# Expansion of phycobilisome linker gene families in mesophilic red algae

**DOI:** 10.1038/s41467-019-12779-1

**Published:** 2019-10-23

**Authors:** JunMo Lee, Dongseok Kim, Debashish Bhattacharya, Hwan Su Yoon

**Affiliations:** 1 Department of Biological Sciences, Sungkyunkwan University, Suwon, 16419 Korea; 2 Department of Biochemistry and Microbiology, Rutgers University, New Brunswick, NJ 08901 USA; 3 Present Address: Department of Oceanography, Kyungpook National University, Daegu, 41566 Korea

**Keywords:** Genome evolution, Molecular evolution, Phylogenetics, Comparative genomics

## Abstract

The common ancestor of red algae (Rhodophyta) has undergone massive genome reduction, whereby 25% of the gene inventory has been lost, followed by its split into the species-poor extremophilic Cyanidiophytina and the broadly distributed mesophilic red algae. Success of the mesophile radiation is surprising given their highly reduced gene inventory. To address this latter issue, we combine an improved genome assembly from the unicellular red alga *Porphyridium purpureum* with a diverse collection of other algal genomes to reconstruct ancient endosymbiotic gene transfers (EGTs) and gene duplications. We find EGTs associated with the core photosynthetic machinery that may have played important roles in plastid establishment. More significant are the extensive duplications and diversification of nuclear gene families encoding phycobilisome linker proteins that stabilize light-harvesting functions. We speculate that the origin of these complex families in mesophilic red algae may have contributed to their adaptation to a diversity of light environments.

## Abstract (web summary)

Widely distributed red algae have experienced massive genome reduction during evolution. Here, using an improved genome assembly of *Porphyridium purpureum*, Lee et al. show the role of endosymbiotic gene transfer in plastid evolution and the correlation between phycobilisome linker diversification and the red algal radiation.

## Introduction

Intracellular gene transfer from permanent endosymbionts (e.g., plastids and mitochondria) to the host nuclear genome is referred to as EGT and has been widely reported in photosynthetic eukaryotes^1,2^. Analysis of the shared set of EGTs in the Archaeplastida (comprising Rhodophyta, Glaucophyta, and Viridiplantae) common ancestor may reveal evolutionary events that occurred when a heterotrophic eukaryotic host captured and retained a cyanobacterial cell. For example, the three Archaeplastida taxa share a plastid protein import system whereby nuclear-encoded, plastid-destined proteins contain a pre-sequence that targets them to the organelle via translocons at the inner/outer chloroplast (plastid) membranes (TIC/TOC). This conserved nanomachine was established during the early stages of endosymbiosis (i.e., before diversification into the three algal lineages) by the ancient EGT events^3–5^. However, differential EGTs and independent gene losses that occurred after Archaeplastida diversification resulted in widely different gene inventories not only in plastid DNA but also in the nuclear genomes of algae and plants^6–8^.

One of the most prominent cases of ancient EGT followed by divergence among Archaeplastida involves the light-harvesting antenna complex, which absorbs and transfers light energy to chlorophyll-*a* in photosystem II (PS II)^9,10^. The cyanobacterial plastid donor had a variety of light-harvesting strategies using phycobiliproteins (phycoerythrin (PE), phycocyanin (PC), and other accessary pigments) organized in macromolecular complexes referred to as phycobilisomes (PBSs) that have broad spectral properties^11,12^. In contrast, primary plastids display a different composition of the major antenna pigments: i.e., PBSs in the Rhodophyta and Glaucophyta, and light-harvesting chlorophyll *a*/*b* proteins in Viridiplantae (green algae and land plants)^9,10^. The red algal light-harvesting antenna complex, with considerable modifications, was spread via secondary/tertiary plastid endosymbiosis to a vast array of marine primary producers such as Cryptophyta, Haptophyta, Heterokontophyta, and Dinophyta^9,13,14^.

In red algae, PBSs are the major light-harvesting antenna complexes anchored to thylakoid membranes^10^. This protein complex is composed of PE, PC, and allophycocyanin (APC) and its structure and organization has been elucidated from the unicellular red alga *Porphyridium*^10,15,16^. For instance, in *Porphyridium cruentum* (a synonym of *Porphyridium purpureum*; www.algaebase.org), the basis of functional stability across pH 4–8 has been explained by pH-dependent structural conformations of the B-PE protein complex^17,18^. Based on 3D protein structure models, highly resolved assembly mechanisms and energy transfer pathways of PBSs have been studied from the red alga *Griffithsia pacifica*^19^. Zhang et al.^19^ characterized not only protein subunits of the antenna pigments but also the linker proteins that play important roles in the formation of PBSs by connecting rods (i.e., PC and PE) and core (i.e., APC) structures. They classified rod linker proteins into three classes: (1) LR1–LR3 proteins containing the PBS linker domain (pfam00427), (2) LR-gamma4–LR-gamma8 comprising the PE gamma chain linker polypeptide containing the conserved chromophore binding structure, and (3) the LR9 protein containing the adhesion domain FAS1. The rod-core linkers (LCs) consist of LRC1–LRC6 proteins that directly link to the core structure composed of three APC trimers, LC, and core-membrane linker (LCM) proteins^10,19^. Although a diversity of antenna pigments of PBSs have been studied at the sequence-level^20–23^, linker protein families remain poorly characterized in the major subphyla of red algae comprising the Cyanidiophytina (species-poor extremophilic taxa) and two mesophilic lineages, the Proteorhodophytina (unicellular/filamentous algae, including those with intron-rich plastid genomes) and Eurhodophytina (macroscopic seaweeds)^24^. This paucity of data reflects two factors: (1) the very similar domain compositions that make linker proteins difficult to distinguish from each other, and (2) the cyanobacteria-biased functional database (e.g., KEGG) that make it challenging to generate detailed knowledge about algal homologs. An additional aspect to keep in mind is that red algal lineages underwent genome reduction in their last common ancestor^25,26^. Selection for gene loss resulted in the shedding of ca. 25% of the red algal inventory shared with the Viridiplantae, with an additional 18% lost in the ancestor of the Cyanidiophytina^27^. The impact of these events on red algal evolution remains to be fully understood.

Here, we generate an improved long-read hybrid genome assembly (22 Mbp, 52 contigs, N50 = 1.8 Mbp; Illumina and Nanopore technologies) and gene models (9898 predicted proteins) from the model unicellular red alga *P. purpureum* CCMP1328. Based on available red algal genome data, we report several anciently derived EGTs in the Archaeplastida ancestor and linker protein families associated with PBSs. We determine the origins of PBS linker protein families including previously undescribed linker proteins in the mesophilic red algae. Although these taxa show different cellular life styles and plastid genome structures, they share a diversity of conserved, lineage-specific nuclear-encoded PBS linker protein families. Based on these data, we speculate that the ancestor of the mesophilic lineages was under selection to adapt to widely differing light environments through the development of flexible PBS structures.

## Results

### Hybrid genome assembly and the gene models of *P*. *purpureum*

We generated a *P. purpureum* CCMP1328 genome assembly^22^ by incorporating long-read sequencing data (1.0 Gbp; 138,851 reads; N50 of the sequencing reads = 14.9 kbp; Nanopore, Oxford Nanopore technologies, Oxford, UK). The hybrid assembly made using MaSuRCA (v3.2.8)^28^ relied on the Nanopore data with existing Illumina sequencing reads from *P. purpureum* CCMP1328 (accession: SRX242705)^22^. This assembly was 22.1 Mbp in size and contains 52 contigs with N50 = 1.8 Mbp, with the largest contig being 5.7 Mbp. This is a significant improvement over the existing short-read based *P. purpureum* genome assembly (150 × 150 bp Illumina MiSeq library; 19.7 Mbp assembly: 4770 contigs, N50 = 20 kbp)^22^. We analyzed repeated sequences in the assembly using RepeatModeler (see Methods) and found a total of 14% repeated DNA in the genome, which is greater than previously reported (4%)^22^. Among the repeated sequences, long terminal repeat (LTR) elements are the most abundant (1.8 Mbp or 8% of the genome; Supplementary Table 1). The Kimura evolutionary distances^29^ between repeated sequences show the accumulation of “unknown” repeats and a relatively recent expansion of LTR elements (Supplementary Fig. 1). We predicted 9898 gene models using published RNA sequence data from *P. purpureum* (SRX242707), available red algal nuclear proteins^22,26,30–33^, and red algal expression sequencing tags (EST)^34,35^ data (Supplementary Table 2; details in Methods). Benchmarking Universal Single-Copy Orthologs (BUSCO) analysis^36^ showed that the gene models encompass 90.4% of the 429 conserved eukaryotic BUSCO gene set, which is the highest value among currently available red algal genomes (Supplementary Table 3).

Comparison of the existing^22^ and newly generated assemblies turned up 3.1 Mbp of unique DNA sequences (BLASTn *e*-value = 0; secondarily sorted from BLASTn results with a 1.e−10 cutoff) in the current data that include newly assembled regions as well as predicted repeated sequences (Supplementary Fig. 2). Although there is 1.0 Mbp of unique DNA sequences in the previous assembly, most of these regions are potential assembly errors in short-read based sequencing data. Based on BLASTp results (*e*-value cutoff = 1.e−20), we found 775 unique protein sequences from the currently predicted gene models that includes 120 genes with low similarity compared to previous gene models (1.*e*−20 < *e*-value < 1.e−05; Supplementary Fig. 2). Although only 62 of the newly described genes were localized to KEGG metabolic pathways, these include 23 uncategorized groups primarily involved in Spliceosome (ko03040), Ribosome (ko03010), and Metabolic pathway (ko01100) functions (Supplementary Data 1). There are also 36 unique protein sequences in the previously predicted gene models^22^, but most of these genes share overlapping frames of different lengths (11), low sequence identities (10), or are plastid genes (7). We also found four tandemly duplicated cryptochrome genes that were previously split (Supplementary Fig. 3). Therefore, the current genome data from *P. purpureum* show many improvements, in particular with respect to measures of genome quality and completeness (Supplementary Table 4).

**Fig. 1:**
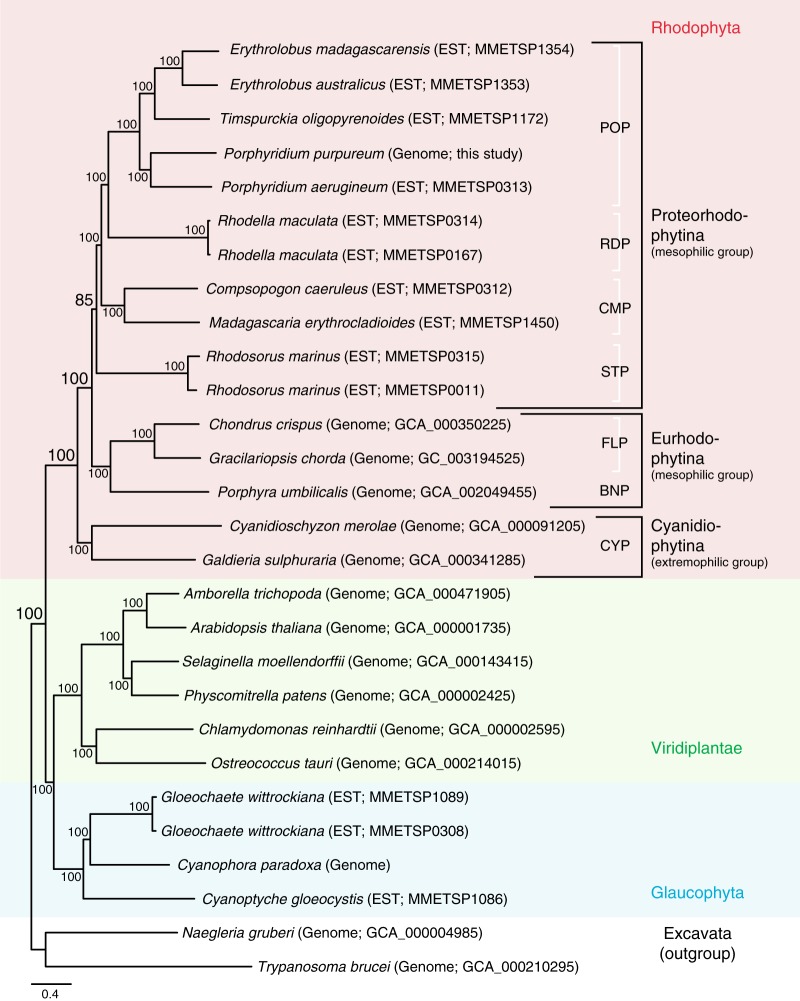
Maximum likelihood tree built using 4777 concatenated Archaeplastida nuclear proteins from whole-genome and EST data. POP Porphyridiophyceae, RDP Rhodellophyceae, CMP Compsopogonophyceae, STP Stylonematophyceae, FLP Florideophyceae, BNP Bangiophyceae, CYP Cyanidiophyceae. Source data are provided as a Source Data file

We constructed a phylogenetic tree of Archaeplastida lineages based on a concatenated dataset of 4777 nuclear proteins (excluding those involved in photosynthesis) that are shared by at least 10 species among the 28 sampled taxa. The phylogeny was inferred using the maximum likelihood (ML) method with 1000 bootstrap replications, using the best-fit evolutionary model (Supplementary Table 2; IQ-tree v1.6.7)^37^. This ML tree provides strong bootstrap support (BS) for a monophyletic Rhodophyta that is sister to the monophyletic groups, Viridiplantae and Glaucophyta (BS = 100%, Fig. 1). The interrelationships of these three primary endosymbiosis groups (i.e., the early split of Rhodophyta) is also supported by transcriptome-based gene and taxon-rich datasets^38^ as well as by phylogenetic analysis of plastid genome data^8^. Within Rhodophyta, the subphylum Proteorhodophytina is well supported (BS = 85%) with inclusion of the Stylonematophyceae and Rhodellophyceae, and the clade of Porphyridiophyceae and Compsopogonophyceae. Although most of these internal relationships have strong BS in this analysis, branching order within the subphylum Proteorhodophytina conflicts with a recent plastid genome-based phylogeny^24^. This discrepancy between nuclear and plastid genome trees may be explained by cryptic hybridization and gene flow between genomes in the ancestor(s) of Proteorhodophytina. A similar case of incongruent phylogenetic signal was recently reported between nuclear rRNA, mitochondrial, and plastid genome data from the Corallinophycidae (Florideophyceae, Rhodophyta)^39^. Additional phylogenetic analyses using a broader sample of red algal nuclear genome data are needed to address their complex evolutionary history.

### Antenna complex in red algal PBSs

**Fig. 2:**
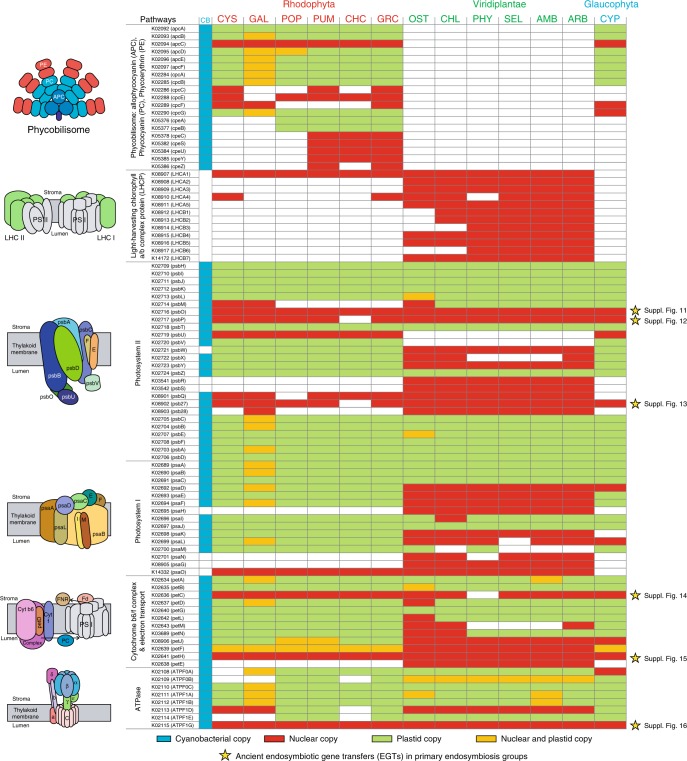
Photosynthetic and the light-harvesting antenna complexes in the primary plastid group based on KEGG database. Nuclear (filled red), plastid (filled green), and both (filled orange) copies in the primary plastid group, and in cyanobacteria (filled cyan) are indicated. Asterisk marks (filled yellow) are indicated as ancient endosymbiotic gene transfers. Entry accessions of KEGG database: map00195 and map 00196. CB Cyanobacteria, CYS *Cyanidioschyzon merolae*, GAL *Galdieria sulphuraria*, POP *Porphyridium purpureum*, PUM *Porphyra umbilicalis*, CHC *Chondrus crispus*, GRC *Gracilariopsis chorda*, OST *Ostreococcus tauri*, CHL *Chlamydomonas reinhardtii*, PHY *Physcomitrella patens*, SEL *Selaginella moellendorffii*, AMB *Amborella trichopoda*, ARB *Arabidopsis thaliana*, and CYP *Cyanophora paradoxa*. Source data are provided as a Source Data file

**Fig. 3:**
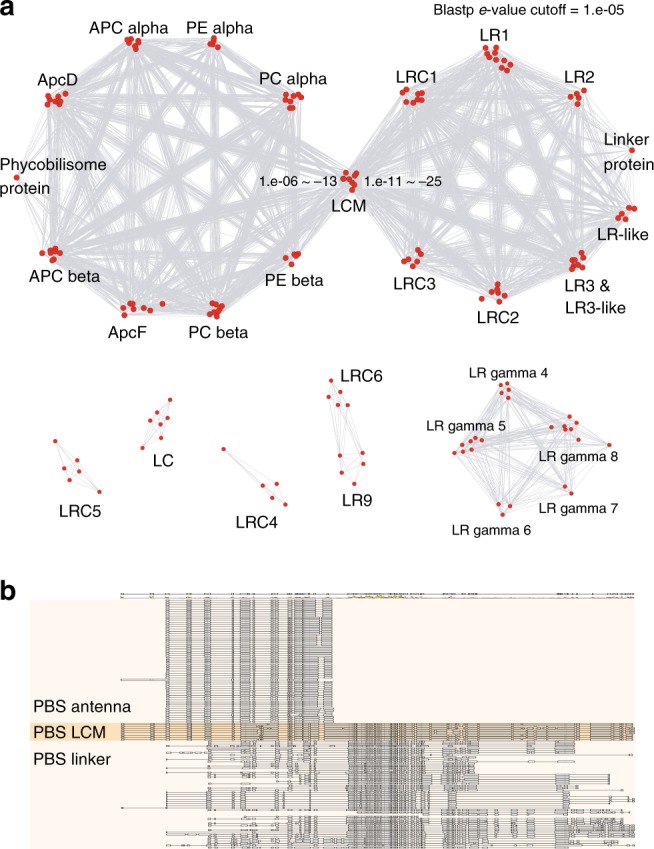
Network analysis of LCM and related proteins in red algae. **a** Protein similarity-based network of red algal phycobilisome families (Blastp *e*-value cutoff = 1.e−05; drawn using Cytoscape). **b** Alignment of phycobilisome LCM-related genes that were used in the network and phylogenetic analyses

**Fig. 4:**
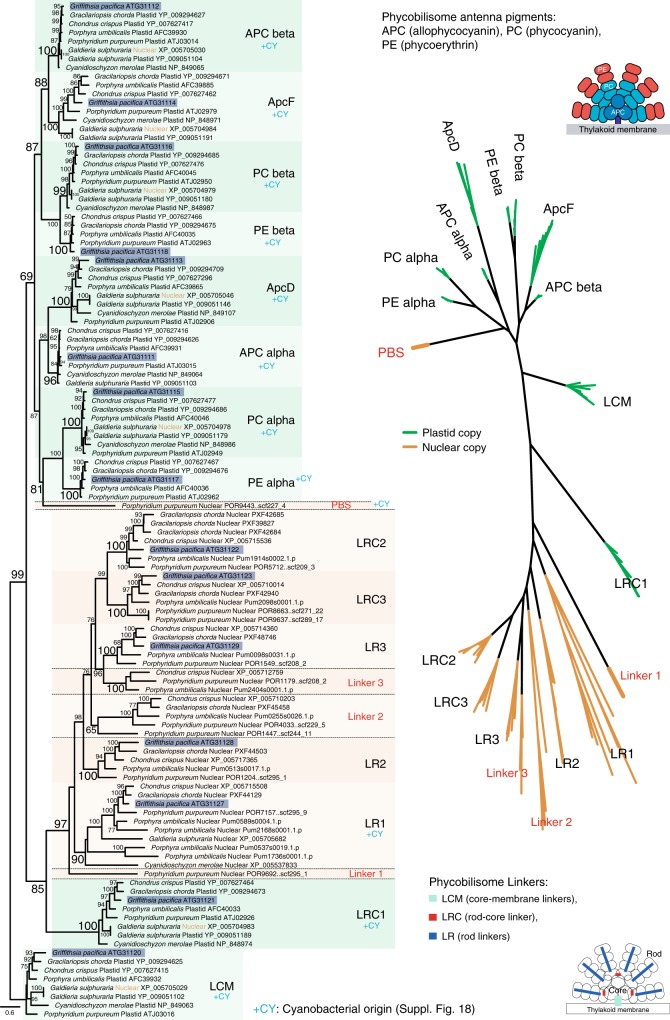
Phylogenetic relationship of all phycobilisome antenna pigments and homologous phycobilisome linker proteins portrayed as rooted and un-rooted ML trees. The core-membrane linker LCM was chosen as outgroup of the rooted tree because it comprises a link between antenna pigments and other linker proteins (Fig. 3). Each family is defined by the phylogenetic cluster based on the reference *Griffithsia pacifica* (dark-blue colored blocks)^19^. Each clade in the unrooted tree shows plastid (green color) and nuclear-encoded (orange color) copies of these families with simplified structural schemes of the phycobilisomes. Source data are provided as a Source Data file

In red algae (as in glaucophytes^40^), the majority of the components of the photosynthetic machinery is encoded in the plastid genome, rather than in the nucleus. In contrast, over one-half of the photosynthetic machinery is nuclear-encoded in green algae and land plants (Fig. 2). These differences are explained by differential EGT or outright gene loss in the ancestors of each lineage^8,27^. To generate a more complete picture of the gene loss process, we analyzed EGTs that putatively (i.e., excluding parallel transfers) occurred before the diversification of the three Archaeplastida lineages (Fig. 2 and Supplementary Fig. 4). This analysis shows that genes with several core plastid functions including the TIC/TOC complex and several involved in photosynthesis (PSII, cytochrome, ferredoxin, and ATPase complexes) were moved to the nuclear genome of primordial algae (Supplementary Figs. 5–16; see details in Supplementary Note 1). Duplication and divergence are found in gene families that encode the light-harvesting antenna complexes, including PBSs in red algae and glaucophytes, and light-harvesting chlorophyll *a*/*b* proteins in Viridiplantae^9,10^. Phycobilisomes were likely lost in the ancestor of green algae and land plants because these protein complexes are present both in glaucophytes and rhodophytes. The phycobilisomes are more highly diverged in red algae, suggesting independent diversification of these protein components after the split from Glaucophyta, which contains a limited phycobilisome machinery. Glaucophytes encode a small number of phycobilisome linker proteins, although this insight comes from a single completed nuclear genome, that of *Cyanophora paradoxa* (Figs. 1 and 2)^40,41^. We characterized red algal phycobilisome protein families using a protein similarity-based network method (Blastp; *e*-value cutoff = 1.e−05), conserved domains (CD-search)^42^, and phylogenetic analysis (IQ-tree)^37^, relying on the well-characterized reference protein sequences from *G*. *pacifica* (details in Methods)^19^. This analysis included not only the antenna pigment-proteins (PC, PE, and APC) but also all rod linkers, rod-LCs, and core structure-related proteins. Based on the network analysis, six groups were identified with one dominant connected component that includes all phycobilisome antenna pigments and major linker proteins. This component contains a central node comprising LCMs that connects the LR (rod linker) and LRC (rod-LC) protein families (Fig. 3; see details in Methods). The LCMs include sequences homologous with the phycobilisome antenna and other linker proteins (Fig. 3a, *e*-value cutoff = 1.e−05). These network connections are also present when using a more stringent cutoff (*e*-value cutoff = 1.e−10, Supplementary Fig. 17). The PBS antenna proteins align well with the N-terminal region of LCMs, whereas linker proteins (i.e., LR and LRC) share similarity with the C-terminus of LCMs (Fig. 3b). Although aligned amino acid sequences between the PBS antenna and linker proteins show low overall similarity (*e*-value > 1.e−05), these two antenna and linker proteins share several well-conserved amino acid residues (Fig. 3b). Therefore, we used the alignment to construct the phylogeny of LCM-related genes to study the relationships of red algal PBS protein families (Fig. 4).

All antenna pigments, including APC, PC, and PE are generally plastid-encoded in photosynthetic red algae (green branches in Fig. 4). However, there are a few exceptions with regard to duplicated copies of these genes in the nuclear genome of *Galdieria sulphuraria* and a previously undescribed nuclear-encoded plastid-targeting PBS protein in *P*. *purpureum* (see color-code in Fig. 4). In addition, PBS of Cyanidiophytina lack PE that is absent not only in genome data (PE alpha and beta in Fig. 4) but also when studied using fluorescence spectroscopy^43,44^. The previously undescribed protein in *P. purpureum* clustered with PBS subunits but does not belong to an existing family (PBS in Fig. 4). Although this protein was reported in an earlier study of the *P. purpureum* genome^22^, we extend this result by showing that it is present in other red algae and in some cryptophytes (Supplementary Fig. 18). Because of the well-supported relationship with cyanobacteria that contain APC-like globin domains (cd12130), this PBS protein is likely to be involved in APC-related functions (Supplementary Fig. 18) and contains a globin superfamily-like domain superfamily (cl21461). Homologs of the previously undescribed PBS protein are present only in the subphylum Proteorhodophytina (i.e., *Erythrolobus* spp., *P. purpureum*, *Rhodosorus marinus*; Supplementary Fig. 18), despite the fact that our database includes whole-genome data from members of the subphylum Eurhodophytina (i.e., *Porphyra umbilicalis*, *Gracilariopsis chorda*, *Chondrus crispus*) and Cyanidiophytina (i.e., *Cyanidioschyzon merolae*, *Galdieria sulphuraria*) species (Fig. 1). Moreover, only cryptophyte nuclear-encoded proteins (*Guillardia theta* XP_005827057 and ESTs from *Hemiselmis andersenii* and *Chroomonas mesostigmatica*) are identified in this family within the red alga-derived secondary endosymbiosis groups. Blastp (*e*-value cutoff = 1.e−05) analysis using the nr database returns the same result. It is, however, still unclear if cryptophyte APC is fully functional because it has not been reported from these taxa^9,45–47^.

### Origin and diversification of PBS linker proteins

Phycobilisome linker proteins play important roles in the structure of PBS antenna pigments and linkage to the thylakoid membrane^10,19^. Despite their well-studied protein structural assembly mechanisms^10,15,16,19^, the origin of red algal PBS linker families is poorly understood. This is because they share similar domain compositions, making it challenging to identify individual family members using sequence data. Furthermore, the KEGG PBS database is not a good predictive tool because it is primarily based on cyanobacterial genome data and lacks information from diverse red algal and glaucophyte PBS linker proteins (Figs. 2 and 4)^19,40^. To overcome these hurdles, we used phylogenetic approaches to categorize PBS linker protein families and used the well-characterized *G*. *pacifica* PBSs^19^ as the reference for this approach (details in Methods).

Based on the phylogenetic analyses, we categorized red algal PBS linker proteins into nine groups. Among them, the LCM and LRC1 (rod-LC 1) are encoded in plastid genomes, similar to their antenna pigments (e.g., APC, PC, and PE), whereas the remaining linker proteins are encoded in the nuclear genome (Fig. 4). Each nuclear-encoded PBS linker protein family (Nu-PBS linker), LR1–LR3, LRC2, and LRC3 form well-supported monophyletic groups. The Nu-PBS linker proteins (BS = 97%) are closely related to the plastid-encoded linker protein LRC1 (Fig. 4). From this analysis, we found three Nu-PBS linkers in *P. purpureum*. The previously undescribed linker 1 is closely related to the LR1-clade and contains only a single predicted PBS linker domain (cl27695) that differs from most LR1 proteins that include not only a specific PBS linker polypeptide domain (pfam00427) but also a specific APC linker domain (pfam01383; Fig. 4).

The linker 2 contains a PBS linker domain, however, the characterized PBS linker protein from *G*. *pacifica* is absent and two Nu-PBS linkers of *P. purpureum* are distinct within the clade (BS = 65%; Fig. 4). The LR3 clade contains duplicated copies from an independent family that includes the linker 3 protein that is present in three red algal species (Fig. 4). The two rod-LC proteins, LRC2 and LRC3, are likely independently derived from the LR3-related rod linker protein of the plastid-encoded rod-LC (i.e., LRC1; Fig. 4). Interesting, with the exception of the LR1 family that contains Cyanidiophytina, other Nu-PBS linker families are present only in mesophilic red algae (i.e., Proteorhodophytina and Eurhodophytina). These Nu-PBS linker families likely diversified after the basal split of Cyanidiophytina.

### Origin of PBS linker proteins

To better understand the origin of Nu-PBS linker proteins, we added homologs from a broader sampling of taxa, including cyanobacteria (Supplementary Fig. 18). Plastid genome-encoded red algal LCM and LRC1 proteins clearly grouped with cyanobacteria and glaucophytes. Nuclear-encoded LR1 clustered with cyanobacteria (BS = 95%), within the monophyletic clade (BS = 99%) of the remaining Nu-PBS linker proteins (Supplementary Fig. 18). This suggests that red algal linker proteins (i.e., LCM, LRC1, and LR1) were derived from cyanobacteria via primary endosymbiosis. Thereafter, the LR1 gene was transferred to the nuclear genome giving rise to the related PBS antenna-homologous linker families. After this EGT event, the remaining red algal Nu-PBS linker proteins (i.e., LR2, LR3, LR3-like, LR-like, LRC2, and LRC3) diversified in the Proteorhodophytina and Eurhodophytina, after the split of the Cyanidiophytina. Only the Nu-PBS linker 1 protein family is limited to Proteorhodophytina species (i.e., *Erythrolobus* spp., *Compsopogon caeruleus*, *P. aerugineum*), suggesting an independent diversification in this subphylum.

With the exception of the major connected component in the network analysis, the other five components comprising Nu-PBS linker proteins include the LC (APC trimers LC), the homologous LR gamma 4–8 group, LRC4, LRC5, and the homologous LRC6–LR9 (Fig. 3). As the APC LC protein, the red algal LC family originated from cyanobacteria and these are also present in glaucophyte algae (Supplementary Fig. 19). The homologous LR gamma linker proteins are categorized into separate clades that are present in Proteorhodophytina (LR gamma 5 and 8) and Eurhodophytina (all LR gamma family members; Supplementary Fig. 20a), however, their origins are unclear (Supplementary Fig. 20b). The LRC4 and LRC5 families are apparently red algal-specific proteins, and only LRC4 includes a single Cyanidiophytina species (i.e., *Galdieria sulphuraria*; Supplementary Fig. 21).

Interestingly, the LRC6–LR9 families in red algae are grouped with diverse eukaryotes (Supplementary Fig. 22). Although the functions of these proteins in red algae involve PBS linkage structure^19^, homologs in non-photosynthetic eukaryotes have extracellular functions related to cell–cell interaction and cell adhesion [e.g., *Homo sapiens* (Uniprot ID: Q9NY15, Q8WWQ8), *Drosophila melanogaster* (Q8IP52, Q86B94), and *Mus musculus* (Q8R4Y4, Q8R4U0)] (Supplementary Fig. 22). The homologs in Cyanidiophytina are distantly related to the clade of mesophilic red algae, suggesting independently derived functions.

## Discussion

**Fig. 5:**
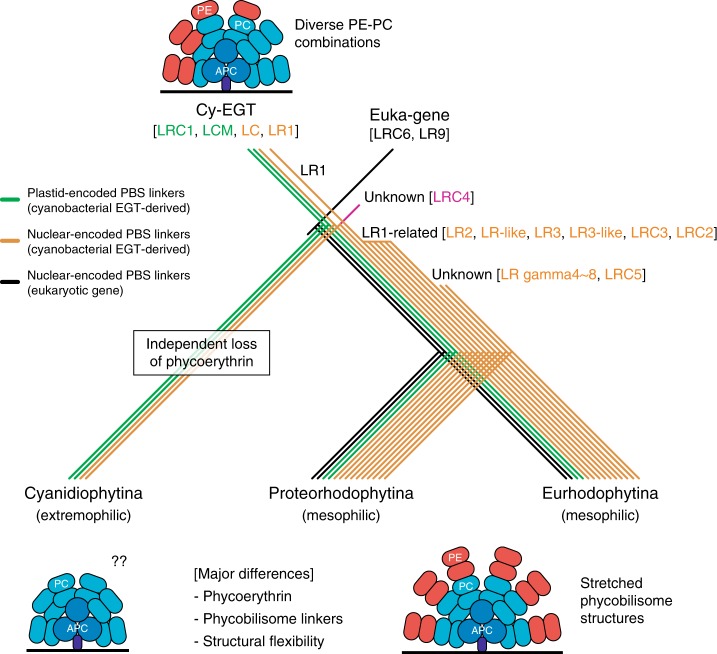
The differing diversification patterns of red algal phycobilisome linker proteins when comparing extremophilic and mesophilic red algae. Cy-EGT and Euka-gene are cyanobacterium-derived EGTs and eukaryotic genes, respectively (Supplementary Data 2). The colored branches are the Cy-EGT-derived plastid-encoded phycobilisome (PBS) linkers (green color), nuclear-encoded PBS linkers (orange color), and eukaryotic nuclear-encoded PBS linkers (black color). The divergence of LRC4 family is unclear because of the existence of a Cyanidiophytina gene copy (Supplementary Fig. 21)

Our study identified ancient EGTs in Archaeplastida that gave rise to a diversity of PBS-linker protein families in mesophilic red algae. These proteins contributed to the establishment of photosynthetic components during the early phases of primary endosymbiosis and to the stability of PBS structures. Our results provide insights into the evolutionary history of PBS structures in mesophilic red algae (i.e., subphylum Proteorhodophytina and Eurhodophytina), in which linker protein diversification occurred after the EGT-derived origin of a cyanobacterium-derived LR1 encoding gene (Fig. 5, Supplementary Fig. 18 and Supplementary Data 2)^10,15,19,48^. Red algal linker protein families are however derived from multiple sources, including cyanobacteria (EGT and plastid encoded; Cy-EGT), eukaryotic genes (Euka-gene), as well as unknown (red algal-specific) origins that underwent expansion (e.g., LR1-related linker families; Fig. 5). These patterns are distinct from the PBS-containing Glaucophyta. The model glaucophyte *Cyanophora paradoxa* contains a limited PBS machinery (i.e., *apc*A-F, *cpc*A-B, and *cpc*F-G) comprising gene duplication-derived PBS linker proteins (Fig. 2 and Supplementary Fig. 18)^40,41^. Among the cyanobacterial PBS-derived eukaryotic lineages (i.e., glaucophytes and rhodophytes), only the mesophilic red algae contain distinct and diversified linker proteins. We speculate that the origin of complex PBS linker families may reflect selection to expand light-harvesting capacity during the radiation of mesophilic red algae (7100 described species) into a variety of aquatic environments such as the high and low intertidal, subtidal, and in association with coral reefs.

Phycobilisome structural mobility in *P. purpuruem* is greater than in *Cyanidium caldarium* (Cyanidiophytina) with regard to the regulation of light-harvesting efficiency^16^, consistent with the transition to the mesophilic habitats. In addition, the B-PE complex of *P. purpureum* shows pH-dependent structural conformations with strong functional stability in a wide pH range^17,18^. Cyanidiophytina lacks PE^43,44^, and their PBS structures may be simpler than in mesophilic taxa (Fig. 5)^10,19^. Because the primary plastid donor (cyanobacteria) presumably contained PE and PC, it is likely that the ancestor of Cyanidiophytina lost PE and its PE-associated linker proteins (Fig. 5)^49^. This event may be explained by the different light environment in many high elevation hot spring habitats and the reliance of many of these taxa on heterotrophic growth that allows them to utilize a variety of external carbon sources (e.g., glucose)^32^. Additional studies are needed of unicellular and multicellular mesophilic red algae to uncover the relationship between structural stability of PBS complexes, resulting from linker protein diversification, and the ability of these taxa to thrive in different light environments (Supplementary Note 2, Supplementary Table 5, and Supplementary Figs. 23–26).

## Methods

### Sample preparation, genome sequencing, and assembly

Samples of the unicellular red alga *P. purpureum* CCMP1328 were subcultured in L1-Si standard medium (NCMA, https://ncma.bigelow.org/) and the cells were harvested using 5 µm pore-sized mixed cellulose ester membrane filters (Advantec MFS Inc., Tokyo, Japan). DNA extraction from *P. purpureum* cells was done using the CTAB method with several DNA purification steps. For sequencing library preparation, we used the standard protocol described in the MinION library preparation kit (SQK-LSK109; Oxford Nanopore Technologies) by following these steps: DNA repair/end-prep without DNA fragmentation step, adapter ligation, and clean-up steps. After priming a new flow cell and loading the prepared sequencing library, the Nanopore sequencing run was progressed during 48 h by the MinKNOW v1.14.1 platform (GUI v2.1.14). The base-calling of raw sequence data was conducted using the Albacore v2.3.1 script provided by Oxford Nanopore Technologies (https://nanoporetech.com). These base-called sequencing raw reads (1.0 Gbp; number of reads: 138,851) were used for genome assembly. The hybrid genome assembly was done by MaSuRCA assembler (v3.2.8)^28^ using the base-called reads from Nanopore sequencing and the published short-read Illumina sequencing data (accession: SRX242705)^22^. Error correction step, gene-modeling, and functional annotations are described in Supplementary Note 3.

### **A**nalysis of repeated elements in the hybrid genome assembly

We analyzed repeated DNA elements based on the RepeatModeler pipeline (v1.0.11; http://www.repeatmasker.org/RepeatModeler) that includes de novo repeat family identification and modeling package (RECON v1.08 and RepeatScout v1.0.5)^50,51^. We used the default l-mer size option and filtered out low-complexity and tandem repeats (Tandem Repeats Finder)^52^. The repeat classifications were conducted based on Repeat Library that was downloaded from the server (http://www.girinst.org). A customized Python script was used to parse the frequency of Kimura distances from the classified repeat elements.

### Gene family analysis

Gene families of our target proteins were defined by functional categories (KEGG accessions). Other un-categorized gene families were defined by phylogenetic analysis and their shared conserved domains based on already defined gene families. For example, the red algal PBS linker protein families were previously reported as unknown protein, but with the detailed functions, 3D structure models, and their protein sequences were described recently from *G. pacifica*^19^. Based on these reference proteins, homology searches were done using Blastp (*e*-value cutoff = 1.e−05), and putative homologs were aligned by MAFFT v7.313 (default option:–auto)^53^. Through phylogenetic analysis of the homologous sequences based on the ML method (1000 replications; IQ-tree v1.6.7)^37^, we defined the gene family based on their monophyletic cluster that grouped with the reference protein. In addition, categorized proteins within the cluster were validated using evidences from conserved domains, if possible to get domain prediction results of the proteins. To analyze origins of a specific gene family, Blastp searches (*e*-value cutoff = 1.e−05) of target proteins were done to our local RefSeq database, and then the top ten hits in each taxonomic group were collected with our target proteins. The collected homologous genes were aligned by MAFFT v7.313 (default option:–auto)^53^, and analyzed by IQ-tree with 1000 replications (v1.6.7)^37^.

### Reporting summary

Further information on research design is available in the Nature Research Reporting Summary linked to this article.

## Supplementary information

Supplementary Information

Peer Review File 

Reporting Summary

Description of Additional Supplementary Files

Supplementary Data 1

Supplementary Data 2

Source Data

## Data Availability

Data supporting the findings of this work are available within the paper and its Supplementary Information files. A reporting summary for this Article is available as a Supplementary Information file. The datasets generated and analyzed during the current study are available from the corresponding author upon request. The hybrid genome assembly, gene models, and functional annotations of *P. purpureum* are available at http://porphyra.rutgers.edu/bindex.php, NCBI (BioProject: PRJNA560054; Genome accession number VRMN00000000), or Marine Bioinformation Center, National Marine Biodiversity Institute of Korea (Raw data: MN00623-0001; Assembly data: MA00293). RNA-seq data are available at NCBI (SRR10010265–SRR10010276). The source data underlying Figs. 1, 2, 4, as well as Supplementary Figs. 25 and 26 are provided as a Source Data file.
