# Supplementary material for: Expansion of phycobilisome linker gene families in mesophilic red algae
Source: Nat Commun. 2019 Oct 23;10:4823. doi: 10.1038/s41467-019-12779-1 (PMC6811547; doi:10.1038/s41467-019-12779-1)
Supplement: Supplementary file 1 — Supplementary Information [file 41467_2019_12779_MOESM1_ESM.pdf]

Expansion of phycobilisome linker gene families in mesophilic red algae

Lee *et al.*

Supplementary Note 1

Ancient EGT in the primary endosymbiosis group

Endosymbiotic gene transfers (EGTs) from permanent endosymbionts (i.e., organelles) to the host nuclear genome have been widely reported in photosynthetic eukaryotes[1,2]. Among these intracellular gene transfers, we focused on the ancient EGTs that occurred before the diversification of the three Archaeplastida lineages (Fig. 2). Several core-plastid functions were moved to the nuclear genomes of primordial algae through ancient EGTs, and may have contributed to the establishment of photosynthesis in primordial algae. Several ancestral features are shared in the primary endosymbiosis group. For example, a phenylalanine (F) residue in the N-terminal sequences of TOC75 translocon is frequently present in rhodophytes and glaucophytes[3-5]. One function involves the TIC/TOC complex[6,7] for which ancient EGTs impacted the proteins TOC75, TIC20, TIC21, and TIC62 (Supplementary Figures 5-8). These are core functional units of the protein-transporting channels through the inner and outer plastid membranes[6,8-10]. Other TIC/TOC proteins are eukaryotic-specific (TOC34 and TIC110; Supplementary Figure 9 and 10)[7,11,12], land plant-specific (TIC55, note that the green alga *Chlamydomonas reinhardtii* contains 'Rieske iron-sulfur cluster 55 kDa protein of the chloroplast inner membrane translocon; XP_001697980)[7,11], and Viridiplantae-specific (TOC159)[11], or have an ambiguous/unknown provenance[7,11,12].

We also found examples of ancient EGTs that involved photosynthetic functions (PSII, cytochrome, and ATPase complexes; Fig. 2). Several instances of ancient EGT shared by all three Archaeplastida groups were found (i.e., these genes are absent from plastid genomes; yellow stars in Fig. 2) and include components of PSII (*psb*O, *psb*P, and *psb*27), the cytochrome complex (*pet*C), the ferredoxin (*pet*H), and the ATPase complex (*atp*G; synonym: *atp*C) (Supplementary Figures 11-16). The *psb*O and *psb*P genes encode the oxygen-evolving enhancer proteins 1 and 2, respectively, that are involved in core functions in PSII including activity, optimization, and the membrane system[13-17]. The *psb*27 gene also plays an important role in photosynthesis, involved in the repair of photodamaged PSII[18-20]. Other cases of ancient EGTs are *pet*C (Rieske iron-sulfur protein in the cytochrome b6/f complex)[21] and *pet*H (ferredoxin: NADP+

oxidoreductase), and *atp*G (ATPase gamma subunit; K02115)[22,23] genes that might have contributed to establishment of photosynthesis in primordial algae. These core genes were also transferred to the nuclear genomes of secondary/tertiary plastid groups with Archaeplastida-derived organelles (Supplementary Figures 11-16).

Several photosynthetic genes (i.e., *psb*R, *psb*S, *psa*H, *psa*N, *psa*G, and *pet*E), present only in Viridiplantae, are involved in PSII, PSI, and the cytochrome complex but these genes (except *pet*E) are absent in cyanobacterial genomes (Fig. 2)[24,25]. Although these genes are important in photosynthetic functions of Viridiplantae, they may not be necessary in rhodophytes and glaucophytes as well as in cyanobacteria[24,25]. Furthermore, the *pet*E (plastocyanin) gene, which is involved in the functioning of the photosynthetic electron transport chain, is Viridiplantae- and cyanobacterial-specific. In red algae, however, cytochrome-*c*6 encoded by the *pet*J gene is the alternative for the plastocyanin[26]. This suggests that the phycobilisome-containing primary endosymbiosis groups (i.e., rhodophytes and glaucophytes) have different evolutionary history from Viridiplantae lineage that uses the light-harvesting antenna complex (i.e., chlorophyll a/b proteins).

Supplementary Note 2

Gene expression patterns of PBS linker proteins

The PBS complex of *P. purpureum* allows this alga to adapt to different light conditions through a variety of light-absorption abilities and energy transfer mechanisms[27,28]. To gain insights into gene expression patterns of PBS linker proteins in *P. purpureum*, we used transcriptomics (RNA-seq) to study PBS linker protein gene expression under limited light conditions. To collect RNA-seq data, we initially cultured *P. purpureum* under white light LED (10 μmol photons m^{-2} s^{-1}) as control for one week, and then exposed the cells to 30 μmol photons m^{-2} s^{-1} (higher) white light, blue light (peak wavelength = 463 nm; 5 μmol photons m^{-2} s^{-1}), and red light (peak wavelength = 628 nm; 5 μmol photons m^{-2} s^{-1}) (Supplementary Fig. 23a; SPIC-200AW, Everfine Co. Hangzhou, China) for five days at 20°C. We used the typical LED white light source as the control without any modification (e.g., decreasing the blue light amount) because

the purpose of our experiment was to determine the gene expression responses of phycobilisome linker protein families when the cells were exposed to the specific wavelengths of red and blue light. We recognize however that it has been found in land plants that the high proportion of blue light in the typical white light LED source can impact gene expression levels resulting in altered phenotypes (e.g., ascorbate content)[27]. All of these experiments used triplicate 50 ml culture tubes. Cell-counting was done before and after the light treatments with the Hemocytometer counting method. Under each light condition, we extracted total RNA using the CTAB method after filtration with a 3 μm pore-sized mixed cellulose ester membrane (ADVANTEC, Japan). The RNA-seq library was constructed using the Truseq Stranded mRNA Prep Kit. The quality of the RNA-seq libraries was checked on a 2100 Bioanalyzer (Agilent Technologies, USA). The amplified RNA-seq libraries were sequenced using 100 bp paired-end reagents with the Illumina NovaSeq 6000 platform. The mapping of RNA-seq data was done with tophat2[28] (Supplementary Table 5), and read counts calculated using HTSeq-count[29]. The scatter plots of HTSeq-count results were made based on Log_{10} (HTSeq-count + 1), and calculated R square (R^2). We used z-score [('Expression' - 'Average expression of all conditions in each gene') / 'Standard deviation of all conditions in each gene'] as normalized values for the gene expression analysis after filtration of moderately expressed genes (maximum / minimum fold change < 1.5-fold). The previously undescribed PBS linker 1 (POR9692..scf295_1), linker 3 (POR1179..scf208_2), and PBS (APC-like; POR9443..scf227_4) genes were filtered in this step because these are moderately expressed genes, therefore their functions are unclear under the tested conditions. In addition, ambiguously expressed genes (< HTSeq-count 100) were also excluded in this analysis.

Algal concentrations increased 1.4 ~ 2.1-fold during the experiment and were roughly similar across all treatments (Supplementary Fig. 23b). Consistent with this results, overall gene expression patterns did not significantly differ between the culture conditions ($R^2 = 0.992 \sim 0.9458$; p-value > 0.05; Wilcoxon rank sum test) based on scatter plots of HTSeq-counts (Supplementary Fig. 24)[29]. These preliminary results suggest that *P. purpureum* grows well under red and blue light. Under higher white light, some light-induced antenna complex genes were more highly expressed than under

lower white light, and under blue and red light (Supplementary Fig. 25a). These higher white light-induced groups include eight phycobilisome proteins and two plastid-encoded PBS linker proteins (LCM and LRC1) that may be related to the quantitative efficiency of photosynthesis. Interestingly, the *cpc*E gene and 10 Nu-PBS linker proteins, including two copies of the previously undescribed linker 2 were down-regulated only under red light, and maintained their expression level under blue light (Supplementary Fig. 25b). We found three potential candidates for the blue light-response vis-à-vis PBS linker proteins based on 2-fold change between blue and red light. LR2 (POR1204..scf295_1), LR3 (POR1549..scf208_2), and LRC5 (POR5765..scf295_1) were down-regulated only under red light (over 2-fold), and maintained their expression level in other conditions (Supplementary Fig. 25). Other potential blue light-response group (1.5 ~ 2-fold between blue and red light) includes PBS core (LC), rod (LR gamma 4, and LR9), rod-core (LRC4, and another LRC5 copy), and previously undescribed (two copies) linkers as well as the phycocyanin lyase alpha subunit (*cpc*E), which catalyzes the attachment of phycocyanobilin and its degradation (i.e., is reversible)[30]. A Nu-PBS linker protein (LR gamma 8) is also a candidate for the blue light response because its expression was maintained under blue light when compared to the control, but is down-regulated in higher light, which contains blue light (Supplementary Fig. 25c). Genes encoding six PBS linker proteins (LR1, LRC2, two of LRC3, and two of LR gamma 4) and a LHCA1 (light-harvesting complex I chlorophyll *a*/*b* binding) protein were marginally down-regulated in higher and blue light and significantly down-regulated under red light when compared to the control (Supplementary Fig. 25d).

Other photosynthetic functions (photosystems, cytochrome, and ATPase complexes) also included higher light-induced and blue light-response genes similar to PBS linker proteins (Supplementary Fig. 26). The 27 genes responding to higher light include all photosynthetic functions (i.e., PSII, PSI, cytochrome, ATPase) mainly encoded in the plastid genome (Supplementary Fig. 26a and 26b). The blue light-response (i.e., down-regulated only under red light) photosynthetic functions (*psa*O, *psb*27, *psb*O, *psb*U, *pet*H, and *pet*C) are nuclear encoded in *P. purpureum* (Supplementary Fig. 26c). The *psb*P and *psb*W genes were highly expressed under

higher and blue light and may also be candidates for the blue light response (Supplementary Fig. 26d). One gene (POR6749..scf209_3) of the two ‘F-type H+-transporting ATPase subunit gamma (ATPF1G)’ genes was up-regulated only in the red light (Supplementary Fig. 26e). Although there was no red light-induced PBS linker protein, the ATPase can be an important role of adaptive mechanism under red light in *P. purpureum*.

Although many PBS linker proteins showed a response to the limited light conditions we tested, no PBS linker gene was significantly up-regulated when compared to the control. We postulate that the organization of PBS liker proteins aids in maintenance of the structural integrity and stability of phycobilisome complexes in mesophilic red algae. To gain greater insight into this process, we suggest comparative genomic, transcriptomic, and biophysical analyses of PBS linker proteins as well as analysis of other photosynthetic pigments in a diversity of *Porphyridium* species.

Supplementary Note 3

Error correction of genome assembly and gene annotations

To construct a more accurate genome assembly, an additional error correction step (i.e., Illumina read mapping and nucleotide corrections) was done using the BWA (Burrows-Wheeler Aligner) program with samtools[31-33]. About 95% of the Illumina sequencing reads were mapped on the 22 Mbp hybrid assembly of *P. purpureum*. The organelle-derived contigs and contaminants (e.g., bacterial genomes) were manually removed by customized python scripts based on BLASTn/p results using the nt/nr database (NCBI). To predict the gene models of *P. purpureum*, we used published RNA sequencing data of *P. purpureum* (SRX242707)[34] and available red algal nuclear proteins from *Cyanidioshcyzon merolae*[35], *Galdieria sulphuraria*[36], *Porphyridium purpuruem*[34], *Porphyra umbilicalis*[37], *Chondrus crispus*[38], and *Gracilariopsis chorda*[39] as well as EST data of *Erythrolobus australicus*, *E. madagascarensis*, *Porphyridium aerugineum*, *Timspurckia oligopyrenoides*, *Compsopogon caeruleus*, *Madagascaria erythrocladioides*, *Rhodella maculata*, and *Rhodosorus marinus*[40,41]. The published RNA-seq data was aligned to our hybrid genome assembly result using STAR aligner

(v2.6.0c)[42]. Based on these mapped RNA-seq data, the gene models were predicted by BRAKER2 pipeline (v2.1.0; http://bioinf.uni-greifswald.de/bioinf/braker)[43], which includes implementation of GeneMark-ET[44] and AUGUSTUS[45]. The homologous gene-based prediction method was also conducted by BRAKER2 pipeline based on the EST data. All these predicted gene models (RNA-seq-based and homology-based) were sorted and combined into the final gene set (9,038 proteins) of *P. purpureum* genome prior to the RNA-seq based gene models. Functional annotations of the gene models in *P. purpureum* were analyzed by CD-search (conserved domain prediction)[46] and KEGG blast (metabolic pathway analysis; http://www.genome.jp/tools/blast). We focused on the analysis of photosynthesis and their antenna proteins (map00195 and map00196) as well as phycobilisome-related conserved domains (Fig. 2 and 4). Plastid-targeting signals were predicted by web-based ChloroP program (v1.1; http://www.cbs.dtu.dk/services/ChloroP; Fig. 4)[47].

Supplementary Table 1. Predicted transposable elements of *Porphyridium purpureum* genome.

Predicted groups	Number	Length (bp)
DNA/Academ	6	15,418
DNA/Crypton	3	1,754
DNA/hAT-Ac	28	38,898
DNA/MULE-MuDR	4	471
DNA/TcMar-Tc1	315	297,345
LINE	61	114,727
LINE/CRE	24	35,927
LINE/CRE-Cnl1	11	9,376
LINE/CRE-II	18	23,466
LINE/I-Jockey	89	15,326
LINE/RTE-X	4	1,040
LTR/Copia	545	982,798
LTR/ERV1	43	53,828
LTR/Gypsy	364	672,306
LTR/Gypsy-Cigr	84	118,372
rRNA	21	71,781
Simple repeat	6,614	349,631
Unknown	1,921	845,981
**Total**	10,155	3,648,445
**Total without overlapping**	-	3,261,703

Supplementary Table 2. Data sources of concatenated genesets for the Archaeplastida phylogeny.

Lineage			Species name	Data	Remarks
**Rhodophyta**	**Cyaniidiophytina**	Cyanodiophyceae	*Cyanidioschyzon merolae*	Genome	GCA_000091205
			Galdieria sulphuraria	Genome	GCA_000341285
	**Proteorhodophytina**	Porphyridiophyceae	*Erythrolobus australicus*	Transcriptome	MMETSP1353
			Erythrolobus madagascarensis	Transcriptome	MMETSP1354
			Porphyridium aerugineum	Transcriptome	MMETSP0313
			Timspurckia oligopyrenoides	Transcriptome	MMETSP1172
			Porphyridium purpureum	Genome	this study
		Compsopogonophyceae	*Compsopogon caeruleus*	Transcriptome	MMETSP0312
			Madagascaria erythrocladioides	Transcriptome	MMETSP1450
		Rhodellophyceae	*Rhodella maculata*	Transcriptome	MMETSP0314
			Rhodella maculata	Transcriptome	MMETSP0167
		Sylonematophyceae	*Rhodosorus marinus*	Transcriptome	MMETSP0315
			Rhodosorus marinus	Transcriptome	MMETSP0011_2
	**Eurhodophytina**	Bangiophyceae	*Porphyra umbilicalis*	Genome	GCA_002049455
		Florideophyceae	*Chondrus crispus*	Genome	GCA_000350225
			Gracilariopsis chorda	Genome	GC_003194525
**Viridiplantae**	Green algae		*Ostreococcus tauri*	Genome	GCA_000214015
			Chlamydomonas Reinhardtii	Genome	GCA_000002595
	Bryophyte		*Physcomitrella patens*	Genome	GCA_000002425
	Pteridophyte		*Selaginella moellendorffii*	Genome	GCA_000143415
	Angiosperms		*Amborella trichopoda*	Genome	GCA_000471905
			Arabidopsis thaliana	Genome	GCA_000001735
**Glaucophyta**	Glaucocystales		*Cyanophora paradoxa*	Genome	Price et al. 2012
			Gloeochaete wittrockiana	Transcriptome	MMETSP1089
			Gloeochaete wittrockiana	Transcriptome	MMETSP0308
			Cyanoptyche gloeocystis	Transcriptome	MMETSP1086
**Excavata (outgroup)**	Heterolobosea		*Naegleria gruberi*	Genome	GCA_000004985
	Kinetoplastea		*Trypanosoma brucei*	Genome	GCA_000210295

Supplementary Table 3. Analysis of BUSCO gene sets using red algal genomes.

Red algal class	Species name	Complete single BUSCOs	Complete duplicated BUSCOs	Fragmented BUSCOs	Missing BUSCOs	Total hits	Remarks
**Porphyridiophyceae**	*Porphyridium purpureum*	366	116	22	41	388 (90.4%)	BUSCO of previous genemodels: 380 (88.5%, Bhattacharya, D. et al. *Nat. Commun.* 2013)
**Cyanidiophyceae**	*Cyanidioschyzon merolae*	313	61	33	83	346 (80.6 %)	
	Galdieria sulphuraria	359	124	21	49	380 (88.5 %)	
**Bangiophyceae**	*Porphyra umbilicalis*	228	55	71	130	299 (69.6 %)	
**Florideophyceae**	*Chondrus crispus*	303	93	40	86	343 (79.9 %)	
	Gracilariopsis chorda	359	100	17	53	376 (87.6 %)	

Abbreviation: BUSCO=Benchmarking Universal Single-Copy Orthologs

Supplementary Table 4. Summarized results of comparative genome analysis between current and previous genome assembly data.

	**Current assembly**	**Previous assembly**
Genome size	22.1 Mbp	19.7 Mbp
Number of contigs	52	4,770
N50 of genome data	1.8 Mbp	0.02 Mbp
Repeated sequences	14%	4%
Unique DNA sequences (BLASTn *e*-value cutoff= 0)	3.1 Mbp	1.0 Mbp
Gene models	9,898	8,355
Coverage of 429 BUSCO gene sets	90.40%	88.50%
Unique protein sequences (BLASTp *e*-value cutoff = 1.*e*-20)	775	36

Supplementary Table 5. Information of RNA-sequencing data.

RNA-seq conditions	Data (bp)	Mapped reads (%)
Control 1 (white light)	5,492,021,652	90.3%
Control 2 (white light)	5,790,459,280	91.1%
Control 3 (white light)	6,142,935,948	92.8%
High light 1	6,402,106,998	90.2%
High light 2	5,734,160,264	91.0%
High light 3	5,439,137,446	87.5%
Blue light 1	5,351,547,822	86.1%
Blue light 2	5,024,918,468	88.7%
Blue light 3	5,122,937,554	90.1%
Red light 1	5,607,138,826	91.6%
Red light 2	5,531,612,036	90.9%
Red light 3	6,442,752,226	91.7%

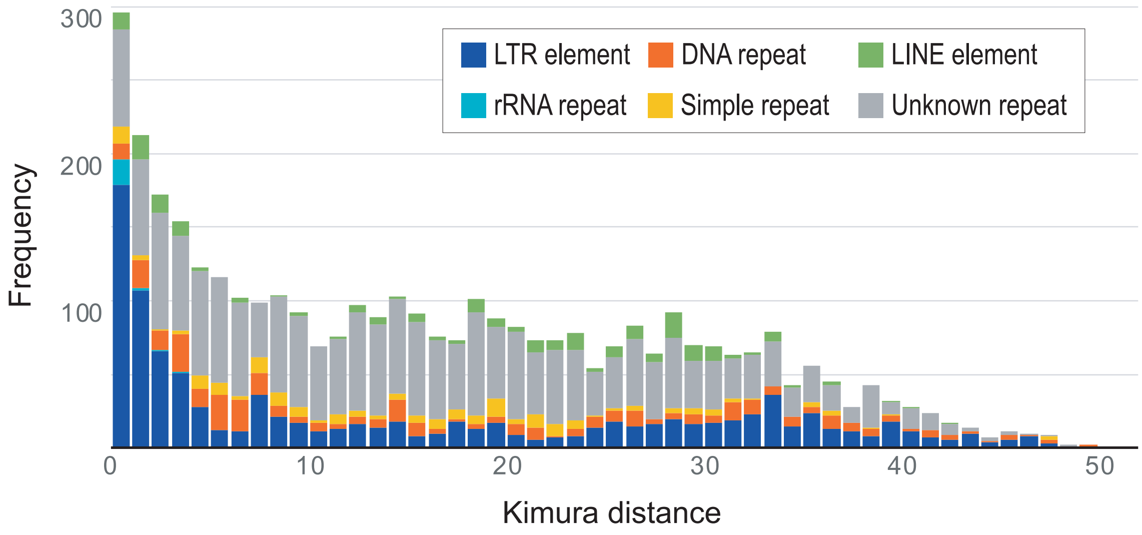

Supplementary Figure 1. Distribution of Kimura values in repeated elements in the *Porphyridium purpureum* genome.

BLASTn comparison

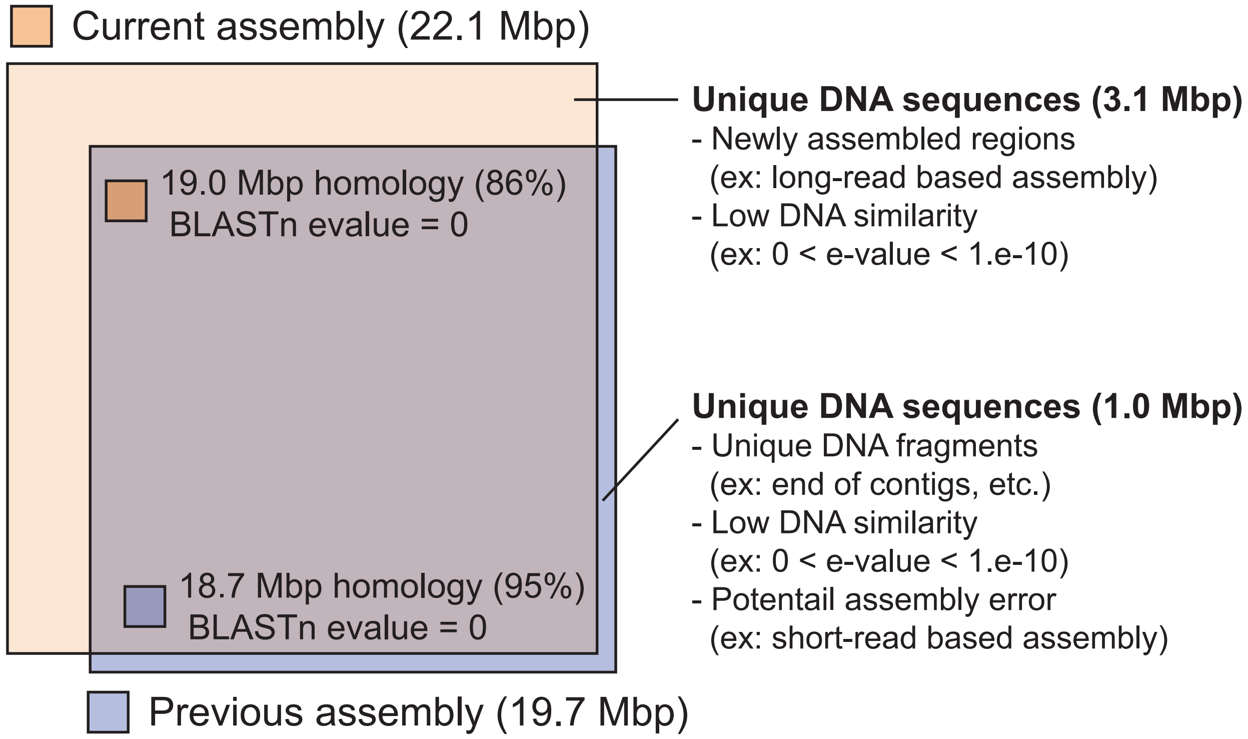

BLASTp comparison

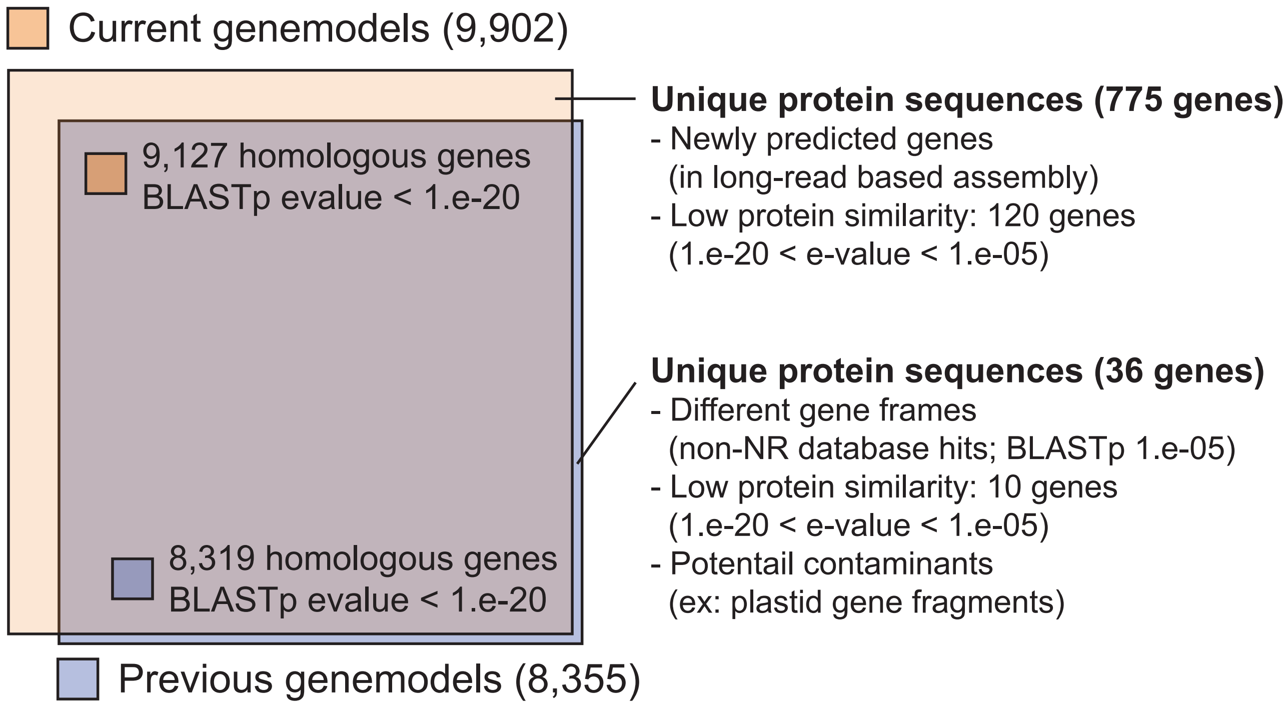

Supplementary Figure 2. Comparisons of assemblies and gene models between current and previous genome data of *P. purpureum*.

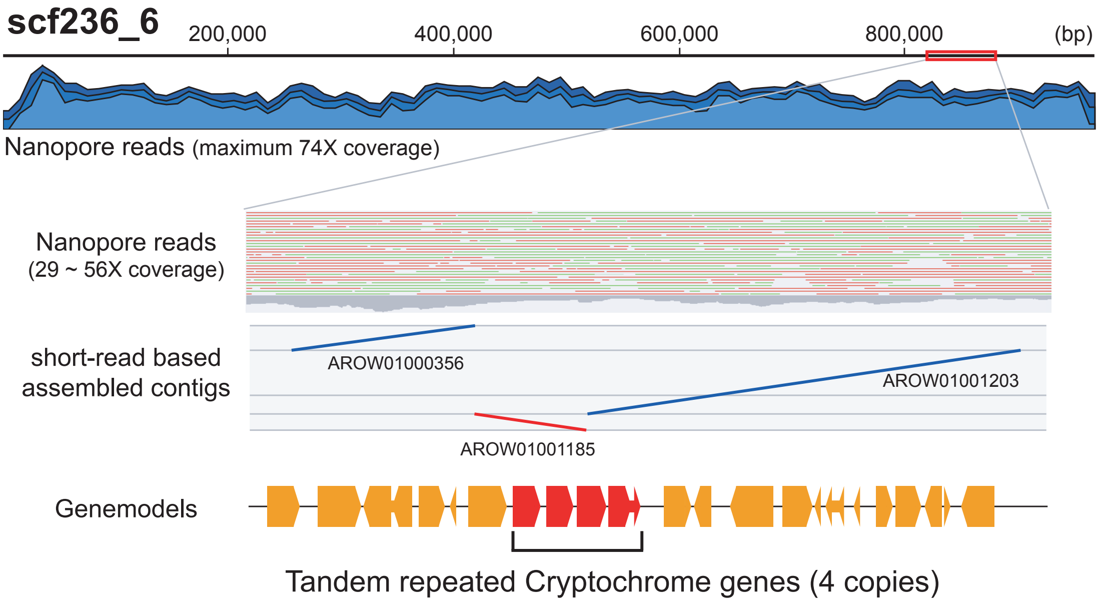

Supplementary Figure 3. Tandem repeated cryptochrome genes of *P. purpureum* and their genomic regions.

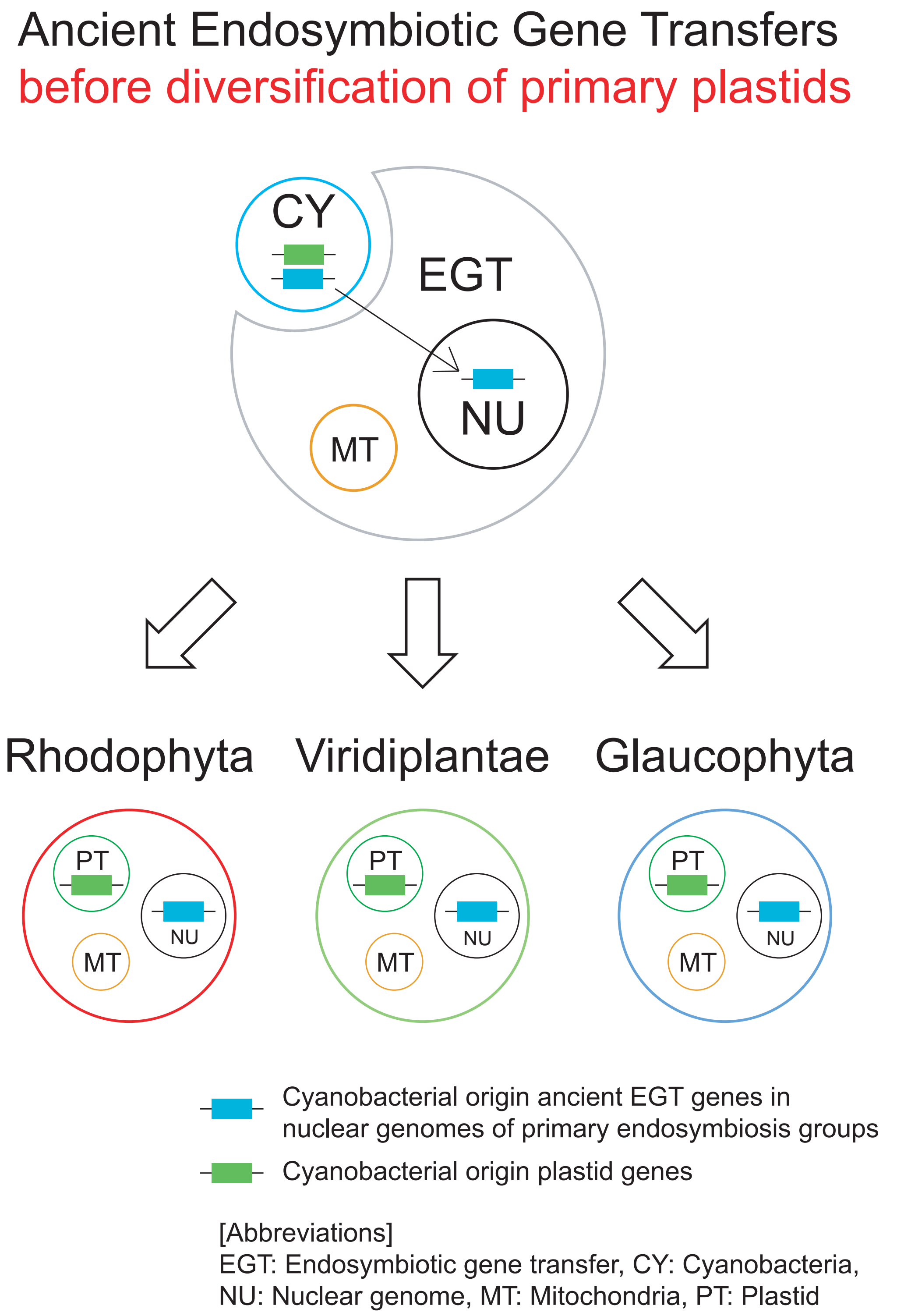

Supplementary Figure 4. Schematic process of ancient endosymbiotic gene transfers before diversification of primary plastids.

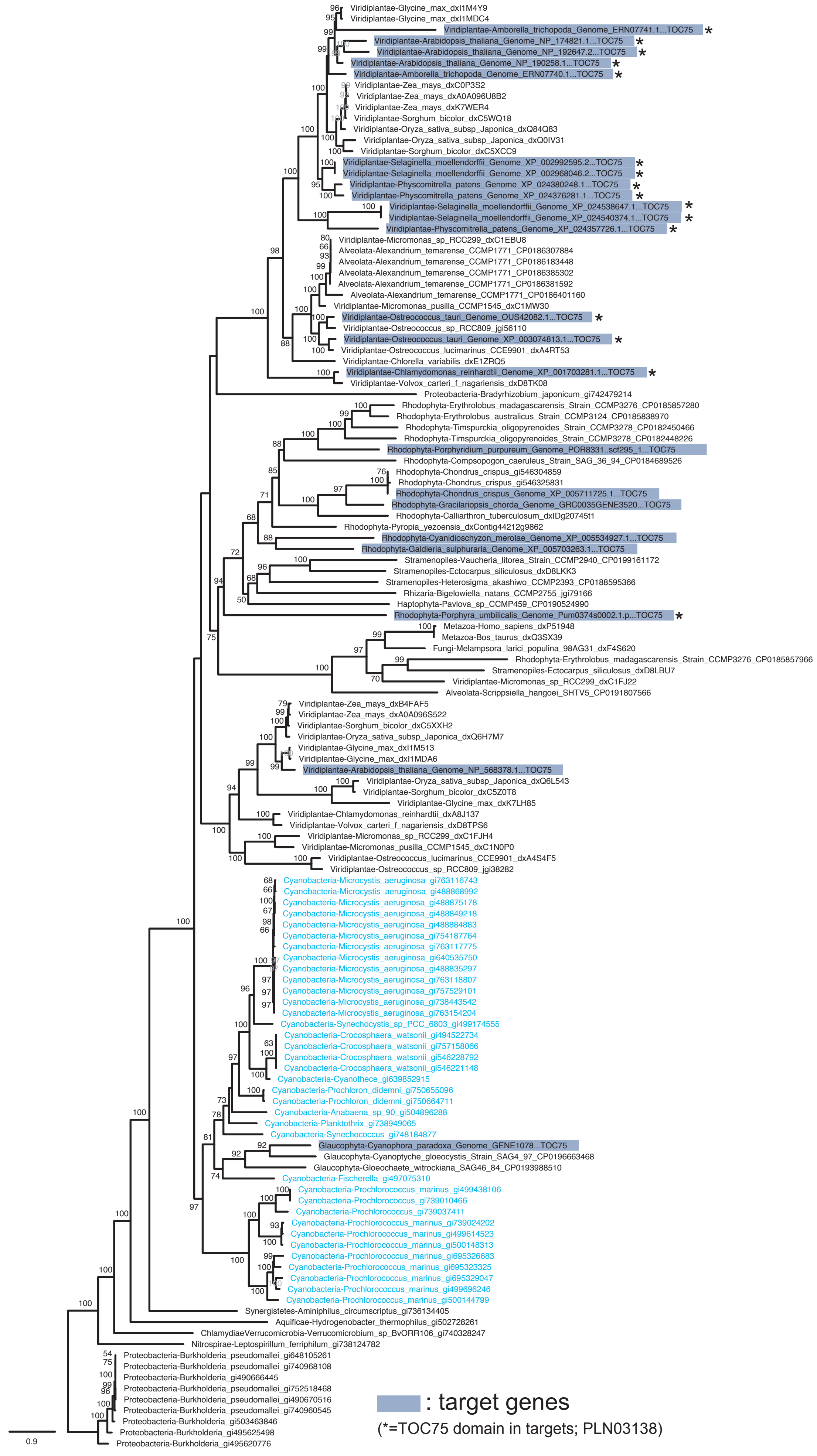

Supplementary Figure 5. ML tree of aligned TOC75 homologous genes. ML trees built using each aligned homologous gene sets of several TIC/TOC components indicated ancient endosymbiotic gene transfers (Blastp *e*-value cutoff=1.e-05 to local RefSeq database; IQ-tree program with 1,000 replications; ≥ 50 bootstrap supporting values).

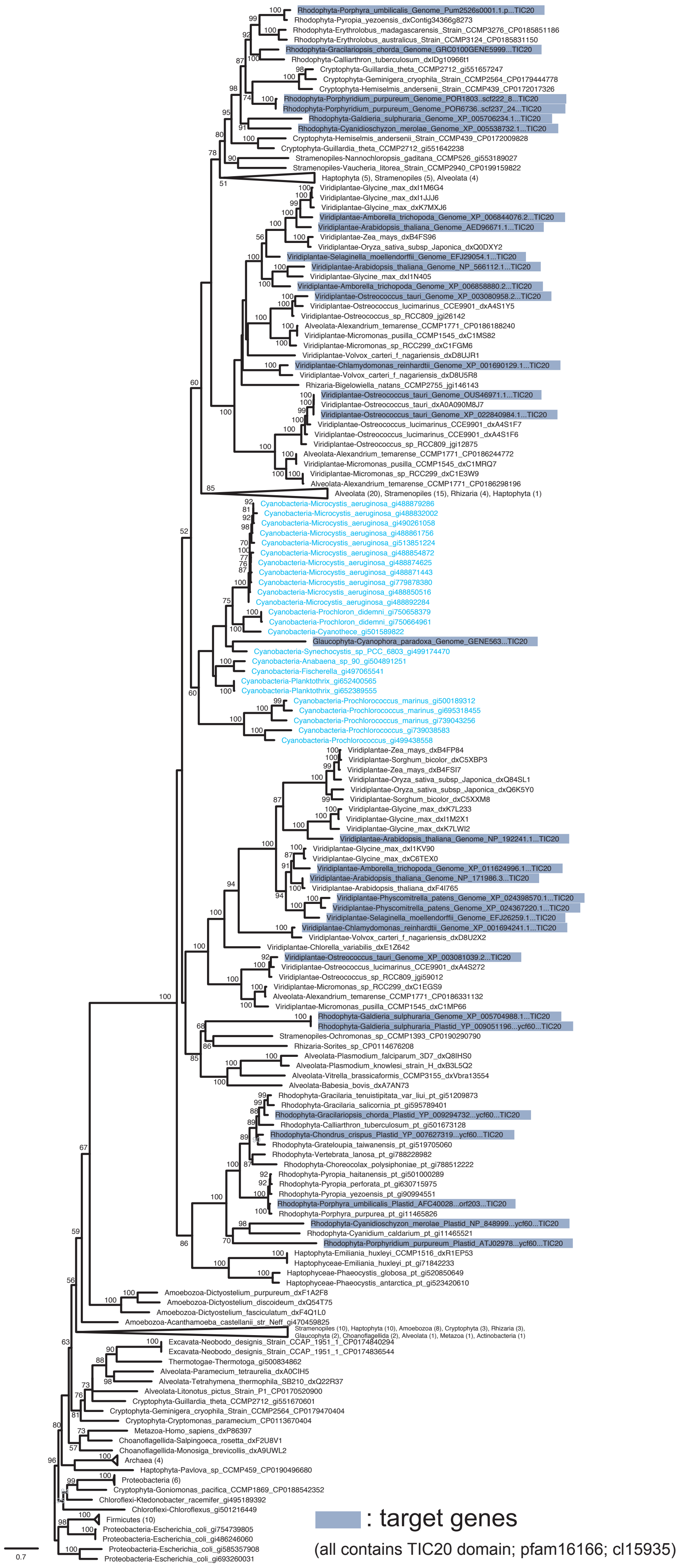

Supplementary Figure 6. ML tree of aligned TIC20 homologous genes. ML trees built using each aligned homologous gene sets of several TIC/TOC components indicated ancient endosymbiotic gene transfers (Blastp *e*-value cutoff=1.e-05 to local RefSeq database; IQ-tree program with 1,000 replications; ≥ 50 bootstrap supporting values).

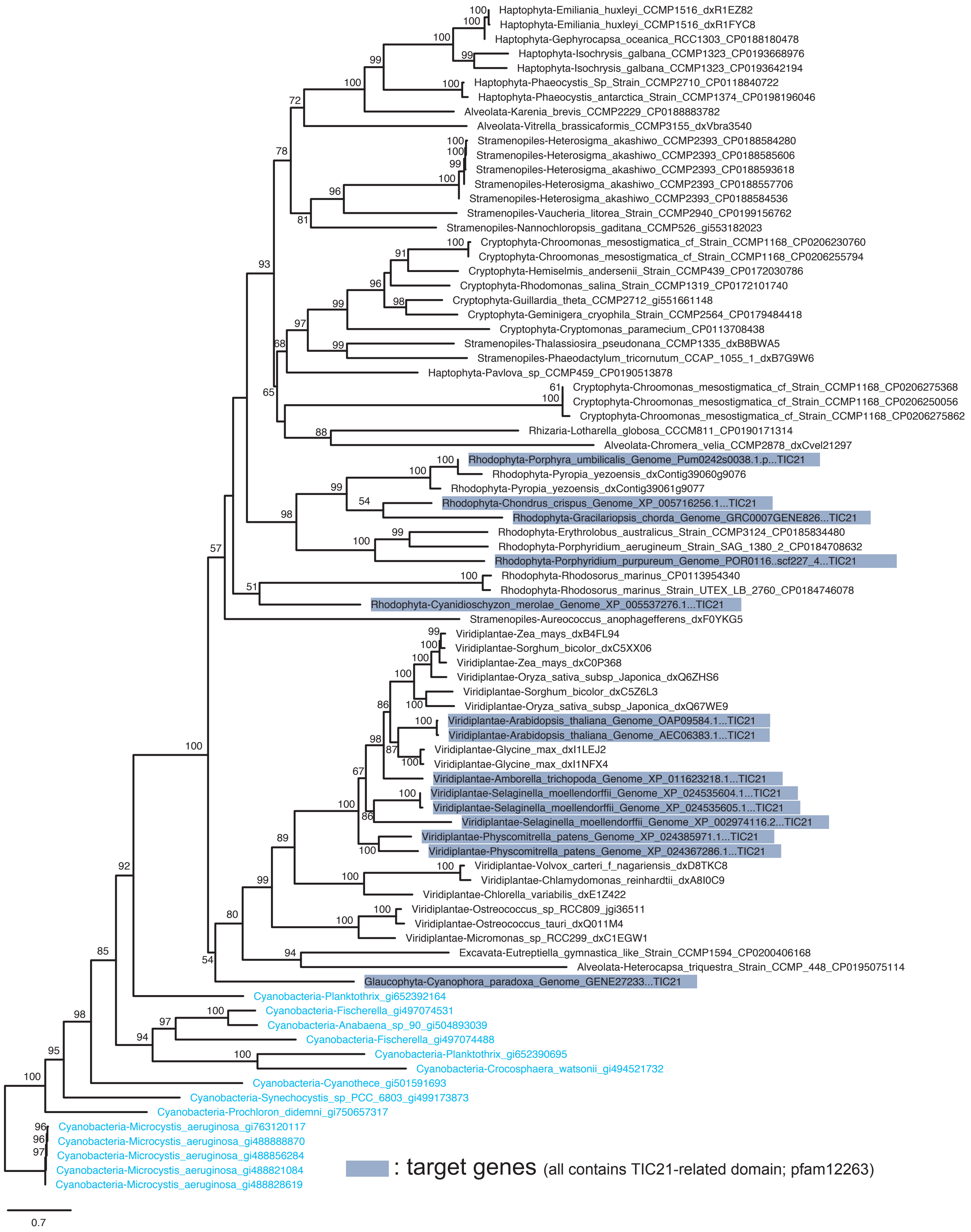

Supplementary Figure 7. ML tree of aligned TIC21 homologous genes. ML trees built using each aligned homologous gene sets of several TIC/TOC components indicated ancient endosymbiotic gene transfers (Blastp *e*-value cutoff=1.e-05 to local RefSeq database; IQ-tree program with 1,000 replications; ≥ 50 bootstrap supporting values).

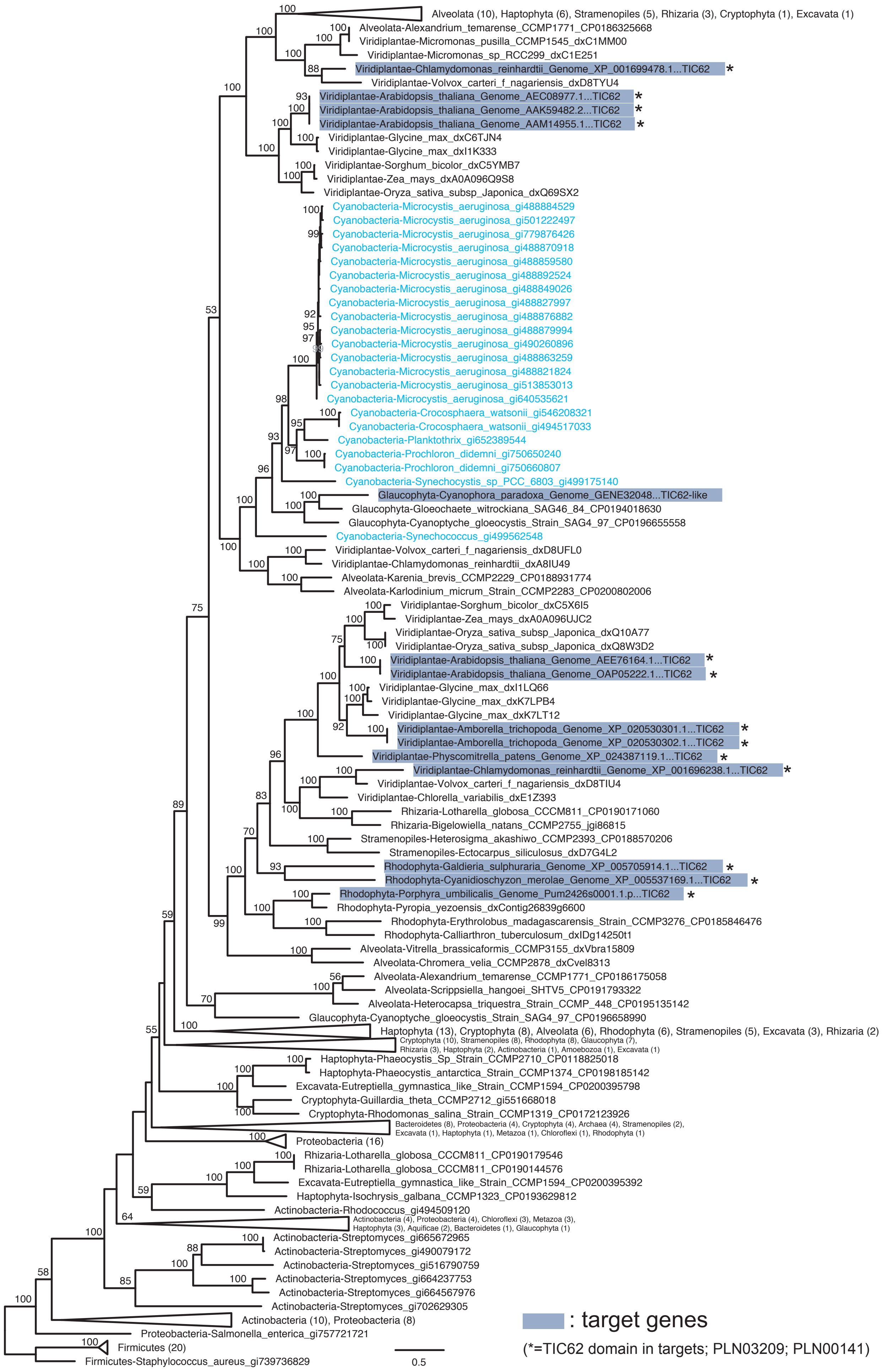

Supplementary Figure 8. ML tree of aligned TIC62 homologous genes. ML trees built using each aligned homologous gene sets of several TIC/TOC components indicated ancient endosymbiotic gene transfers (Blastp *e*-value cutoff=1.e-05 to local RefSeq database; IQ-tree program with 1,000 replications; ≥ 50 bootstrap supporting values).

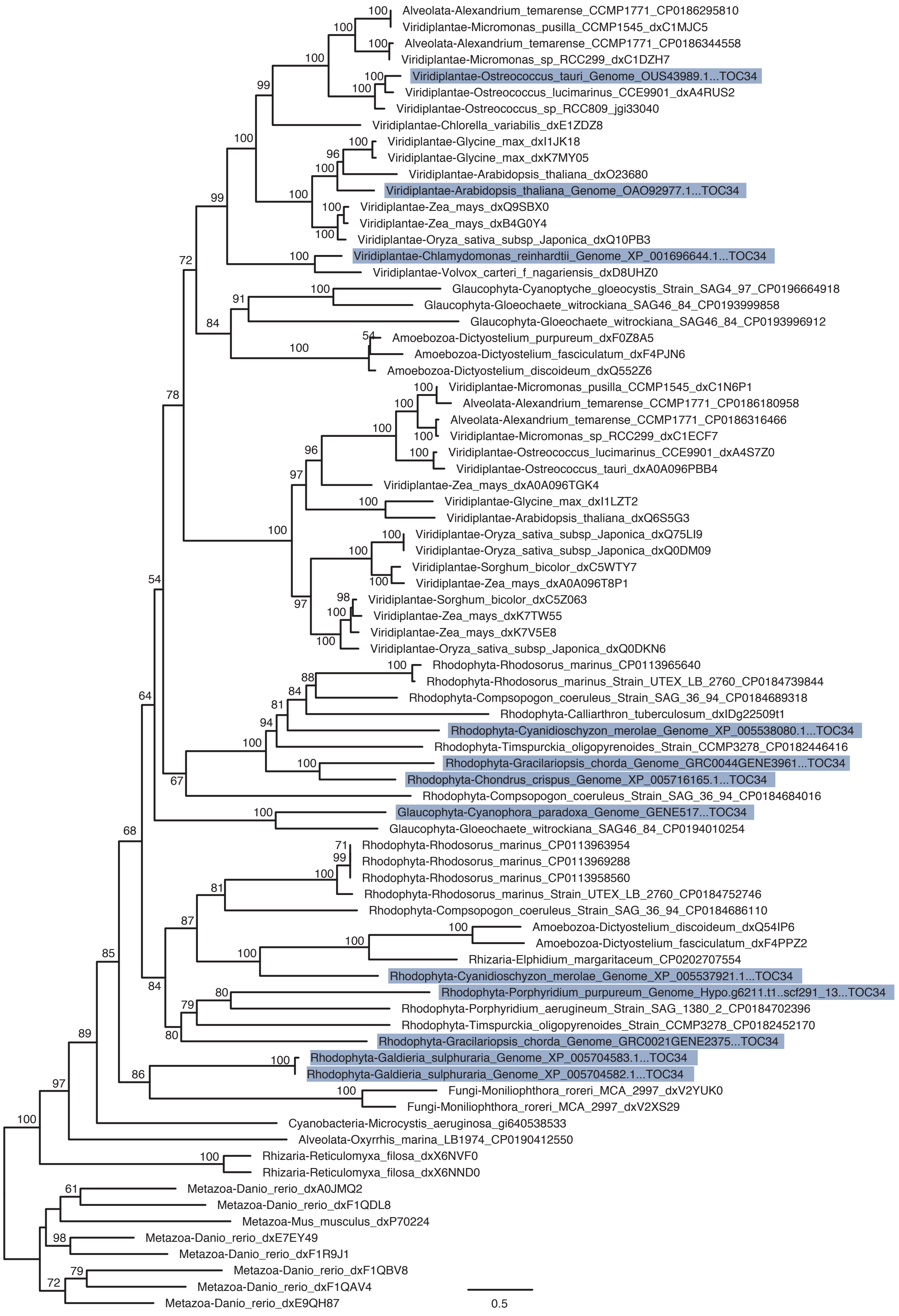

Supplementary Figure 9. ML tree of aligned TOC34 homologous genes. ML trees built using each aligned homologous gene sets of several TIC/TOC components indicated ancient endosymbiotic gene transfers (Blastp *e*-value cutoff=1.e-05 to local RefSeq database; IQ-tree program with 1,000 replications; ≥ 50 bootstrap supporting values).

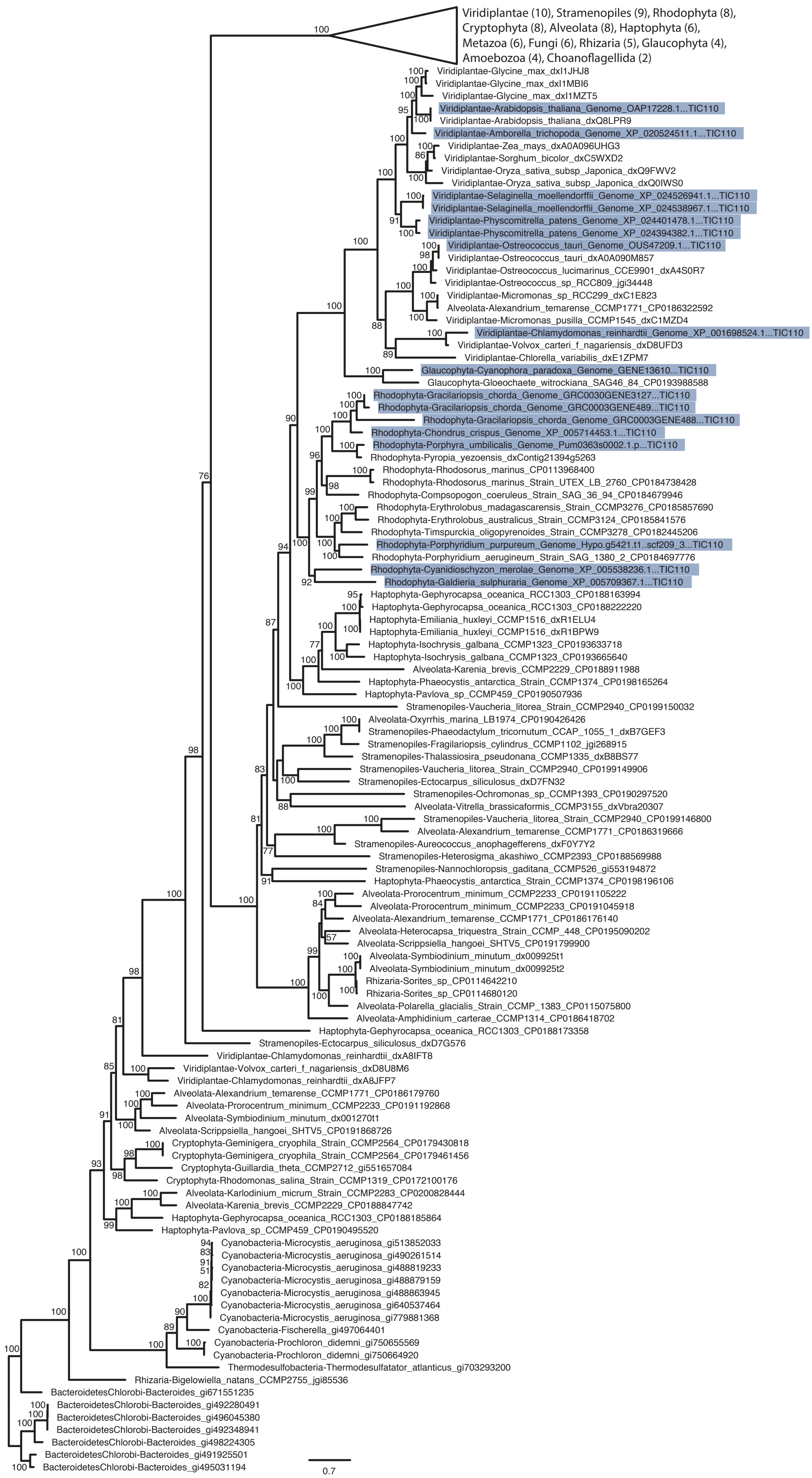

Supplementary Figure 10. ML tree of aligned TIC110 homologous genes. ML trees built using each aligned homologous gene sets of several TIC/TOC components indicated ancient endosymbiotic gene transfers (Blastp *e*-value cutoff=1.e-05 to local RefSeq database; IQ-tree program with 1,000 replications; ≥ 50 bootstrap supporting values).

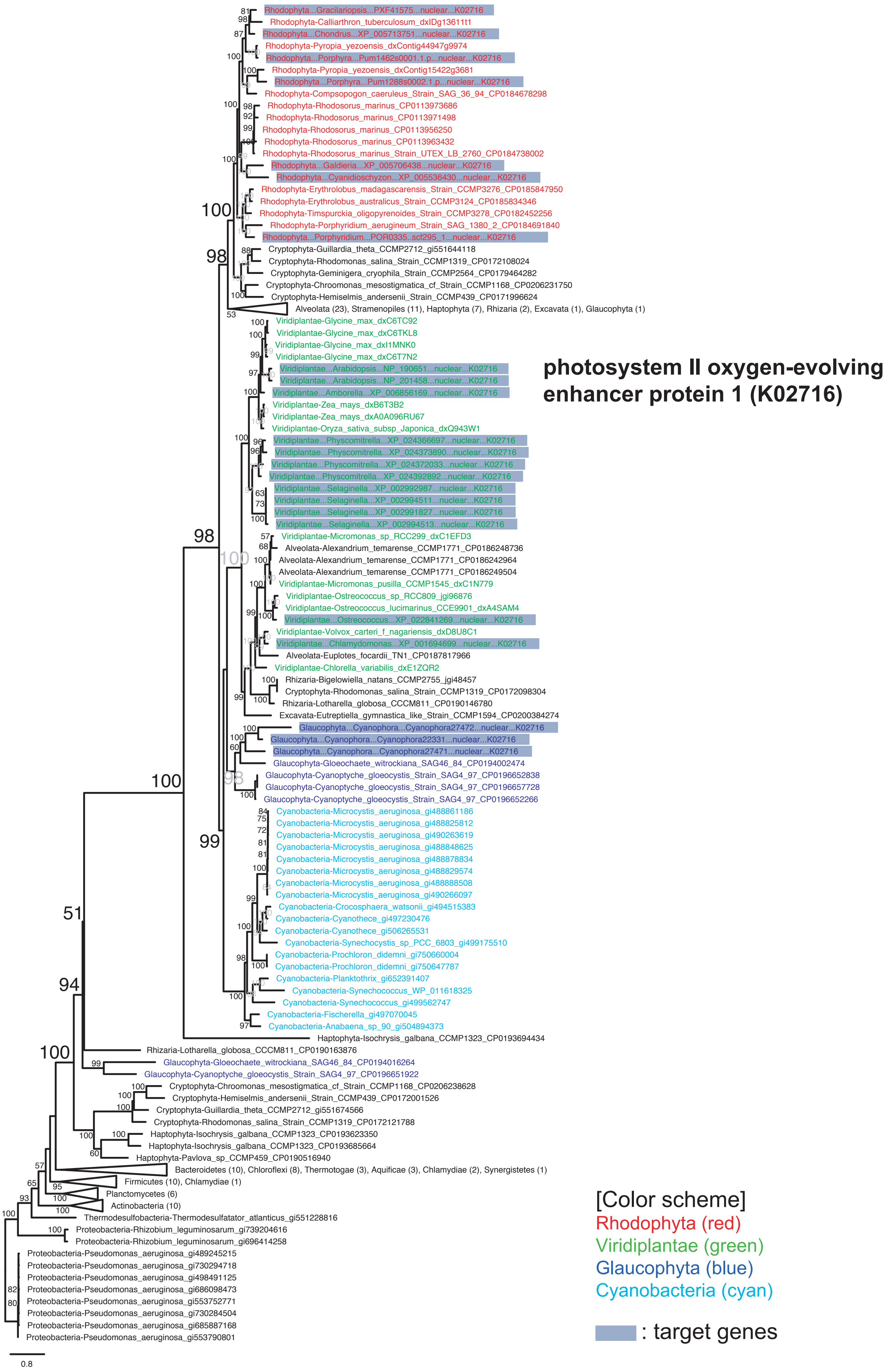

Supplementary Figure 11. ML tree of aligned photosystem II oxygen-evolving enhancer protein 1 homologous genes. ML trees built using each aligned homologous gene sets of photosynthetic function proteins indicated as ancient endosymbiotic gene transfers (Blastp *e*-value cutoff=1.e-05 to local RefSeq database; IQ-tree program with 1,000 replications; ≥ 50 bootstrap supporting values).

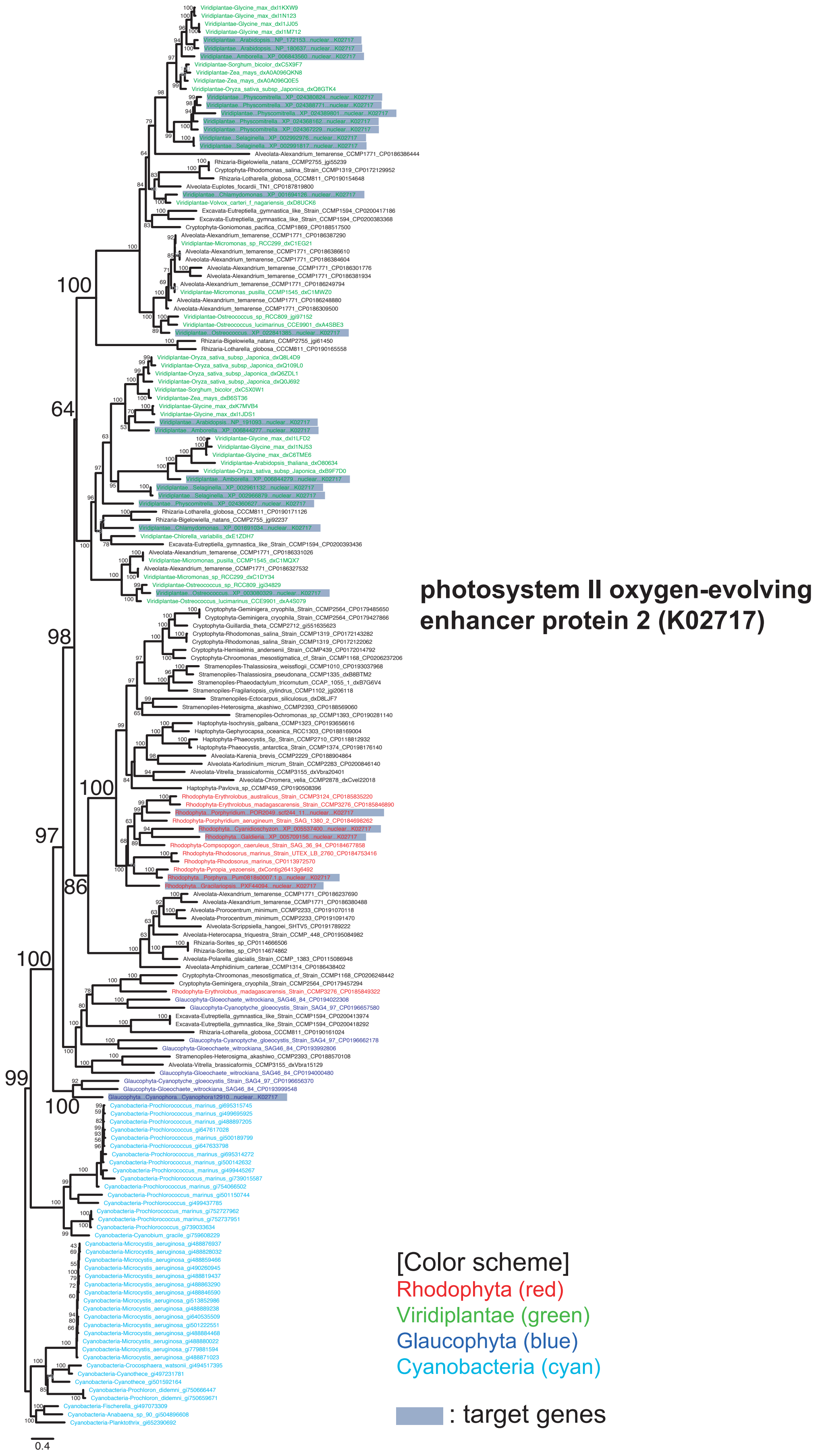

Supplementary Figure 12. ML tree of aligned photosystem II oxygen-evolving enhancer protein 2 homologous genes. ML trees built using each aligned homologous gene sets of photosynthetic function proteins indicated as ancient endosymbiotic gene transfers (Blastp *e*-value cutoff=1.e-05 to local RefSeq database; IQ-tree program with 1,000 replications; ≥ 50 bootstrap supporting values).

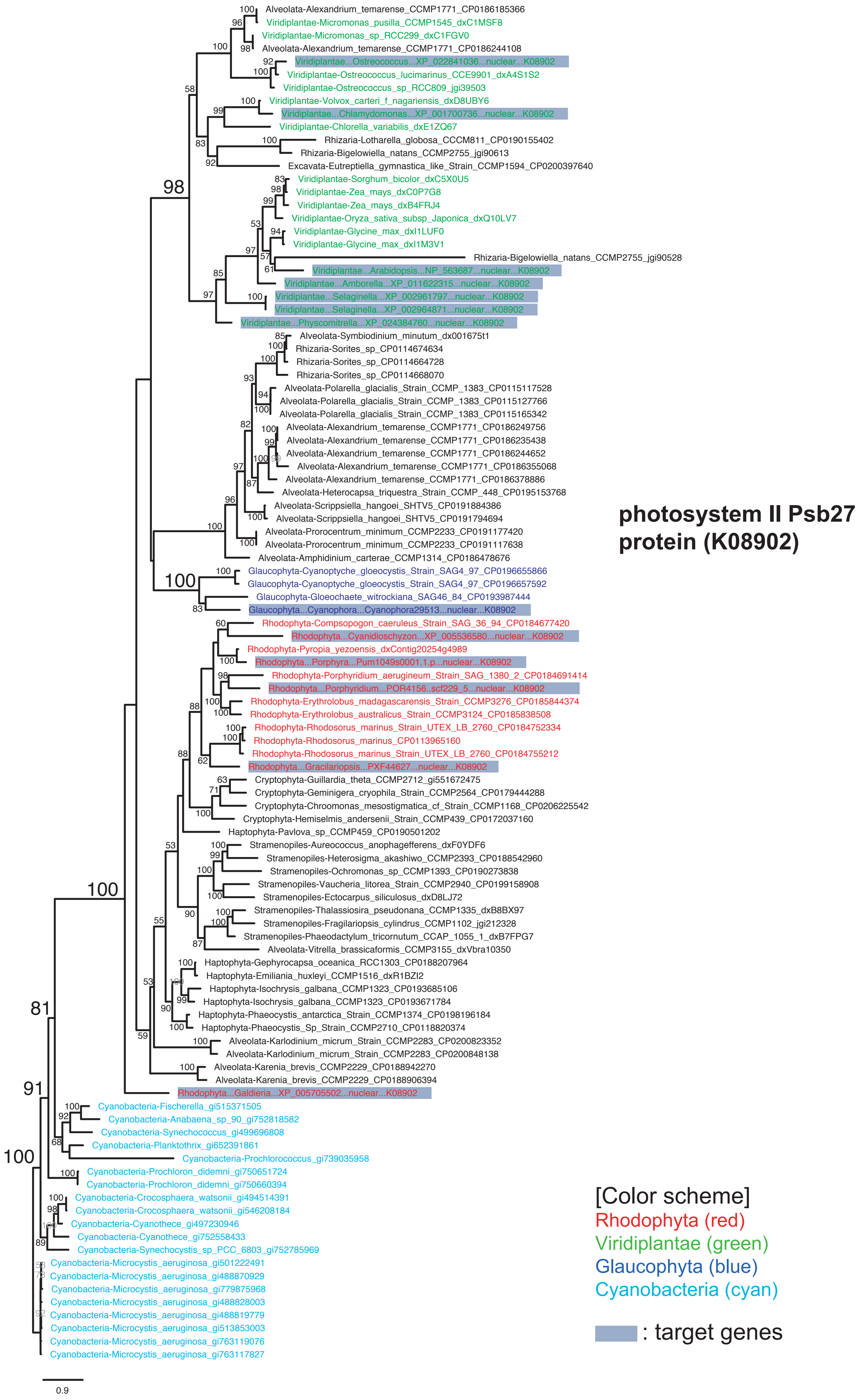

Supplementary Figure 13. ML tree of aligned photosystem II psb27 protein homologous genes. ML trees built using each aligned homologous gene sets of photosynthetic function proteins indicated as ancient endosymbiotic gene transfers (Blastp *e*-value cutoff=1.e-05 to local RefSeq database; IQ-tree program with 1,000 replications; ≥ 50 bootstrap supporting values).

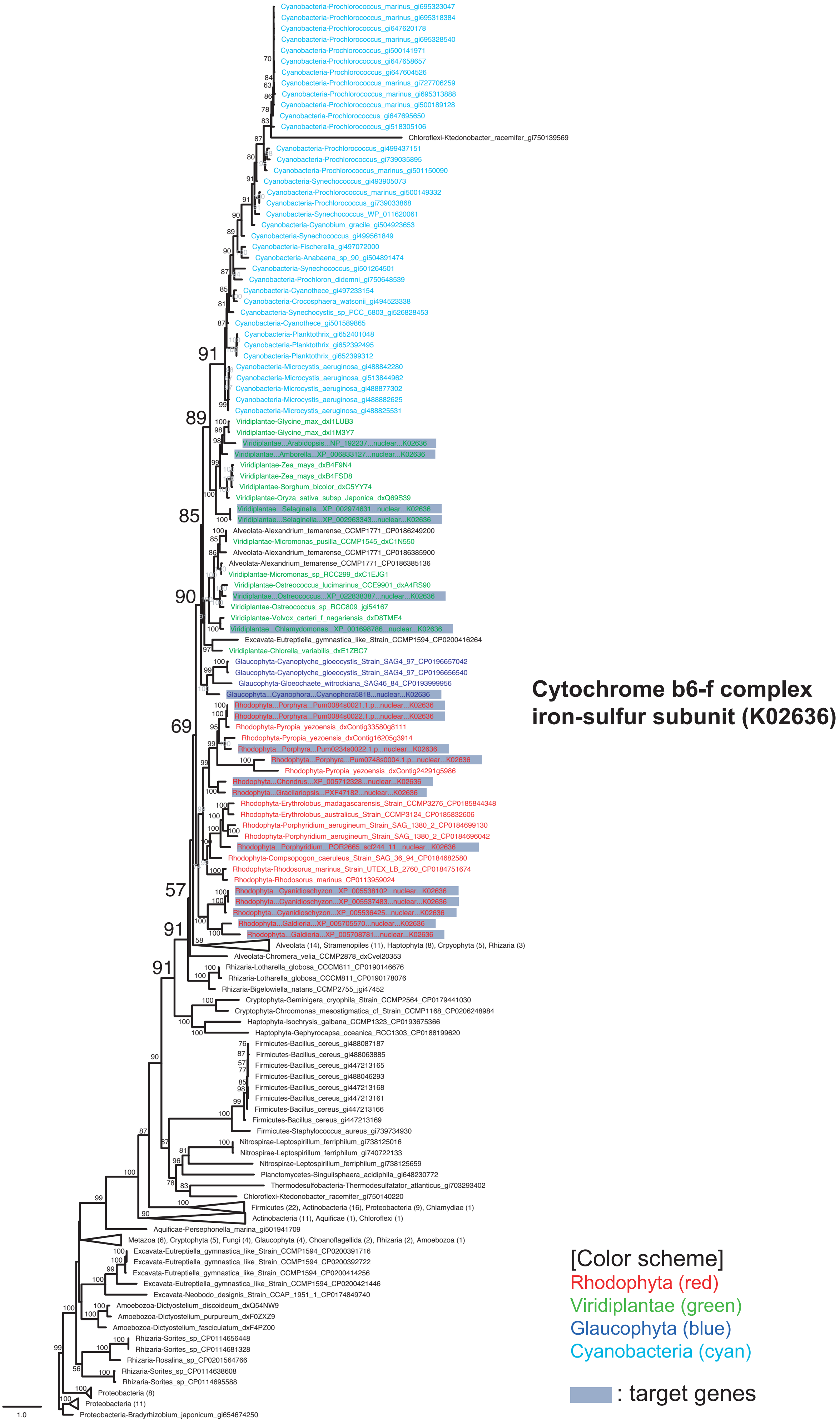

Supplementary Figure 14. ML tree of aligned cytochrome b6-f complex iron-sulfur subunit homologous genes. ML trees built using each aligned homologous gene sets of photosynthetic function proteins indicated as ancient endosymbiotic gene transfers (Blastp *e*-value cutoff=1.e-05 to local RefSeq database; IQ-tree program with 1,000 replications; ≥ 50 bootstrap supporting values).

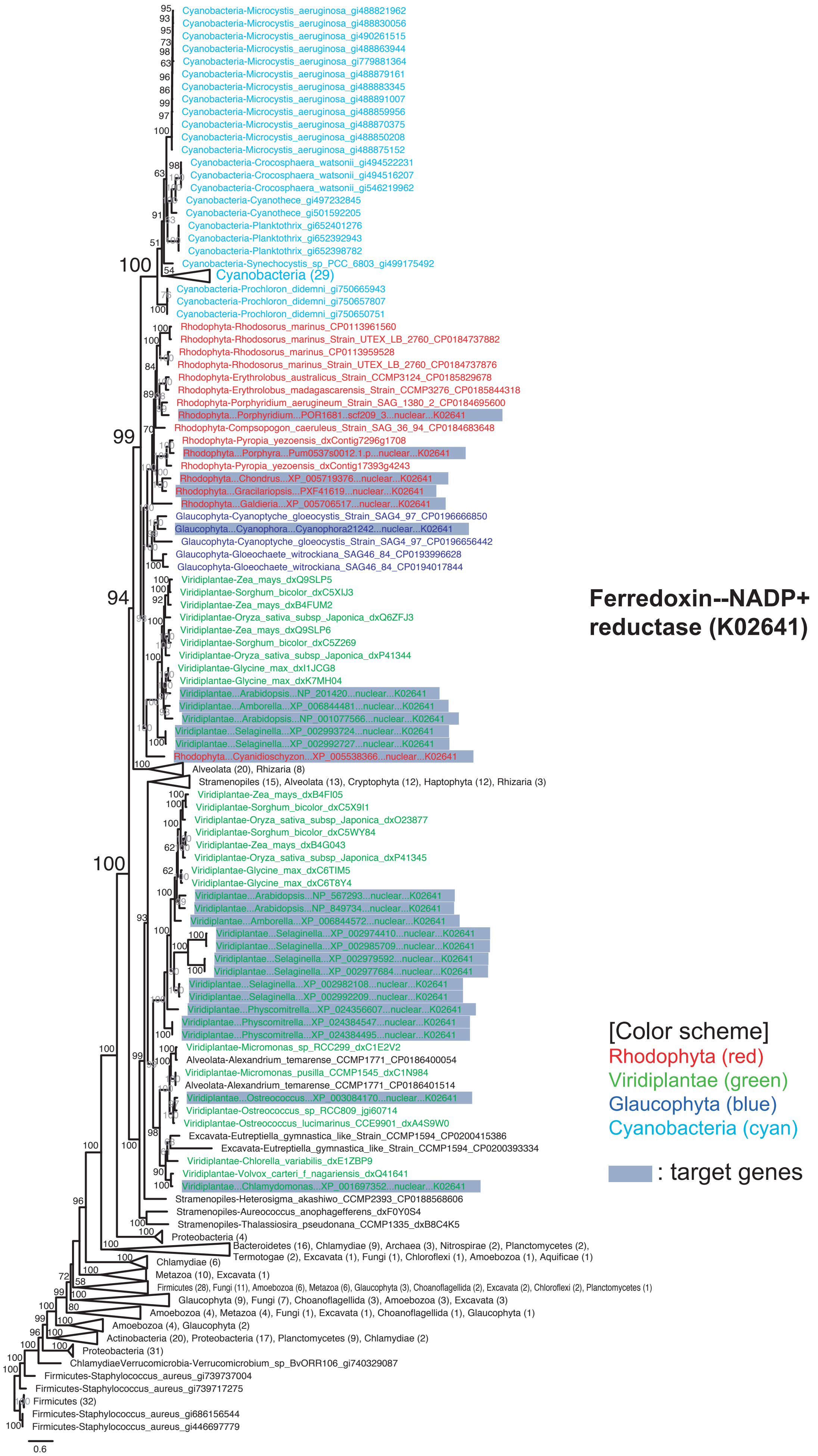

Supplementary Figure 15. ML tree of aligned ferredoxin-NADP+ reductase homologous genes. ML trees built using each aligned homologous gene sets of photosynthetic function proteins indicated as ancient endosymbiotic gene transfers (Blastp *e*-value cutoff=1.e-05 to local RefSeq database; IQ-tree program with 1,000 replications; ≥ 50 bootstrap supporting values).

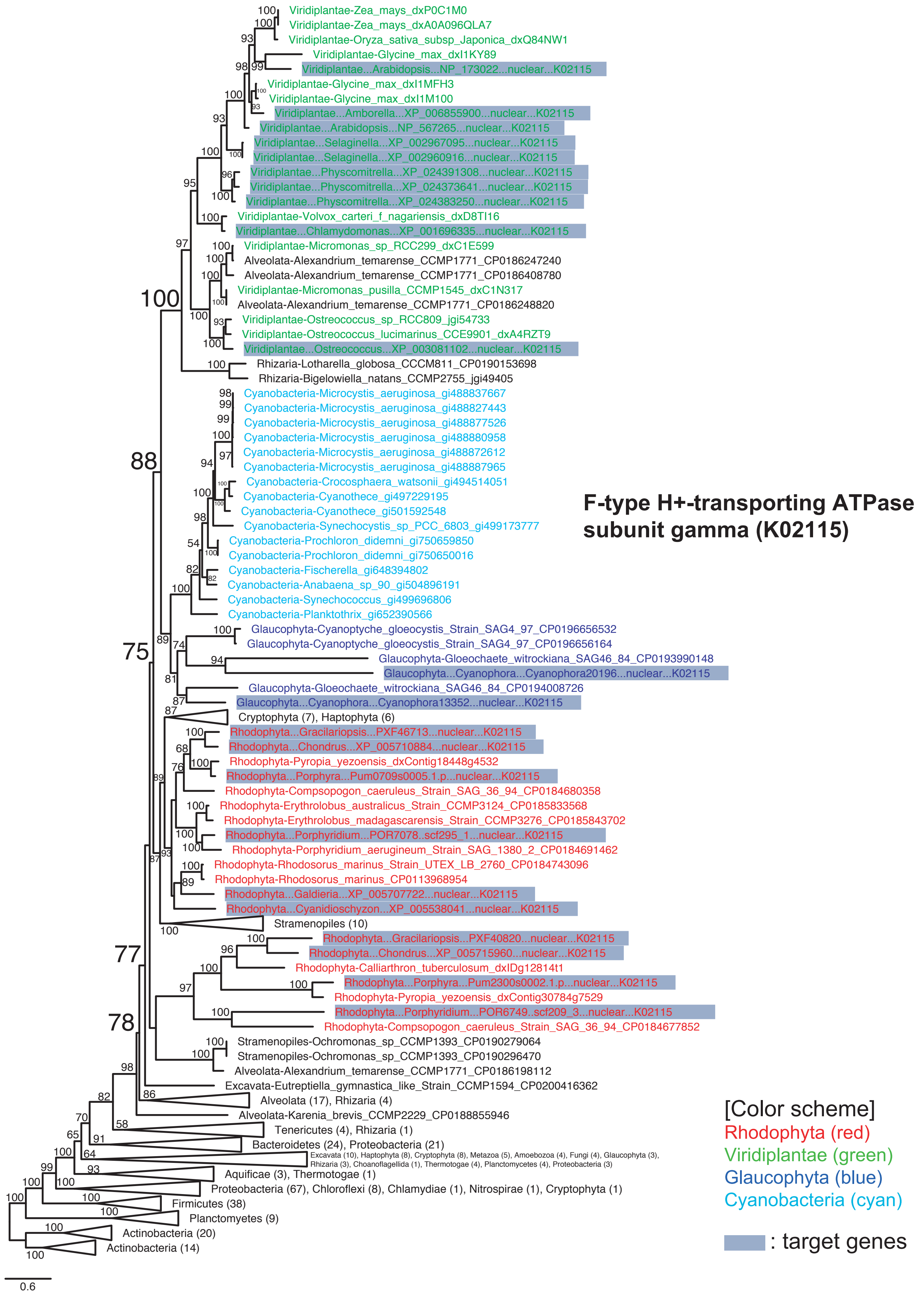

Supplementary Figure 16. ML tree of aligned F-type H+-transporting ATPase subunit gamma homologous genes. ML trees built using each aligned homologous gene sets of photosynthetic function proteins indicated as ancient endosymbiotic gene transfers (Blastp *e*-value cutoff=1.e-05 to local RefSeq database; IQ-tree program with 1,000 replications; ≥ 50 bootstrap supporting values).

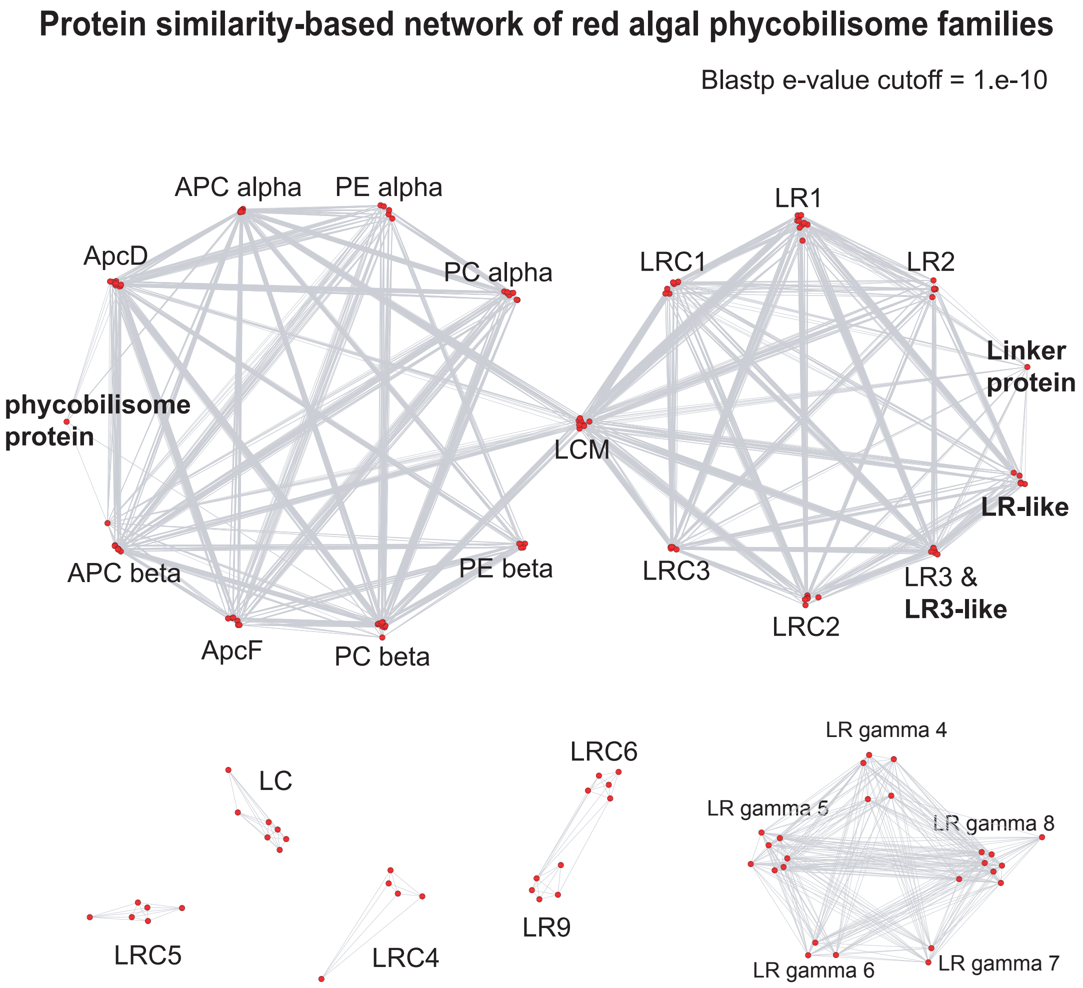

Supplementary Figure 17. Protein similarity-based network of red algal phycobilisome families. Blastp *e*-value cutoff is 1.e-10. It was drawn using Cytoscape.

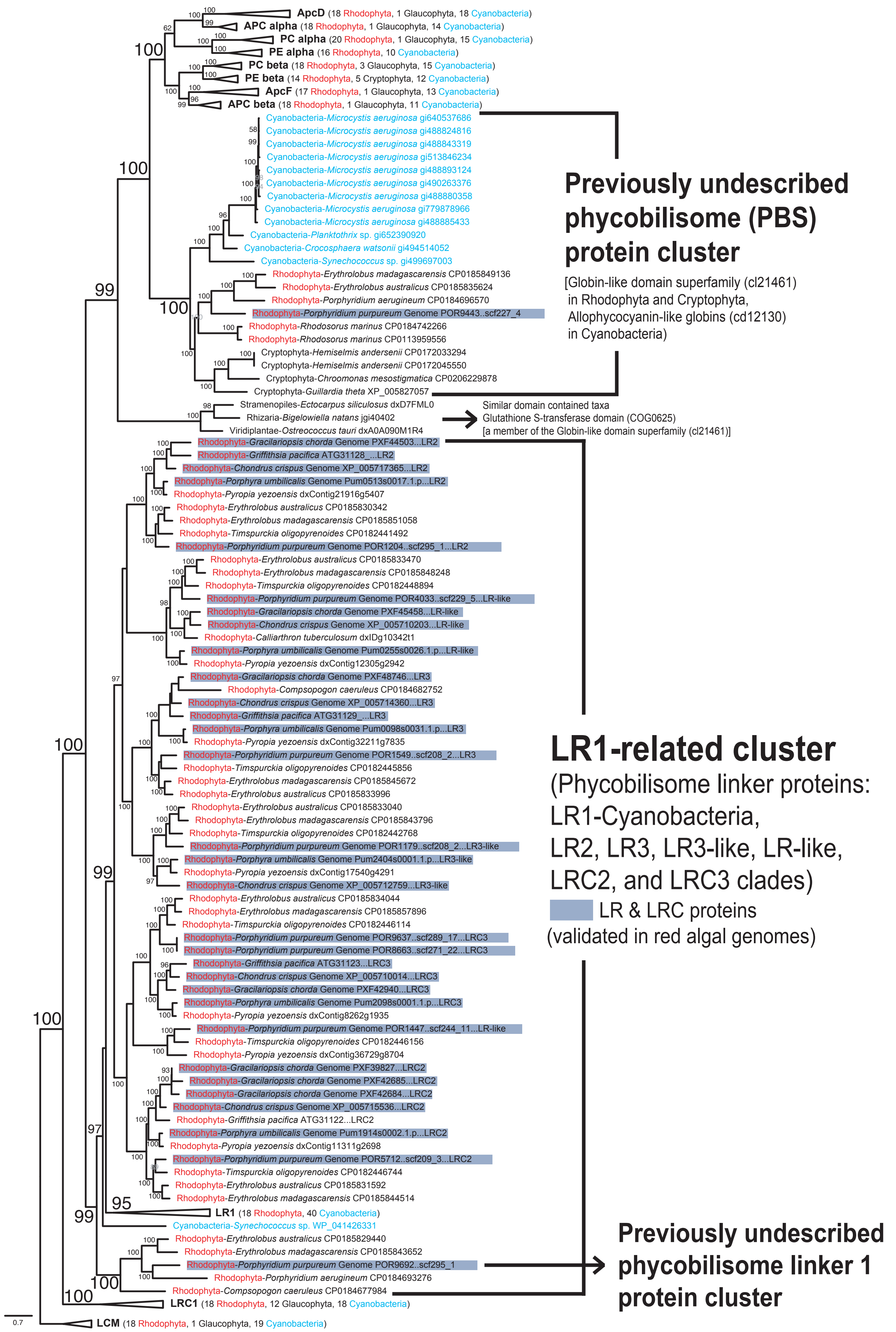

Supplementary Figure 18. The origins of all phycobilisome antenna pigments and their homologous phycobilisome linker proteins. ML tree built using aligned phycobilisome antenna and linker families including their homologous genes (Blastp *e*-value cutoff=1.e-05 to local RefSeq database; IQ-tree program with 1,000 replications; ≥ 50 bootstrap supporting values).

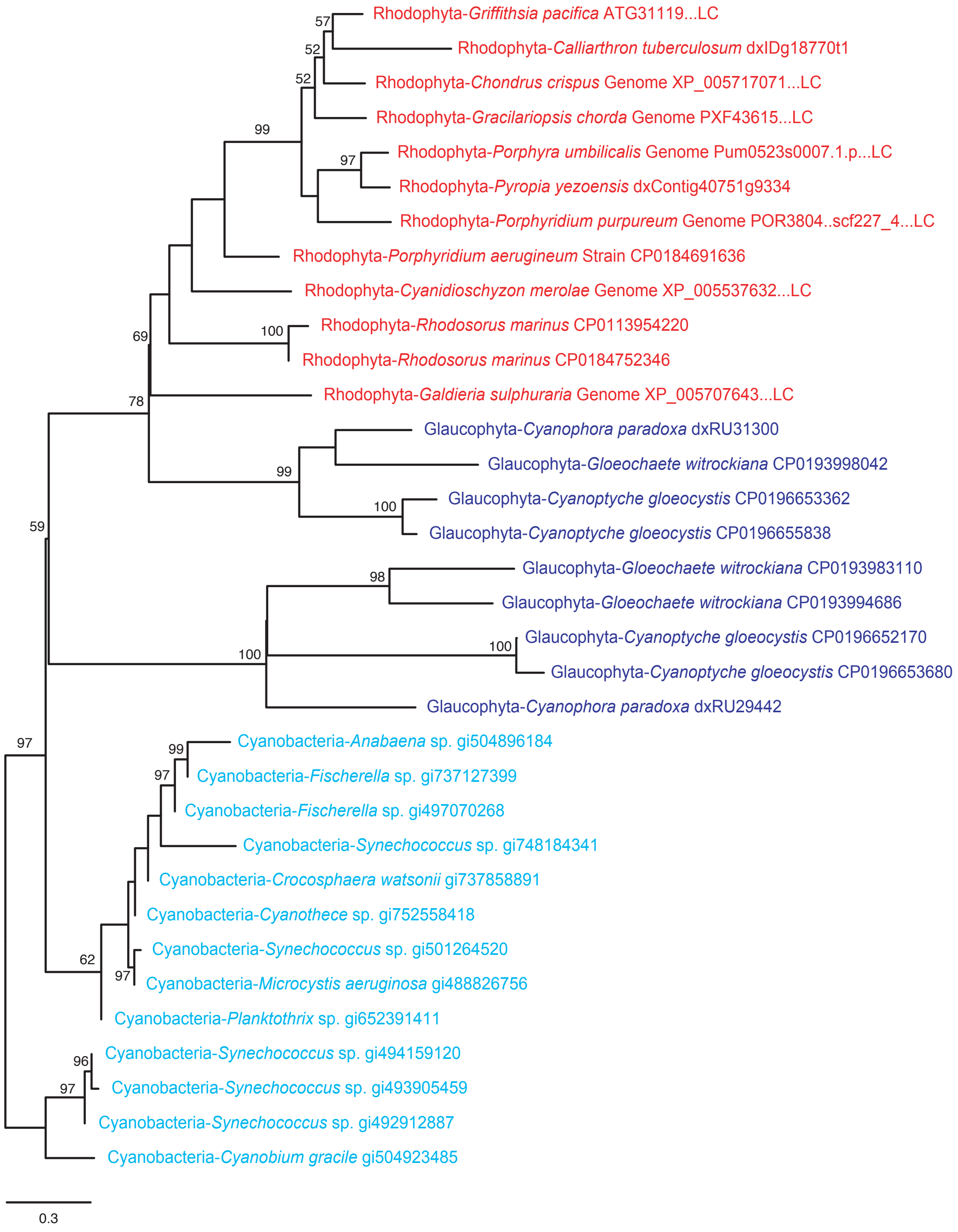

Supplementary Figure 19. ML tree built using aligned LC, APC trimers core linker protein family, homologous genes. IQ-tree program was done with 1,000 replications (Blastp *e*-value cutoff=1.e-05 to local RefSeq database, ≥ 50 bootstrap supporting values).

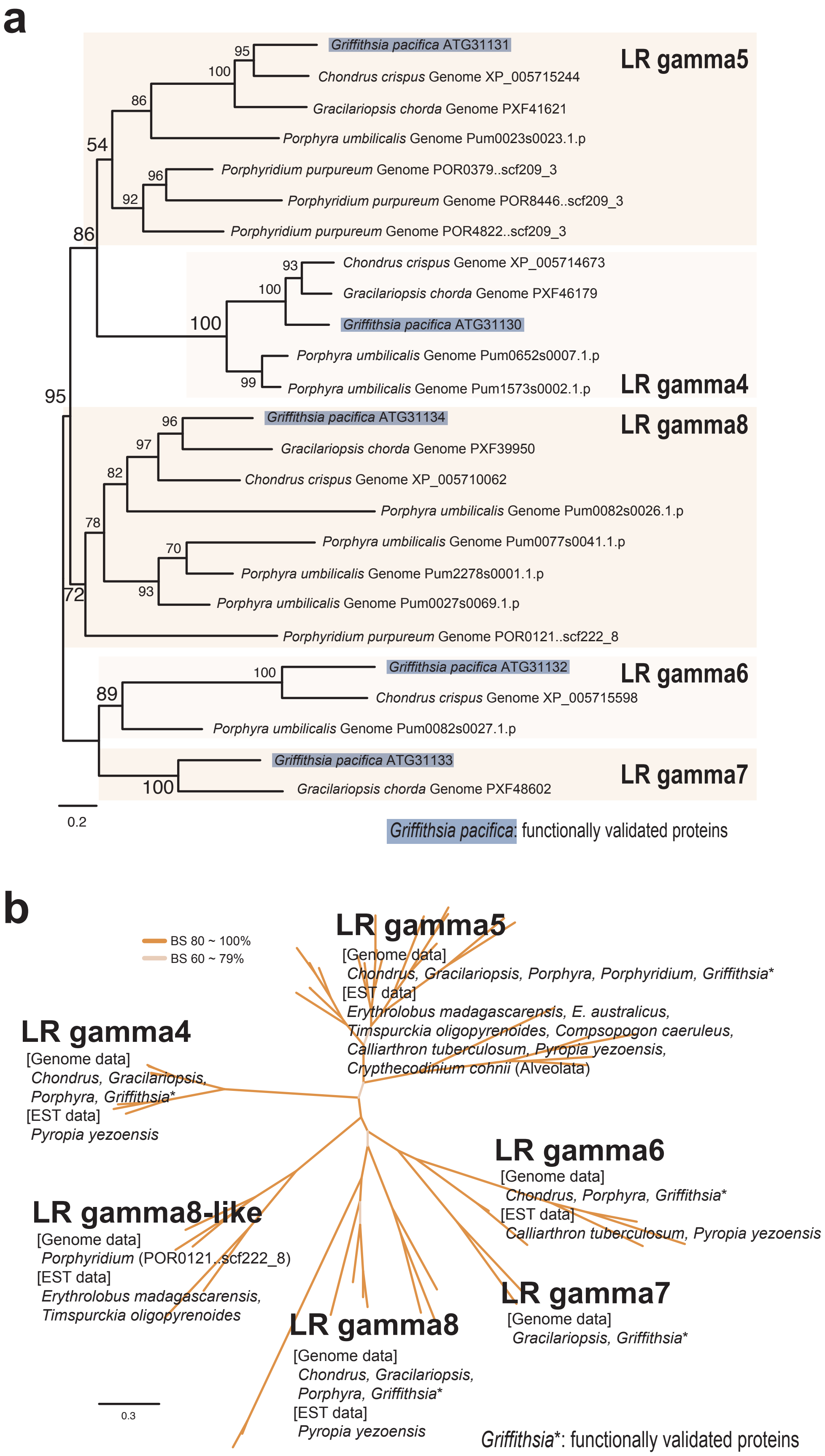

Supplementary Figure 20. ML trees built using each aligned homologous genes of LR gamma 4 ~ 8, phycobilisome rod linker gamma, families based on rooted and un-rooted ML trees. a ML tree of aligned LR gamma families in red algal genomes. **b** ML tree of aligned LR gamma homologous genes. IQ-tree program was done with 1,000 replications (Blastp *e*-value cutoff=1.e-05 to local RefSeq or red algal genome database).

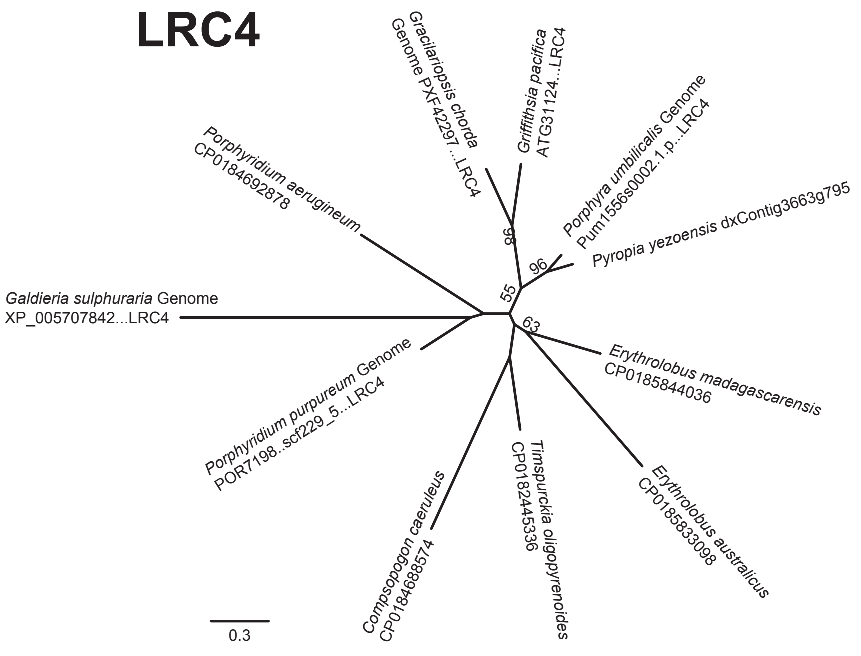

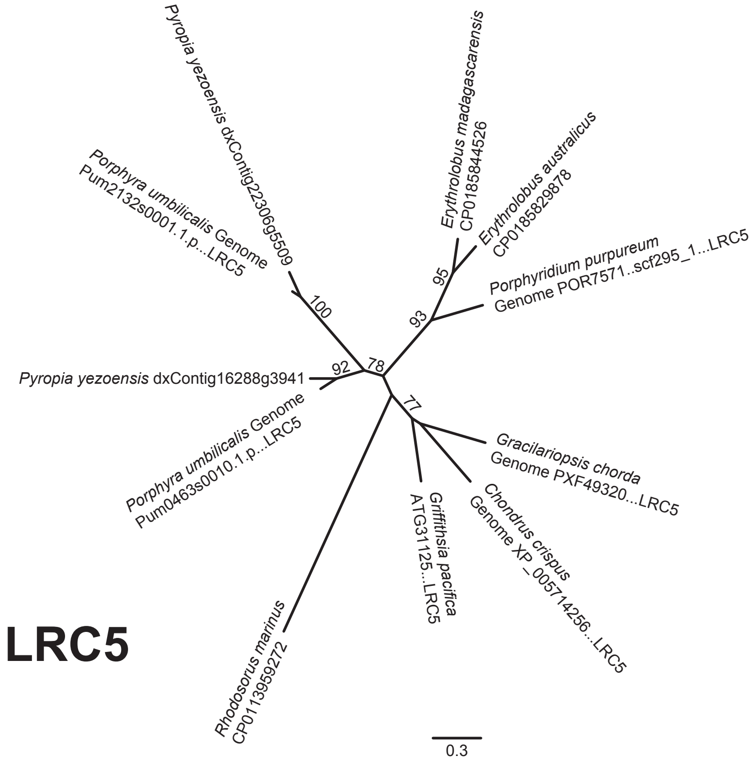

Supplementary Figure 21. Un-rooted ML trees built using each aligned phycobilisome rod-core linker, LRC4 and LRC5, homologous genes. IQ-tree program was done with 1,000 replications (Blastp *e*-value cutoff= 1.e-05 to local RefSeq database; ≥ 50 bootstrap supporting values).

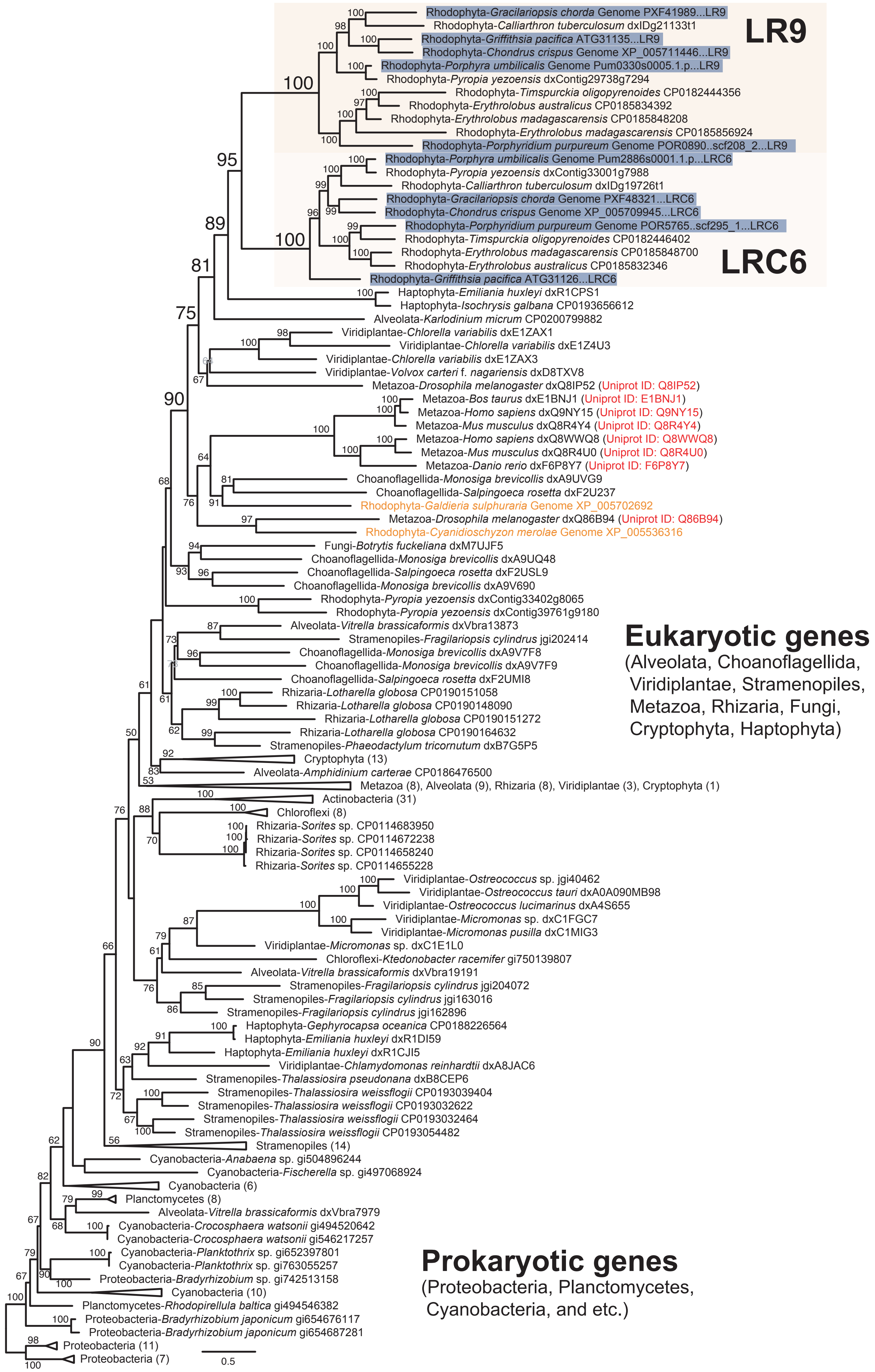

Supplementary Figure 22. ML trees built using aligned phycobilisome linker, LR9 and LRC6, homologous genes. IQ-tree program was done with 1,000 replications (Blastp *e*-value cutoff=1.e-05 to local RefSeq database; ≥ 50 bootstrap supporting values).

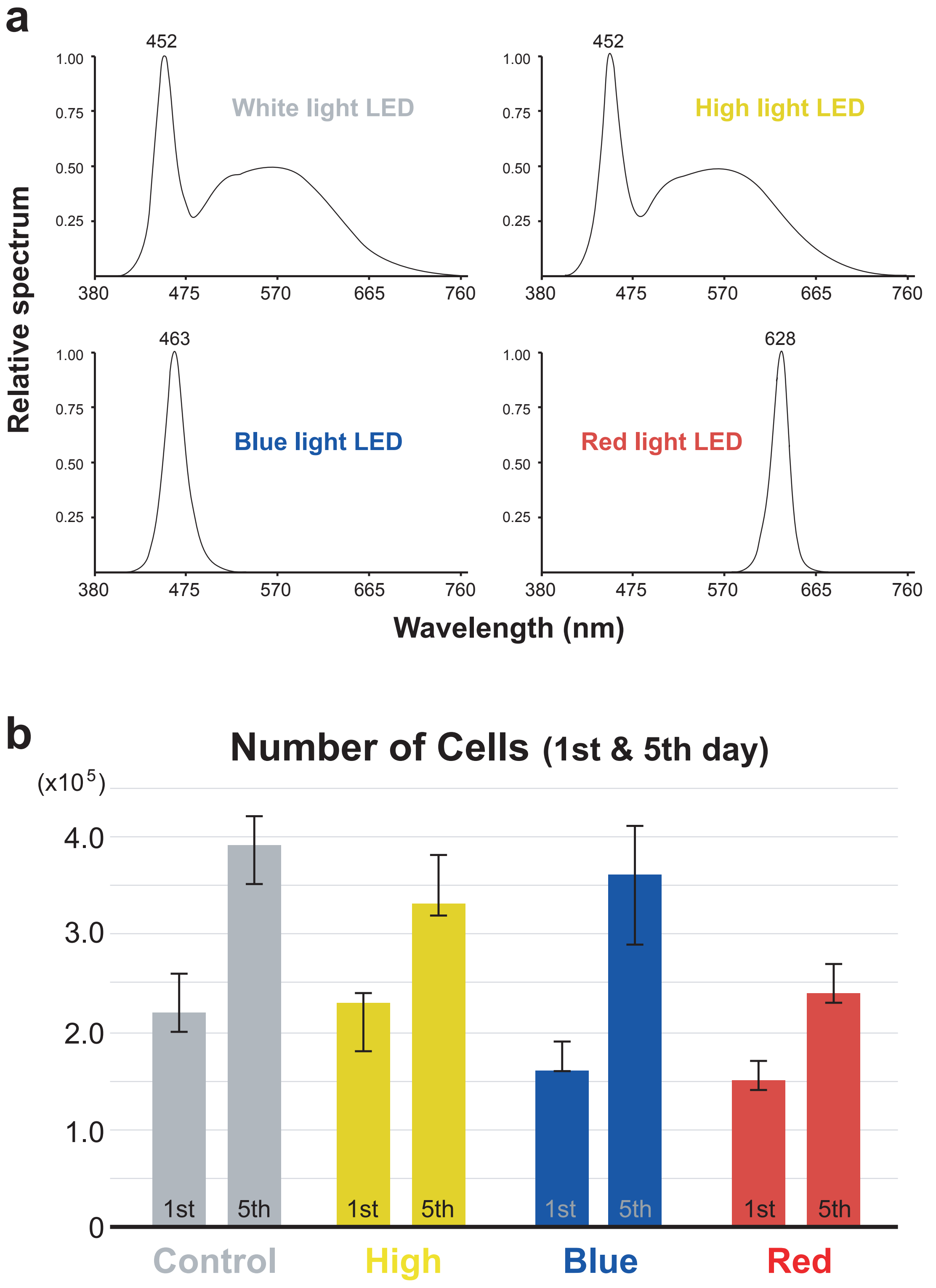

Supplementary Figure 23. Experimental conditions and cell growth. a Action spectrum of light condtions. **b** Number of cells of *Porphyridium purpureum* under different light sources (n=3 biologically independent experiments; error bars).

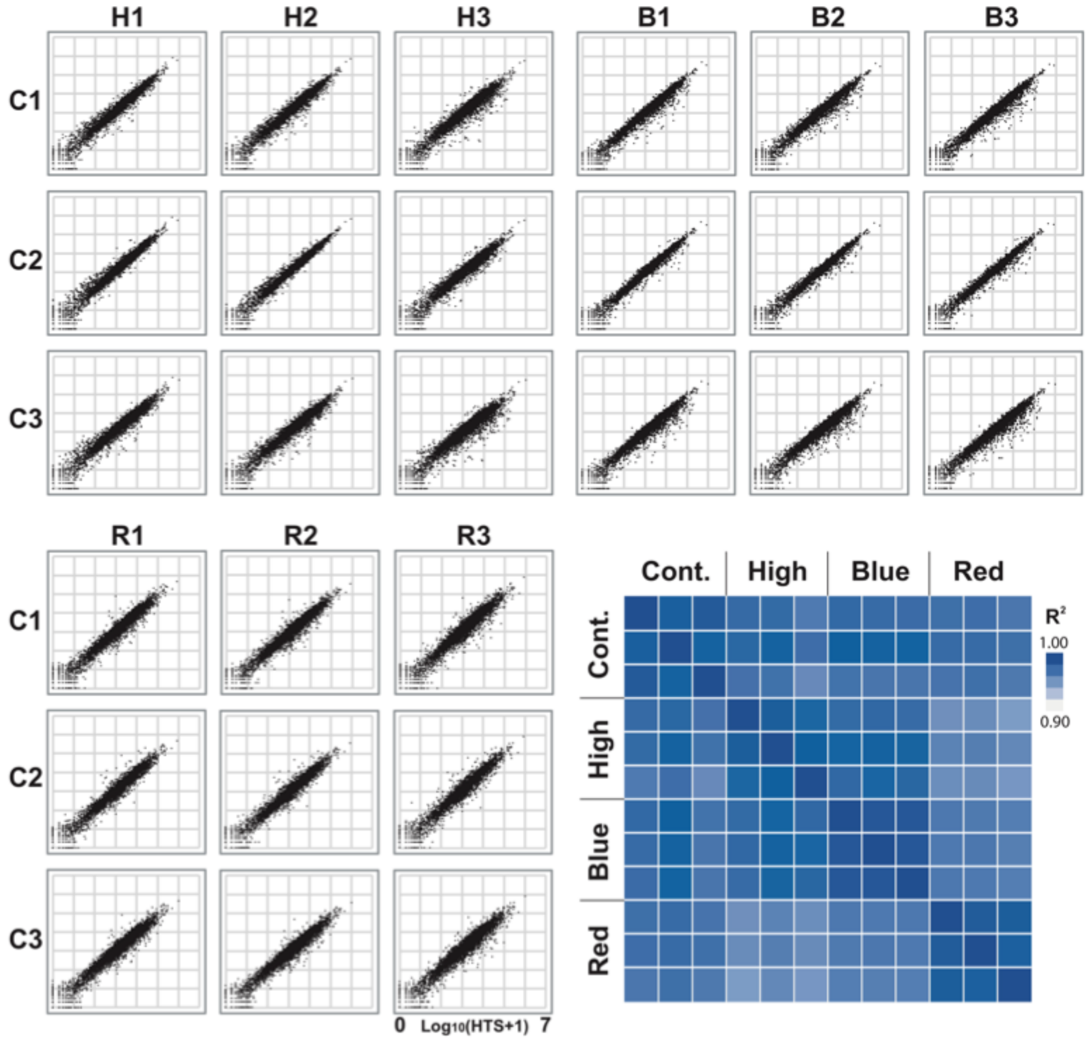

Supplementary Figure 24. Gene expression pattern analysis of *Porphyridium purpureum* under different light sources. Axis of scatter plots is Log_{10}(HTSeq-count+1). Abbreviations: Cont. and C = (white light), H = Higher light, B = Blue light, and R = Red light.

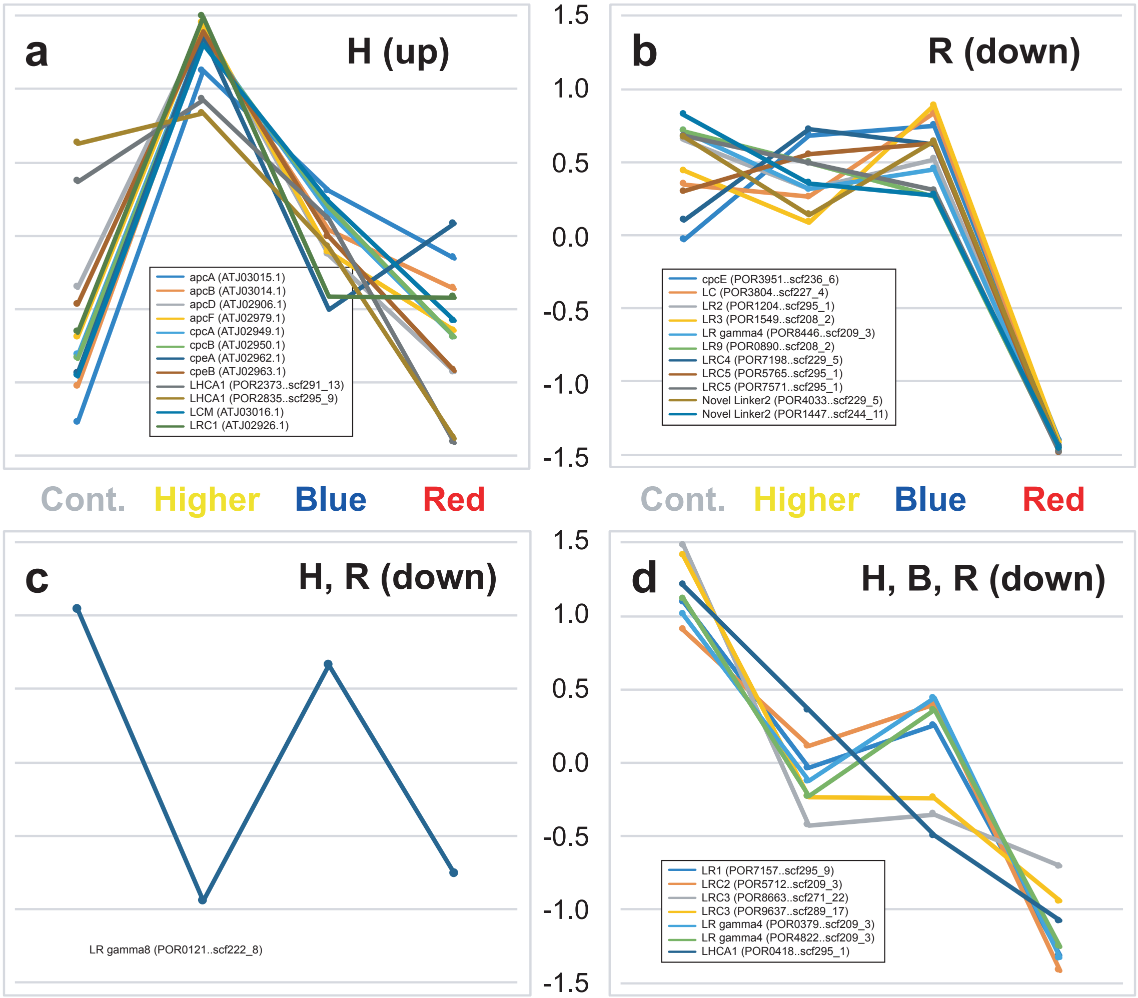

z-score normalization: ('Expression' - 'Average expression of all conditions in each gene') / 'Standard deviation of all conditions in each gene'
Filtration: maximum / minimum fold change < 1.5 folds, Cont. = Control (white light)

Supplementary Figure 25. Gene expression patterns of antenna complex genes using normalized values, z-score, with filtration with max/min fold change < 1.5 folds. a Higher light-induced groups. **b** Blue light-response groups (down-regulated only under red light). **c** Blue light response candidate (but down-regulated under high and red lights). **d** Down regulated groups under all light treatments (higher, blue, and red lights). Source data of Supplementary Figure 25 are provided as a Source Data file.

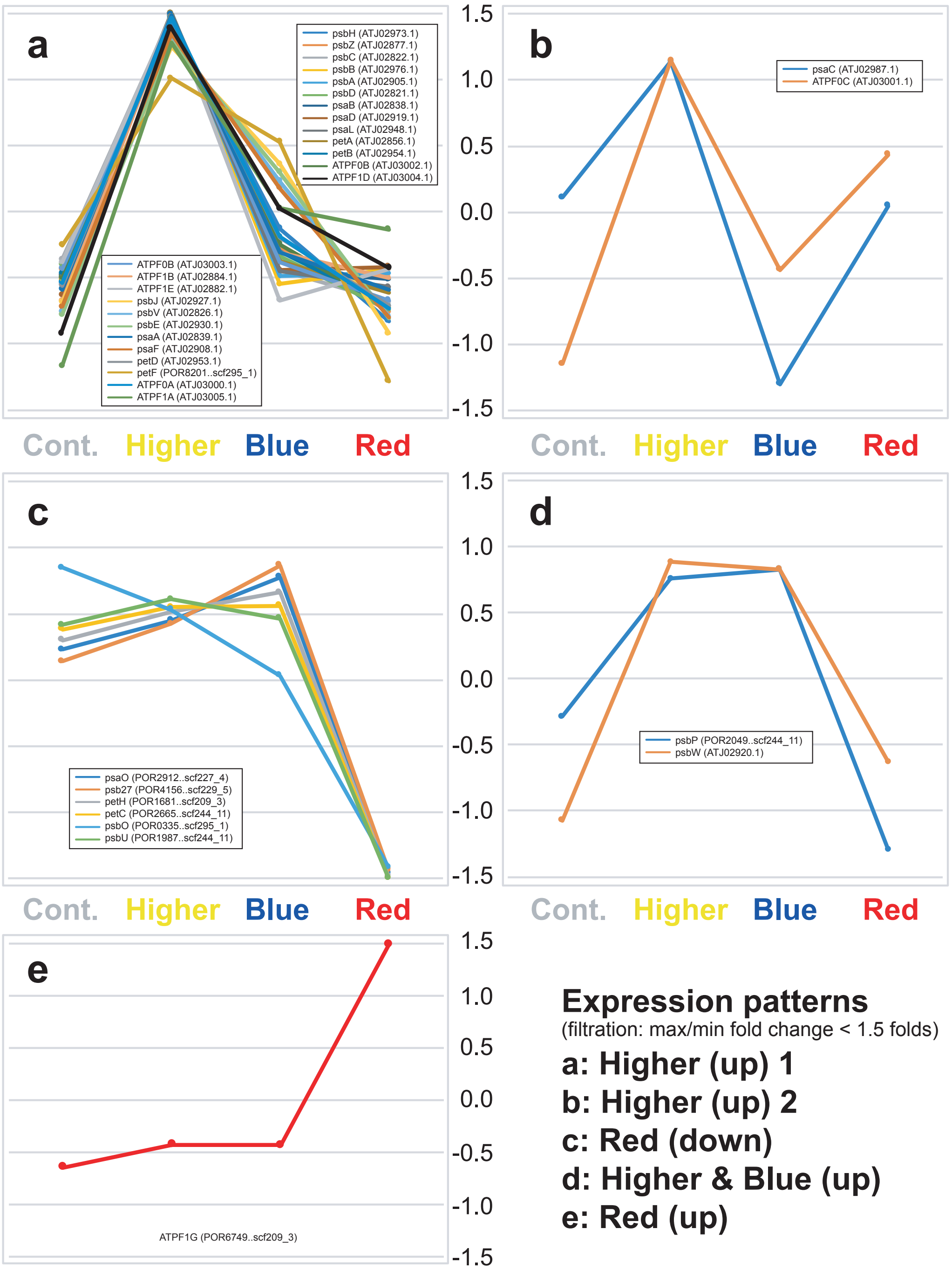

z-score normalization = ('Expression' - 'Average expression of all conditions in each gene') / 'Standard deviation of all conditions in each gene'
Filtration: maximum / minimum fold change < 1.5 folds, Cont. = Control (white light)

Supplementary Figure 26. Gene expression patterns of photosyntheic function genes using normalized values, z-score, with filtration with max/min fold change < 1.5 folds. a Higher light induced group-1. **b** Higher light-induced group-2. **c** Blue light-response groups (down regulated only under red light). **d** Blue light response candidates (but up-regulated also under higher light). **e** Up-regulated gene only in red light. Source data of Supplementary Figure 26 are provided as a Source Data file.

Supplementary References

1. Bhattacharya, D., Yoon, H. S. & Hackett, J. D. Photosynthetic eukaryotes unite: endosymbiosis connects the dots. *Bioessays* **26**, 50-60 (2004).
2. Timmis, J. N., Ayliffe, M. A., Huang, C. Y. & Martin, W. Endosymbiotic gene transfer: organelle genomes forge eukaryotic chromosomes. *Genetics* **5**, 123-135 (2004).
3. Patron, N. J. & Waller, R. F. Transit peptide diversity and divergence: a global analysis of plastid targeting signals. *Bioessays* **29**, 1048-1058 (2007).
4. Köhler, D. et al. Identification of protein N-termini in *Cyanophora paradoxa* cyanelles: transit peptide composition and sequence determinants for precursor maturation. *Front. Plant Sci.* **6**, 559 (2015).
5. Price, D. C., Steiner, J. M., Yoon, H. S., Bhattacharya, D. & Löffelhardt, W. *Glaucophyta Ch. Handbook of the protists* (Cham, Springer, 2017).
6. Gross, J. & Bhattacharya, D. Revaluating the evolution of the Toc and Tic protein translocons. *Trends Plant Sci.* **14**, 13-20 (2009).
7. Chan, C. X., Gross, J., Yoon, H. S. & Bhattacharya, D. Plastid origin and evolution: new models provide insights into old problems. *Plant Physiol.* **155**, 1552-1560 (2011).
8. Hinnah, S. C., Hill, K., Wagner, R., Schilcher, T. & Soll, J. Reconstitution of a chloroplast protein import channel. *EMBO J.* **16**, 7351-7360 (1997).
9. Kouranov, A., Chen, X., Fuks, B. & Schnell, D. J. Tic20 and Tic22 are new components of the protein import apparatus at the chloroplast inner envelope membrane. *J. Cell Biol.* **16**, 991-1002 (1998).
10. Ertel, F. et al. The evolutionarily related β-barrel polypeptide transporters from *Pisum sativum* and *Nostoc* PCC7120 contain two distinct functional domains. *J. Biol. Chem.* **280**, 28281-28289 (2005).
11. McFadden, G. I. & van Dooren, G. G. Evolution: red algal genome affirms a common origin of all plastids. *Curr. Biol.* **14**, R514-R516 (2004).
12. Löffelhardt, W. The single primary endosymbiotic event *Ch. Endosymbiosis* (Vienna, Springer, 2014).
13. Thornton, L. E. et al. Homologs of plant *psb*P and *psb*Q proteins are necessary for

regulation of photosystem II activity in the cyanobacterium *Synechocystis* 6803. *Plant Cell.* **16**, 2164-2175 (2004).

14. De Las Rivas, J., Heredia, P. & Roman, A. Oxygen-evolving extrinsic proteins (*psb*O,P,Q,R): bioinformatic and functional analysis. *Biochim. Biophys. Acta.* **1767**, 575-582 (2007).
15. Ifuku, K., Ishihara, S., Shimamoto, R., Ido, K. & Sato, F. Structure, function, and evolution of the *psb*P protein family in higher plants. *Photosynth. Res.* **98**, 427-437 (2008).
16. Popelkova, H. & Yocum, C. F. *Psb*O, the manganese-stabilizing protein: analysis of the structure-function relations that provide insights into its role in photosystem II. *J. Photochem. Photobiol. B* **104**, 179-190 (2011).
17. Ifuku, K. The *psb*P and *psb*Q family proteins in the photosynthetic machinery of chloroplasts. *Plant Physiol. Biochem.* **81**, 108-114 (2014).
18. Chen, H. et al. A *psb*27 homologue in *Arabidopsis thaliana* is required for efficient repair of photodamaged photosystem II. *Plant Mol. Biol.* **61**, 567-575 (2006).
19. Nowaczyk, M. M. et al. *Psb*27, a cyanobacterial lipoprotein, is involved in the repair cycle of photosystem II. *Plant Cell.* **18**, 3121-1331 (2006).
20. Grasse, N. et al. Role of novel dimeric photosystem II (PSII)-*psb*27 protein complex in PSII repair. *J. Biol. Chem.* **286**, 29548-29555 (2011).
21. Kurisu, G., Zhang, H., Smith, J. L. & Cramer, W. A. Structure of the cytochrome b6f complex of oxygenic photosynthesis: tuning the cavity. *Science* **302**, 1009-1014 (2003).
22. Yasuda, R., Noji, H., Yoshida, M., Kinosita, Jr K. & Itoh, H. Resolution of distinct rotational substeps by submillisecond kinetic analysis of F1-ATPase. *Nature* **410**, 898-904 (2001).
23. Feniouk, B. A & Junge, W. Regulation of the F0F1-ATP synthase: the conformation of subunit ε might be determined by directionality of subunit γ rotation. *FEBS Lett.* **579**, 5114-5118 (2005).
24. Thornton, L. E., Roose, J. L. & Pakrasi, H. B. The low molecular weight proteins of photosystem II Ch. Photosystem II (Netherlands, Springer, 2005).
25. Dühring, U. Ossenbühl, F. & Wilde, A. Late assembly steps and dynamics of the cyanobacterial photosystem I. *J. Biol. Chem*. 282, 10915-10921 (2007).

26. Sato, N. & Moriyama, T. Photosynthesis *Ch. Cyanidioschyzon merolae: a new model eukaroytie for cell and organelle biology* (Singapore, Springer, 2017).
27. Ma, G. et al. Effect of red and blue LED light irradiation on ascorbate content and expression of genes related to ascorbate metabolism in postharvest broccoli. *Postharvest Biol. Tec.* **94**, 97-103 (2014).
28. Kim, D. et al. Tophat2: accurate alignment of transcriptomes in the presence of insertions, deletions and gene fusions. *Genome Biol.* **14**, R36 (2013).
29. Anders, S., Pyl, P. T. & Huber, W. HTSeq-a python framework to work with high-throughput sequencing data. *Bioinformatics* **31**, 166-169 (2015).
30. Fairchild, C. R. et al. Phycocyanin α-subunit phycocyanobilin lyase. *Proc. Natl. Acad. Sci. USA* 89, 7017-7021 (1992).
31. Li, H. et al. The sequence alignment/map format and SAMtools. *Bioinformatics* **25**, 2078-2079 (2009).
32. Li, H. A statistical framework for SNP calling, mutation discovery, association mapping and population genetical parameter estimation from sequencing data. *Bioinformatics* **27**, 2987-2993 (2010).
33. Li, H. & Durbin, R. Fast and accurate long-read alignment with Burrows-Wheeler transform. *Bioinformatics* **26**, 589-595 (2010).
34. Bhattacharya, D. et al. Genome of the red alga *Porphyridium purpureum*. *Nat. Commun.* **4**, 1941 (2013).
35. Matsuzaki, M. et al. Genome sequence of the ultrasmall unicellular red alga *Cyanidioschyzon merolae* 10D. *Nature* **428**, 653-657 (2004).
36. Schönknecht, G. et al. Gene transfer from bacteria and archaea facilitated evolution of an extremophilic eukaryote. *Science* **339**, 1207-1210 (2013).
37. Brawley, S. H. et al. Insights into the red algae and eukaryotic evolution from the genome of *Porphyra umbilicalis* (Bangiophyceae, Rhodophyta). *Proc. Natl. Acad. Sci. USA* **114**, E6361-E6370 (2017).
38. Collén, J. et al. Genome structure and metabolic features in the red seaweed *Chondrus crispus* shed light on evolution of the Archaeplastida. *Proc. Natl. Acad. Sci. USA* **110**, 5247-5252 (2013).
39. Lee, J. M. et al. Analysis of the draft genome of the red seaweed *Gracilariopsis chorda* provides insights into genome size evolution in Rhodophyta. *Mol. Biol. Evol.*

35, 1869-1886 (2018).

40. Keeling, P. J. et al. The Marine Microbial Eukaryote Transcriptome Sequencing Project (MMETSP): illuminating the functional diversity of eukaryotic life in the oceans through transcriptome sequencing. *PloS Biol.* **12**, e1001889 (2014).
41. Johnson, L. K., Alexander, H. & Brown, C. T. Re-assembly, quality evaluation, and annotation of 678 microbial eukaryotic reference transctriptomes. *GigaScience* doi:10.1101/323576 (2018).
42. Dobin, A. et al. STAR: ultrafast universal RNA-seq aligner. *Bioinformatics* **29**, 15-21 (2013).
43. Hoff, K. J., Lange, S., Lomsadze, A., Borodovsky, M. & Stanke, M. BRAKER1: unsupervised RNA-seq-based genome annotation with GeneMark-ET and AUGUSTUS. *Bioinformatics* **32**, 767-769 (2016).
44. Lomsadze, A., Burns, P. D. & Borodovsky, M. Integration of mapped RNA-seq reads into automatic training of eukaryotic gene finding algorithm. *Nucleic Acids Res.* **42**, e119 (2014).
45. Stanke, M. et al. AUGUSTUS: ab initio prediction of alternative transcripts. *Nucleic Acids Res.* **34**, W435-W439 (2006).
46. Marchler-Bauer, A. et al. CDD/SPARCLE: functional classification of proteins via subfamily domain architectures. *Nucleic Acids Res.* **45**, D200-203 (2017).
47. Emanuelsson, O., Nielsen, H. & von Heijne, G. ChloroP, a neural network-based method for predicting chloroplast transit peptides and their cleavage sites. *Protein Sci.* **8**, 978-984 (1999).
