# Supplementary material for: Expansion of phycobilisome linker gene families in mesophilic red algae
Source: Nat Commun. 2019 Oct 23;10:4823. doi: 10.1038/s41467-019-12779-1 (PMC6811547; doi:10.1038/s41467-019-12779-1)
Supplement: Supplementary file 3 — Reporting Summary [file 41467_2019_12779_MOESM3_ESM.pdf]

nature research

Corresponding author(s): NCOMMS-19-02099A

Last updated by author(s): Sep 2, 2019

Reporting Summary

Nature Research wishes to improve the reproducibility of the work that we publish. This form provides structure for consistency and transparency in reporting. For further information on Nature Research policies, see Authors & Referees and the Editorial Policy Checklist .

Statistics

For all statistical analyses, confirm that the following items are present in the figure legend, table legend, main text, or Methods section.

n/a	Confirmed	
☐	☒	The exact sample size (n) for each experimental group/condition, given as a discrete number and unit of measurement
☐	☒	A statement on whether measurements were taken from distinct samples or whether the same sample was measured repeatedly
☐	☒	The statistical test(s) used AND whether they are one- or two-sided<br>*Only common tests should be described solely by name; describe more complex techniques in the Methods section.*
☒	☐	A description of all covariates tested
☒	☐	A description of any assumptions or corrections, such as tests of normality and adjustment for multiple comparisons
☐	☒	A full description of the statistical parameters including central tendency (e.g. means) or other basic estimates (e.g. regression coefficient) AND variation (e.g. standard deviation) or associated estimates of uncertainty (e.g. confidence intervals)
☐	☒	For null hypothesis testing, the test statistic (e.g. F, t, r) with confidence intervals, effect sizes, degrees of freedom and P value noted<br>*Give P values as exact values whenever suitable.*
☒	☐	For Bayesian analysis, information on the choice of priors and Markov chain Monte Carlo settings
☒	☐	For hierarchical and complex designs, identification of the appropriate level for tests and full reporting of outcomes
☒	☐	Estimates of effect sizes (e.g. Cohen's d, Pearson's r), indicating how they were calculated

Our web collection on statistics for biologists contains articles on many of the points above.

Software and code

Policy information about availability of computer code

Data collection

1. Download illumina sequencing reads for hybrid assembly
https://www.ncbi.nlm.nih.gov/sra/SRX242705
2. Download RepeatModeler pipeline for repeat element analysis
http://www.repeatmasker.org/RepeatModeler
3. Download Repeat Library for repeat element analysis
http://www.girinst.org
4. Download EST data for phylogenetic analysis
https://zenodo.org/record/1212585#.XEvKvibsZCo
https://github.com/dib-lab/dib-MMETSP
5.Download Genome data for gene and phylogenetic analysis
https://www.ncbi.nlm.nih.gov/genome/
http://porphyra.rutgers.edu/bindex.php
6. Download Griffithsia pacifica phycobilisome antenna and linker proteins for analysis of phycobilisome gene families
https://www.ncbi.nlm.nih.gov/protein/?term=Structure+of+phycobilisome+from+the+red+alga+Griffithsia+pacifica
7. Download RNA sequencing data for gene-modeling
https://www.ncbi.nlm.nih.gov/sra/SRX242707

Data analysis

1. Albacore script for base calling of Nanopore sequencing reads
https://github.com/nickloman/nanopore-basecalling-scripts
https://nanoporetech.com/events/knowledge-exchange-installing-and-running-albacore
2. MaSuRCA assembler for hybrid assembly
https://github.com/alekseyzimin/masurca
3. BWA (Burrows-Wheeler Aligner) program for align DNA sequencing reads to genome assembly

http://bio-bwa.sourceforge.net/
4. Samtools for error correction of genoem assembly
https://github.com/samtools/samtools
5. STAR aligner for align RNAseq reads to genome assembly
https://github.com/alexdobin/STAR
6. BRAKER2 pipeline for gene-modeling
https://github.com/Gaius-Augustus/BRAKER
7. GeneMark-ET for gene-modeling
http://exon.gatech.edu/GeneMark/license_download.cgi
8. AUGUSTUS for gene-modeling
https://github.com/Gaius-Augustus/Augustus
9. BUSCO (Benchmarking Universal Single-Copy Orthologs) analysis for quality check of gene-models
https://busco.ezlab.org/v1/
10. MAFFT for align homolog gene family
https://mafft.cbrc.jp/alignment/software/
11. IQ-tree for Maximum Likelihood phylogeny analysis
http://www.iqtree.org/
12. Local BLAST program & web-based BLAST for search of homologous genes
https://blast.ncbi.nlm.nih.gov/Blast.cgi?CMD=Web&PAGE_TYPE=BlastDocs&DOC_TYPE=Download
https://blast.ncbi.nlm.nih.gov/Blast.cgi
13. KEGG annotations for gene analysis of metabolic pathways and functional annotations
https://www.kegg.jp/blastkoala/
15. Conserved Domain search for protein domain predictions
https://www.ncbi.nlm.nih.gov/Structure/bwrpsb/bwrpsb.cgi
16. ChloroP for chloroplast (plastid) transit peptide predictions
http://www.cbs.dtu.dk/services/ChloroP
17. Uniprot database information for search of protein functions
https://www.uniprot.org/
18. Tophat2 for RNA-seq mapping
https://github.com/dnanexus/tophat_cufflinks_rnaseq/tree/master/tophat2
19. HTSeq-count for RNA-seq read counts
https://github.com/simon-anders/htseq

For manuscripts utilizing custom algorithms or software that are central to the research but not yet described in published literature, software must be made available to editors/reviewers. We strongly encourage code deposition in a community repository (e.g. GitHub). See the Nature Research guidelines for submitting code & software for further information.

Data

Policy information about availability of data

All manuscripts must include a data availability statement. This statement should provide the following information, where applicable:

- Accession codes, unique identifiers, or web links for publicly available datasets
- A list of figures that have associated raw data
- A description of any restrictions on data availability

All these hybrid genome assembly, gene models, and functional annotations of P. purpureum are available at this link (http://porphyra.rutgers.edu/bindex.php), and NCBI (BioProject ID: PRJNA560054, Genome accession number VRMN00000000, RNA-seq data: SRR SRR10010265 ~ SRR10010276).

Field-specific reporting

Please select the one below that is the best fit for your research. If you are not sure, read the appropriate sections before making your selection.

☐ Life sciences ☐ Behavioural & social sciences ☒ Ecological, evolutionary & environmental sciences

For a reference copy of the document with all sections, see nature.com/documents/nr-reporting-summary-flat.pdf

Ecological, evolutionary & environmental sciences study design

All studies must disclose on these points even when the disclosure is negative.

Study description	In this study, our major finding is extensive duplications and diversification of nuclear-encoded phycobilisome linker proteins in mesophilic red algal genomes that constitute structure of phycobilisomes and play central roles in stabilizing light-harvesting functions. This finding was addressed by genome data analysis. We interpret these results as evidence that the origin of complex phycobilisome linker proteins in mesophilic red algae allowed them to adapt and radiate under different light regimes. In addition, we generated the high-quality hybrid genome assembly of Porphyridium purpureum based on Nanopore sequencing platform that it will provide a valuable platform to support future genome-based cell biological experiments.
Research sample	For this study, we ordered the unicellular red alga Porphyridium purpureum (strain: CCMP 1328) from the culture collection (NCMA, https://ncma.bigelow.org/) that was isolated from the coastal side pond in the North Atlantic ocean.

	Sex of this species is unknown. These algal samples were subcultured in L1-Si standard medium. Taxonomic information: Eukaryota, Rhodophyta (red algae), class Porphyridiophyceae, order Porphyridiales, genus Porphyridium
Sampling strategy	[N/A] In this study, there was no experiment to determine sample-size.
Data collection	For genome sequencing of our target species, we used the standard protocol described in the MinION library preparation kit (SQK-LSK109; Oxford Nanopore Technologies) by following these steps: DNA repair/end-prep without DNA fragmentation step, adapter ligation, and clean-up steps. After priming a new flow cell and loading the prepared sequencing library, the Nanopore sequencing run was progressed during 48 hours by the MinKNOW v1.14.1 platform (GUI v2.1.14). The base-calling of raw sequence data was conducted using the Albacore v2.3.1 script provided by Oxford Nanopore Technologies (https://nanoporetech.com).
Timing and spatial scale	[N/A] In this study, there was no experiment to require timing and spatial scale.
Data exclusions	[N/A] All available red algal Expressed Sequence Tag (EST) data and genome-based predicted genes in our target groups were used for this study.
Reproducibility	[N/A] In this study, there was no experiment related to reproducibility.
Randomization	[N/A] In this study, there was no experiment consider to randomization.
Blinding	[N/A] There was no blinding information in this study.

Did the study involve field work? ☐ Yes ☒ No

Reporting for specific materials, systems and methods

We require information from authors about some types of materials, experimental systems and methods used in many studies. Here, indicate whether each material, system or method listed is relevant to your study. If you are not sure if a list item applies to your research, read the appropriate section before selecting a response.

Materials & experimental systems

n/a	Involved in the study
☒	☐ Antibodies
☒	☐ Eukaryotic cell lines
☒	☐ Palaeontology
☒	☐ Animals and other organisms
☒	☐ Human research participants
☒	☐ Clinical data

Methods

n/a	Involved in the study
☒	☐ ChIP-seq
☒	☐ Flow cytometry
☒	☐ MRI-based neuroimaging
