# Supplementary material for: Expansion of phycobilisome linker gene families in mesophilic red algae
Source: Nat Commun. 2019 Oct 23;10:4823. doi: 10.1038/s41467-019-12779-1 (PMC6811547; doi:10.1038/s41467-019-12779-1)
Supplement: Supplementary file 4 — Description of Additional Supplementary Files [file 41467_2019_12779_MOESM4_ESM.docx]

**Description of Additional Supplementary Files**

File Name: Supplementary Data 1.
Description: 775 unique protein sequences in the long-read based assembly data (*: KEGG functions are also present in the short-read based assembly data).

File Name: Supplementary Data 2.
Description: Phylogenetic origins of phycobilisome proteins in red algal genomes.
