# Supplementary material for: Expansion of phycobilisome linker gene families in mesophilic red algae
Source: Nat Commun. 2019 Oct 23;10:4823. doi: 10.1038/s41467-019-12779-1 (PMC6811547; doi:10.1038/s41467-019-12779-1)
Supplement: Supplementary file 2 — Peer Review File [file 41467_2019_12779_MOESM2_ESM.pdf]

Reviewers' comments:

Reviewer #1 (Remarks to the Author):

This paper is important and timely. However, I feel that the high quality of the data deserves a better presentation in the text. Also, less sometimes is more: Concentration on the phycobilisome evolution and shift of the EGTs in the photosynthetic apparatus and the protein import system to a separate paper is an option…
Anyway, I suggest to modify and partially rewrite the manuscript:
20: …and that the origin of complex…
25: (plastids and mitochondria)
42: … and other accessary pigments organized in the macromolecular complexes called phycobilisomes)
44: …chlorophyll a/b proteins in Viridiplantae…
45 – 47: This sentence is redundant and could be omitted
47, 48: …with considerable modifications…
51: …light-harvesting antenna complexes…
54 – 57: This sentence could be omitted as it deals with commercial applications.
64, 65: Instead: An additional sentence would be helpful explaining the role of the gamma linkers bearing phycoerythrobilins (in contrast to "normal " linker proteins).
65: …the adhesion domain FAS1.
80: …through the development of flexible phycobilisome structures.
101: …several fold greater than…
150: Here, it should be mentioned that rhodophytes and glaucophytes share a primordial Toc translocon requiring a phenylalanine residue in the N-terminal domain of the transit peptides (Price et al., 2017)
155 – 170: These two paragraphs have to be rewritten to remove redundant sentences, correct wrong gene denominations, change the order of citations (and consequently of references*) etc.
161: The psbO and psbP genes…

164: …involved in the repair…
165, 166: …are petC (Rieske iron-sulfur protein in the cytochrome b6/f complex53), petH (ferredoxin: NADP+ oxidoreductase), and atpC (ATPase gamma subunit54,55)…

*53. Kurisu, G., Zhang, H., Smith, J. L. & Cramer, W. A. Structure of the cytochrome b6f complex of oxygenic photosynthesis: tuning the cavity. Science 302, 1009-1014 (2003).

*54. Yasuda, R., Noji, H., Yoshida, M., Kinosita, Jr K. & Itoh, H. Resolution of distinct rotational substeps by submillisecond kinetic analysis of F1-ATPase. Nature 410, 898-904 (2001).

*55. Feniouk, B. A & Junge, W. Regulation of the F0F1-ATP synthase: the conformation of subunit ε might be determined by directionality of subunit γ rotation. FEBS Lett. 579, 5114-5118 (2005).

173: In red algae (and likewise in glaucophytes), the majority of the components of the photosynthetic machinery is encoded…

179: …chlorophyll a/b proteins in Viridiplantae

180: …because these protein complexes are present…

182: …of the protein components…

183: We characterized red algal phycobilisome protein families…

206: …a globin superfamily-like domain…

217: …lumenal located…

251: …duplicated copies from an independent family…

273: …caeruleus…

301: Eurhodophytina

308-310: The lower number of glaucophyte phycobilisome proteins is due to the absence of phycoerythrin and its associated linker proteins. However, complex linker proteins likely originating from gene duplication have also been described for C. paradoxa: Price et al. (2017) Glaucophyta, in "Handbook of the Protists".

571: …of red algal phycobilisome protein families…

Fig. 2: There are more nuclear encoded linker proteins (seven in total) for C. paradoxa than shown in Fig. 2, see Price et al. (2017). It would have been desirable to include Griffithsia, that has been used as the reference organism with respect to phycobilisome structure throughout this paper!

Fig. 4: Phycobilisome antenna pigments
In the unrooted tree, the novel linker adjacent to LR3 should be "novel linker 3" (not 2)!

Suppl. Figs. S5, S6, S6b, S6c, S6d, S6e, S6f, S7, S9, S10: Compsopogon caeruleus
S9: Calliarthron tuberculosum

Reviewer #2 (Remarks to the Author):

The manuscript dives into the evolution of the phycobilisome linker proteins with a focus on red algae. The phycobilisomes are incredible structures and investigations into how they evolved, including the less understood liker polypeptides, is certainly of interest. The authors present a lot of data and the sheer volume of analyses are impressive! I also want to compliment the authors on the beautiful figures. These were very well presented and easy to follow. However, there could be some improvements to the manuscript so that the story being told is clearer and more succinct. For instance, the focus on EGT and the evolution of the TIC/TOC complexes plus the photosynthetic apparatus doesn't fit well in this paper and isn't entirely novel. I recommend removing these sections or at least explain how this work is related to the main story and its significance compared to other studies. Expanding on the phycobilisome and linker role in the introduction would also improve the flow of the story.

Figure 1:
The phylogeny used 4777 concatenated nuclear proteins and the outgroup used is non-photosynthetic. Did your analysis exclude all photosynthesis proteins? How were the proteins selected? The tree shows red algae basal in the Archaeplastida, which is contrary to other data. Perhaps discuss this more.

Figure 3:
The protein similarity network is great. However, the use of a cut off of 1e-5 is too low. The fact that the only edges are to LCM at this E value would indicate the similarity between the linkers and phycobiliproteins is poor. The question, then, is are these proteins really homologs? It wasn't clear from the manuscript and it's a point that needs to be addressed more specifically. This would have implications for the phylogeny in Figure 4 and suggest that a combined phylogeny wouldn't be appropriate. It would be helpful to have an alignment available in the supplementary information to assess this more easily.

General Comment:
In the conclusion the authors state "We postulate that the origin of complex phycobilisome linker structures in mesophilic red algae may have supported the radiation of these taxa into diverse environments after the initial phase of massive genome reduction. This, and other processes ultimately led to the origin of over 7,000 described mesophilic species." While linkers likely have some adaptive role in fine tuning light-harvesting capacity, it is but one small cog in a larger machine. I don't see the evidence that their impact would be great after genome reduction.

Specific, minor comments:

Line 39: Try to avoid using "light harvesting complex" collectively to refer to phycobilisomes and membrane integral light harvesting complexes (LHCs), as it confuses the terminology. Antenna seems like an appropriate term for this role.

The word "pigment" is used to refer to the phycobiliprotein and the chromophore interchangeable, and it is a little unclear. Here is one example (Line 177) "A
remarkable diversification and divergence are found in gene families that encode the light harvesting antenna pigments, including phycobilisomes in red algae and glaucophytes, and chlorophyll-b in Viridiplantae".

Line 46: Doesn't appear to cite the correct reference. Double check.

Line 181: "…phycobilisomes are more highly diverged in red algae". Since glaucophytes are poorly sampled, perhaps this is from sampling issues.

Line 204: Discussion of the novel phycobilisome protein seems to over-interpret the function based on similarity to other proteins.

Line 226: "This is because they share similar domain…" This is an important concept that needs to be explored in the introduction more. The reader needs to be introduced to these concepts in more detail.

Line 227-229: Point about the KEGG metabolic dataset is not clear. Refine explanation.

Overall, nice work. I would recommend that you focus on the phycobilisome linker story, bring more of that data from the supplementary files out into the main body of the manuscript.

Reviewer #3 (Remarks to the Author):

It was previously reported, that the red algal nuclear genome underwent a massive genome reduction before diversification into the extremophilic Cyanidophytina and the various mesophilic lineage. The authors of this manuscript improved the Porphyridium purpureum nuclear genome assembly (the

genome is 22 Mbp, with 9,902 gene models and 14% repeats) and reconstructed events associated with EGT and gene duplications. The new genome assembly is better than the previously reported assembly with respect to both completeness and quality. Furthermore, many genes that have undergone EGT are associated with core photosynthetic genes (the authors really mean the genes encoding major components of photosynthetic electron transport chain) with extensive diversification of the phycobilisome (PBS) linker polypeptides, which play an important role in both energy transfer and PBS stability. The authors suggest that diversification of the linkers allowed for radiation of the mesophilic reds into different ecological niches (based on the light environment).

There is some imprecision in discussing the PBS and linker polypeptides. Most of the linkers connect hexamers of phycobiliproteins (really hexamers of α and β subunits, which themselves are heterodimers) to each other; e.g. phycoerythrin-to-phycoerythrin hexamers, phycoerythrin-to-phycocyanin hexamers and phycocyanin-to-phycocyanin hexamers. These rod linkers (LR) are precisely positioned in the rod structure (specific hexamers in the rods based on position in the rod relative to the core). Some of these rod linkers may also function to terminate the rod at the distal location relative to the core and may even bind a chromophore, as suggested in the manuscript. Linkers also connect the rods to the core (the most proximal phycocyanin hexamer of the rods to the allophycocyanin trimers of the core; LRC) and the core allophycocyanin trimers to the membranes/reaction centers of the photosynthetic apparatus (LCM; these are the largest linkers and may be the most critical for coordinating interactions of the light harvesting structure with reaction centers). The linker classification that the authors give in the manuscript is based on the PBS structure of Griffithsia pacifica, which is a macrophytic red alga with different cell types and a complex morphology and life history. The organism which is being analyzed at the genomic level is Porphyridium purpureum, which is a unicellular red alga. The structural complexity of the PBS in some of the macrophytes versus the unicells may be considerably different. The authors talk about diversification based on light environments, but what about single cell versus multicellular? Are there major differences among the repertoire of linker polypeptides (based on gene models) among the various red alga and do they correlate with light or other aspects of the environment (e.g. desiccation of intertidal organisms), life history, single cell versus multicell etc.

It is not surprising that many genes encoding proteins associated with photosynthesis moved to the nuclear genome by EGT. This has been known for genes of the green and red lineages for many years and has been characterized extensively in both land plants and green alga. There is a greater proportion of the major electron transport genes that have moved to the nucleus in the red algae relative to the green lineage (not really overall genes for photosynthesis as suggested), but neither genes encoding Calvin-Bassham Benson Cycle enzymes (also part of photosynthesis) nor genes for the biosynthesis of chlorophyll and carotenoids, the biogenesis of the cytochromes, the regulation of translation and stability of the plastid transcripts for photosynthesis genes etc are considered in the analysis. If considered, genes for well over half of the proteins of the photosynthetic machinery has moved to the nucleus for both the green lineage and the red alga; it all depends on what you are calling the photosynthetic machinery.
There are also interesting aspects of the data that are not discussed. For example, there are several ‘photosynthesis’ genes that are not present on either the plastid or nuclear genomes of the red alga but are present in green algae and plants: these genes include PETE, which encodes for plastocyanin, PSAN, which encodes the protein for docking plastocyanin, and PSAG, which is for electron transport between plastocyanin and PSI. Plastocyanin is required for the functioning of the photosynthetic electron transport chain and so you either must have it or have a substitute for it… several organisms can use a c6 type cytochrome instead of plastocyanin. So the reds must have a cytochrome c6-type protein and perhaps accessory proteins associated with it. This has implications with respect to the metals used by the organisms… plastocyanin requires copper and cytochrome c6 iron. Perhaps this should be part of the discussion? Other genes missing are PSAH, which docks light harvesting complex I antenna (the reds don’t have the same LHCAI as the greens), PSBS, which is required for quenching in the chlorophyll based antenna of PSII (the reds have the PBS and not the major chlorophyll based antenna) and PSBR, which is needed for the stable assemble of the chlorophyll based antenna of PSII

(again the reds don’t have these antenna). The paper would benefit from a more probing analysis of some of the data that is being presented.

As the authors state, there has been diversification of the genes encoding linker polypeptides of the PBS. The line on 190-192 says that the core membrane linker proteins connect the rod linker (LR) and the rod core linker (LRC)… but I think that they connect the PBS core to the core complex of reaction centers within the membranes. The authors also extended the analysis of the novel PBS pigmented protein with a globin domain (previously identified in the P. purpureum genome) to other members of the Proteorhodophytina, and found 3 novel Nu-PBS linkers, which likely diversified after the split from the Cyanidiophytina.
The LR1 gene (similar to LR genes in cyanobacteria) was likely the gene inherited from the cyanobacteria and then underwent EGT and diversified giving rise to the larger linker family in red algae. The movement of linker genes to the nucleus and their diversification in the different red algal subphyla is interesting, although the functional significance of this diversification is not known, even though it does appear that the linkers are associated with both efficient energy transfer within the complex and its stability. The authors suggest that diversification of the linkers supported the radiation of the phyla into diverse environments following the initial massive genome reduction. At this point is there really any strong evidence to support this conjecture? Could it be that changes in other cellular processes provoked diversification of the linkers? If the diversification of the linkers was an adaptation that allowed the red algae to thrive in various light environments, why didn’t this diversification occur in the cyanobacteria which can live in almost every niche available on our planet (even at 70-75oC in the hot springs)? Hence a major conclusion of the paper appears to me to be highly speculative, with the only support being the ‘diversification’ itself. It might also be interesting to carefully examine the linkers among all of the reds and see if any of their features do correlate with the physiological characteristics of the different organisms (light, desiccation, life cycle and morphology etc).
Overall, while there is some interesting data in this paper and that various aspects of the evolution of the various photosynthesis genes may be interesting, I feel that the results require more analyses and the major conclusion are speculative with little support. At this point, my opinion is that this work would be a valuable contribution to a more specialized journal, especially if the data was more carefully considered and discussed.

Reviewer #4 (Remarks to the Author):

This paper attempts to clarify how mesophilic red algae diversified. The new data of Porphyridium purpureum is derived from Nanopore long-reads. The hybrid assembly with Illumina reads provides the high quality genome. By molecular phylogenetic analysis using large data sets, the authors show extensive duplications of phycobilisome linker proteins in ancestral mesophilic red algae. They claim that early endosymbiotic gene transfer of the linker protein and the duplication may have played critical roles for the diversification of mesophilic red algae. The finding of expanded linker proteins is novel and seems the most interesting. The paper is clearly written and the results are presented in a clear manner. I’m convinced the conclusions are sufficient novel to warrant publication in Nature Communications. I have some concerns.

Major remarks

L152-L154: The analysis of ancient EGTs is interest to researchers in wider-ranging field. For comparison, some analyses of other TIC/TOC proteins have to indicate using the high quality data sets. In addition, gene trees supporting eukaryotic-specific and land plant-specific origins are useful to interpret the results for ancient EGT origin in Figs. 2, S5 and S6.

L19-L22, L77-80, and L310-L312: The question for diversification of mesophilic red algae is interesting. Comparative genomic analysis by multiple methods indicates new results. But the

discussion on the adaptation to diverse environment is ambiguous. Although transcriptome analyses may be beyond the scope of their analysis, the expression data of linker genes in different environments may support the discussion.

Minor remarks

L77-L80: If the explanation for red algal group, including Proteorhodophytina and Eurhodophytina, is added in the introduction, it is likely to be useful to understand the interesting point of this research.

L97-L99: There is no verb.

L130-144: Fig.1 has to be referred in the paragraph.

L155-L157 and Figure 2: If the information of conserved photosynthetic proteins in extant cyanobacteria is added, it is helpful to understand the important of the analysis.

L188-L192: I feel that the network analysis with figure 3 needs to explain more clearly.

L243: Are plastid-targeting signals found in novel Nu-PBS linker sequences?

L379-L381: Does the hybrid genome assembly have accession number of GenBank?

Figure 4: “Phycobilisoem” is typo.

The point-by-point responses

Reviewers' comments:

Reviewer #1 (Remarks to the Author):

This paper is important and timely. However, I feel that the high quality of the data deserves a better presentation in the text. Also, less sometimes is more: Concentration on the phycobilisome evolution and shift of the EGTs in the photosynthetic apparatus and the protein import system to a separate paper is an option… Anyway, I suggest to modify and partially rewrite the manuscript:

We appreciate your thoughtful and valuable comments. Based on these suggestions, we have thoroughly revised the manuscript.

20: …and that the origin of complex…
25: (plastids and mitochondria)
42: … and other accessary pigments organized in the macromolecular complexes called phycobilisomes)
44: …chlorophyll a/b proteins in Viridiplantae…
45 – 47: This sentence is redundant and could be omitted
47, 48: …with considerable modifications…
51: …light-harvesting antenna complexes…

We made all of these changes as suggested.

54 – 57: This sentence could be omitted as it deals with commercial applications.

We omitted two references to commercial applications, and revised the text as follows; "…elucidated from the unicellular red alga *Porphyridium*[10,15,16]. For instance, from *P. cruentum* (a synonym of *P. purpureum*; www.algaebase.org), the basis of functional stability across a wide pH range (pH 4 ~ 8) has been explained by pH-dependent structural conformations of the B-phycoerythrin protein complex[17,18]."

64, 65: Instead: An additional sentence would be helpful explaining the role of the gamma linkers bearing phycoerythrobilins (in contrast to "normal "linker proteins).

We revised the sentence as follows: "LR-gamma4 ~ LR-gamma8 comprising the phycoerythrin gamma chain linker polypeptide containing the conserved chromophore binding…".

65: …the adhesion domain FAS1.
80: …through the development of flexible phycobilisome structures.
101: …several fold greater than…
We made all of these changes as suggested.

150: Here, it should be mentioned that rhodophytes and glaucophytes share a primordial Toc translocon requiring a phenylalanine residue in the N-terminal domain of the transit peptides (Price et al., 2017)

We added this information to the section on ancient EGT, that was moved into Supplementary Text, as suggested by Reviewer 2.

155 – 170: These two paragraphs have to be rewritten to remove redundant sentences, correct wrong gene denominations, change the order of citations (and consequently of references*) etc.
165, 166: …are petC (Rieske iron-sulfur protein in the cytochrome b6/f complex53), petH (ferredoxin: NADP+ oxidoreductase), and atpC (ATPase gamma subunit54,55)…

We reorganized the references, and corrected typos and gene denominations as suggested. The 'ancient EGT' section was moved into Supplementary Text, as reviewer 2 suggested. We briefly described these results in the main text as follows; "These differences are explained by differential EGT or outright gene loss in the ancestors of each lineage[8,27]. To generate a more complete picture of the gene loss process, we analyzed EGTs that putatively (i.e., excluding parallel transfers) occurred before the diversification of the three Archaeplastida lineages (Fig. 2 and Supplementary Fig. S4). This analysis shows that genes with several core plastid functions including the TIC/TOC complex and several involved in photosynthesis (PSII, cytochrome, ferredoxin, and ATPase complexes) were moved to the nuclear genome of primordial algae (Supplementary Table S7, Figs. S5 and S6; see details in Supplementary Text). A remarkable diversification and divergence are found in gene families that encode the light-harvesting antenna…".

161: The psbO and psbP genes…
164: …involved in the repair…
173: In red algae (and likewise in glaucophytes), the majority of the components of the photosynthetic machinery is encoded…
179: …chlorophyll a/b proteins in Viridiplantae
180: …because these protein complexes are present…
182: …of the protein components…
183: We characterized red algal phycobilisome protein families…
206: …a globin superfamily-like domain…
217: …lumenal located…
251: …duplicated copies from an independent family…
273: …caeruleus…
301: Eurhodophytina
571: …of red algal phycobilisome protein families…

We made all of these changes as suggested.

308-310: The lower number of glaucophyte phycobilisome proteins is due to the absence of phycoerythrin and its associated linker proteins. However, complex linker proteins likely originating from gene duplication have also been described for C. paradoxa: Price et al. (2017) Glaucophyta, in "Handbook of the Protists".

We revised the sentence as follows; "These patterns are distinct from the phycobilisome-containing Glaucophyta. The model glaucophyte *Cyanophora paradoxa* contains a limited phycobilisome machinery (i.e., apcA-F, cpcA-B, cpcF-G) comprising gene duplication-derived phycobilisome linker proteins (Fig. 2 and Supplementary Fig. S8)[40,41].".

Fig. 2: There are more nuclear encoded linker proteins (seven in total) for C. paradoxa than

shown in Fig. 2, see Price et al. (2017). It would have been desirable to include Griffithsia, that has been used as the reference organism with respect to phycobilisome structure throughout this paper!

We are in agreement that *C. paradoxa* is a good model to study phycobilisome complexes but this species belongs to a different lineage compared to our target red algae. Furthermore, there is a single available genome from glaucophyte algae. Therefore, we thought that it was less critical to include *C. paradoxa* in our analysis. Instead, we used the well-characterized red algal model for the analysis of phycobilisome linkers (i.e., *Griffithsia*). We stated the limitation of predictions for phycobilisome linker proteins in glaucophytes and rhodophytes as follows; "This is because they share similar domain compositions, making it hard to identify individual family members using sequence data. Furthermore, the KEGG phycobilisome database is not a good predictive tool because it is primarily based on cyanobacterial genome data and lacks information from diverse red algal and glaucophyte phycobilisome linker proteins (Supplementary Table S7 and S8)[19,40]. To overcome these hurdles…".
Thank you for this suggestion. It would clearly be of value to focus on glaucophyte phycobilisomes in a separate paper.

Fig. 4: Phycobilisome antenna pigments
In the unrooted tree, the novel linker adjacent to LR3 should be "novel linker 3" (not 2)!
Suppl. Figs. S5, S6, S6b, S6c, S6d, S6e, S6f, S7, S9, S10: Compsopogon caeruleus
S9: Calliarthron tuberculosum

We corrected these errors as suggested.

Reviewer #2 (Remarks to the Author):
The manuscript dives into the evolution of the phycobilisome linker proteins with a focus on red algae. The phycobilisomes are incredible structures and investigations into how they evolved, including the less understood liker polypeptides, is certainly of interest. The authors present a lot of data and the sheer volume of analyses are impressive! I also want to compliment the authors on the beautiful figures. These were very well presented and easy to follow. However, there could be some improvements to the manuscript so that the story being told is clearer and more succinct. For instance, the focus on EGT and the evolution of the TIC/TOC complexes plus the photosynthetic apparatus doesn't fit well in this paper and isn't entirely novel. I recommend removing these sections or at least explain how this work is related to the main story and its significance compared to other studies. Expanding on the phycobilisome and linker role in the introduction would also improve the flow of the story.

We appreciate these valuable comments. In response, we have revised the manuscript to focus more on phycobilisome and linker proteins, and moved the EGT and TIC/TOC complex story to the Supplementary Text. We kept Fig. 2, including not only phycobilisomes but also other photosynthetic proteins to present a complete picture of the photosynthetic complex.

Figure 1:
The phylogeny used 4777 concatenated nuclear proteins and the outgroup used is non-photosynthetic. Did your analysis exclude all photosynthesis proteins? How were the proteins selected? The tree shows red algae basal in the Archaeplastida, which is contrary to other

data. Perhaps discuss this more.

We used only nuclear genes but excluded photosynthetic genes in this analysis. We chose nuclear genes that are shared in more than 10 species among the sampled taxa. We added this information in the text as follows; "We constructed a phylogenetic tree of Archaeplastida lineages based on a concatenated dataset of 4,777 nuclear proteins (excluding those involved in photosynthesis) that are shared by at least 10 species among the 28 sampled taxa.".

To date, this relationship '(Rhodophyta) and (Viridiplantae + Glaucophyta)' is widely supported by transcriptome-based gene- and taxon-rich datasets (Burki et al. 2016, *Proceedings of the Royal Society B: Biological Sciences* 283: 20152802) as well as plastid genome data (Lee et al. 2016, *BMC Biology* 14:75). We revised the text as follows; "This ML tree provides strong bootstrap support (BS) for a monophyletic Rhodophyta that is sister to the monophyletic groups, Viridiplantae and Glaucophyta (BS = 100%, Fig. 1). The interrelationships of these three primary endosymbiosis groups (i.e., the early split of Rhodophyta) is also supported by transcriptome-based gene and taxon-rich datasets[38] as well as by phylogenetic analysis of plastid genome data[8]. Within Rhodophyta, the subphylum…".

Figure 3:

The protein similarity network is great. However, the use of a cut off of 1e-5 is too low. The fact that the only edges are to LCM at this E value would indicate the similarity between the linkers and phycobiliproteins is poor. The question, then, are these proteins really homologs? It wasn't clear from the manuscript and it's a point that needs to be addressed more specifically. This would have implications for the phylogeny in Figure 4 and suggest that a combined phylogeny wouldn't be appropriate. It would be helpful to have an alignment available in the supplementary information to assess this more easily.

The LCM-connected relationship between PBS antenna and linkers results from the gene structure of LCM proteins that share both homologous parts with PBS antenna and linkers. There are several low *e*-values (1.e-06) between LCM and antenna proteins but the differences of *e*-values between LCM and others (antenna: 1.e-06 ~ -13 and linkers: 1.e-11 ~ -25) are caused by inter-species relationships or partially predicted low quality genes from some red algal genomes. For example, *Galdieria* (Cyanidiophytina) ApcD gene shows a relatively low *e*-value (1.e-06) with *Gracilariopsis* (Eurhodophytina) LCM gene but a higher *e*-value (1.e-10) with *Cyanidioschyzon* (Cyanidiophytina) LCM gene. Based on your comment, we conducted the same analysis with a more stringent *e*-value cutoff (1.e-10), and the result (Supplementary Figure S7) was consistent with current network connections (Fig. 3). Because the *e*-value cutoff 1.e-05 is widely used in homologous gene family analysis, we kept Fig. 3 with the addition of an alignment, but added Supplementary Figure S7 (*e*-value cutoff = 1.e-10). We revised the main text as follows; "This component contains a central node comprising core-membrane linker proteins (LCMs) that connects the LR (rod linker) and LRC (rod-core linker) protein families (Fig. 3; see details in Methods). The LCMs include sequences homologous with the phycobilisome antenna and other linker proteins (Fig. 3a, *e*-value cutoff=1.e-05). These network connections are also present when using a more stringent cutoff (*e*-value cutoff=1.*e*-10, Supplementary Fig. S7). The phycobilisome antenna proteins align well with the N-terminal region of LCMs, whereas linker proteins (i.e., LR and LRC) share similarity with the C-terminus of LCMs (Fig. 3b). Although aligned amino acid sequences between the phycobilisome antenna and linker proteins show low overall similarity (*e*-value>1.e-05), these two antenna and linker proteins share several well-conserved amino acid residues (Fig. 3b). Therefore, we used the alignment to construct the phylogeny of LMC-related genes to study the relationships of red algal phycobilisome protein families (Fig. 4).".

General Comment:

In the conclusion the authors state “We postulate that the origin of complex phycobilisome linker structures in mesophilic red algae may have supported the radiation of these taxa into diverse environments after the initial phase of massive genome reduction. This, and other processes ultimately led to the origin of over 7,000 described mesophilic species.” While linkers likely have some adaptive role in fine tuning light-harvesting capacity, it is but one small cog in a larger machine. I don’t see the evidence that their impact would be great after genome reduction.

We revised the sentence as follows; “We speculate that the origin of complex phycobilisome linker structures may have been selected for and thereby supported the radiation of mesophilic rhodophytes into diverse environments. In addition, the phycobilisomes, with their diverse linker proteins enables red algae to adapt to different light environments via chromatic adaptation[48-51]. This dynamic process may partly explain the provenance of the current 7,100 described mesophilic red algal species.”.

Specific, minor comments:

Line 39: Try to avoid using “light harvesting complex” collectively to refer to phycobilisomes and membrane integral light harvesting complexes (LHCs), as it confuses the terminology. Antenna seems like an appropriate term for this role.

We tried to use separate terms for phycobilisomes and light-harvesting chlorophyll *a*/*b* proteins (LHCP), and revised the text.

The word “pigment” is used to refer to the phycobiliprotein and the chromophore interchangeable, and it is a little unclear. Here is one example (Line 177) “A remarkable diversification and divergence are found in gene families that encode the light harvesting antenna pigments, including phycobilisomes in red algae and glaucophytes, and chlorophyll-b in Viridiplantae”.

Several un-specified words were revised as “light-harvesting antenna complexes”.

Line 46: Doesn’t appear to cite the correct reference. Double check.

The sentence was omitted.

Line 181: “…phycobilisomes are more highly diverged in red algae”. Since glaucophytes are poorly sampled, perhaps this is from sampling issues.

Indeed, there is sampling issue because there is only single available glaucophyte genome data (*Cyanophora paradoxa*). Nevertheless, the limited phycobilisome machinery and linker proteins of this species were compared to red algae in previous papers (references 40 and 41). We added these reports and the sampling limitation in the text as follows; “The phycobilisomes are more highly diverged in red algae, suggesting independent diversification of these protein components after divergence from Glaucophyta, which contains a limited phycobilisome machinery. Glaucophytes encode a small number of phycobilisome linker proteins, although this insight comes from a single completed nuclear genome, that of *Cyanophora paradoxa* (Figs. 1-2)[40,41]. We characterized red algal phycobilisome protein

families…"

Line 204: Discussion of the novel phycobilisome protein seems to over-interpret the function based on similarity to other proteins.

We tone down the interpretation as follows; "…analysis using the nr database returns the same result. It is, however, still unclear if cryptophyte allophycocyanin is fully functional because it has not been reported from these taxa[9,45-47].".

Line 226: "This is because they share similar domain…" This is an important concept that needs to be explored in the introduction more. The reader needs to be introduced to these concepts in more detail.

We added this concept in the introduction as follows; "Although a diversity of antenna pigments of phycobilisomes have been studied at the sequence-level[20-23], linker protein families in red algae remain poorly characterized because their similar domain compositions and the cyanobacteria-biased functional database (e.g., KEGG) make it challenging to generate detailed knowledge about these proteins.".

Line 227-229: Point about the KEGG metabolic dataset is not clear. Refine explanation.

We revised the sentences as follows; "This is because they share similar domain compositions, making it hard to identify individual family members using sequence data. Furthermore, the KEGG phycobilisome database is not a good predictive tool because it is primarily based on cyanobacterial genome data and lacks information from diverse red algal and glaucophyte phycobilisome linker proteins (Supplementary Table S7 and S8)[19,40]. To overcome these hurdles…".

Overall, nice work. I would recommend that you focus on the phycobilisome linker story, bring more of that data from the supplementary files out into the main body of the manuscript.

Based on your comments, we focus on the phycobilisome story in the main text and discussion of other photosynthetic machineries was moved into the Supplementary Text. We discussed in more detail the phycobilisome story, but mentioned the ancient EGT story briefly in the main text as follows; "These differences are explained by differential EGT or outright gene loss in the ancestors of each lineage[8,27]. To generate a more complete picture of the gene loss process, we analyzed EGTs that putatively (i.e., excluding parallel transfers) occurred before the diversification of the three Archaeplastida lineages (Fig. 2 and Supplementary Fig. S4). This analysis shows that genes with several core plastid functions including the TIC/TOC complex and several involved in photosynthesis (PSII, cytochrome, ferredoxin, and ATPase complexes) were moved to the nuclear genome of primordial algae (Supplementary Table S7, Figs. S5 and S6; see details in Supplementary Text). A remarkable diversification and divergence are found in gene families that encode the light-harvesting antenna complexes…".

Reviewer #3 (Remarks to the Author):

It was previously reported, that the red algal nuclear genome underwent a massive genome reduction before diversification into the extremophilic Cyanidophytina and the various mesophilic lineage. The authors of this manuscript improved the Porphyridium purpureum

nuclear genome assembly (the genome is 22 Mbp, with 9,902 gene models and 14% repeats) and reconstructed events associated with EGT and gene duplications. The new genome assembly is better than the previously reported assembly with respect to both completeness and quality. Furthermore, many genes that have undergone EGT are associated with core photosynthetic genes (the authors really mean the genes encoding major components of photosynthetic electron transport chain) with extensive diversification of the phycobilisome (PBS) linker polypeptides, which play an important role in both energy transfer and PBS stability. The authors suggest that diversification of the linkers allowed for radiation of the mesophilic reds into different ecological niches (based on the light environment).

There is some imprecision in discussing the PBS and linker polypeptides. Most of the linkers connect hexamers of phycobiliproteins (really hexamers of α and β subunits, which themselves are heterodimers) to each other; e.g. phycoerythrin-to-phycoerythrin hexamers, phycoerythrin-to-phycocyanin hexamers and phycocyanin-to-phycocyanin hexamers. These rod linkers (LR) are precisely positioned in the rod structure (specific hexamers in the rods based on position in the rod relative to the core). Some of these rod linkers may also function to terminate the rod at the distal location relative to the core and may even bind a chromophore, as suggested in the manuscript. Linkers also connect the rods to the core (the most proximal phycocyanin hexamer of the rods to the allophycocyanin trimers of the core; LRC) and the core allophycocyanin trimers to the membranes/reaction centers of the photosynthetic apparatus (LCM; these are the largest linkers and may be the most critical for coordinating interactions of the light harvesting structure with reaction centers). The linker classification that the authors give in the manuscript is based on the PBS structure of Griffithsia pacifica, which is a macrophytic red alga with different cell types and a complex morphology and life history. The organism which is being analyzed at the genomic level is Porphyridium purpureum, which is a unicellular red alga. The structural complexity of the PBS in some of the macrophytes versus the unicells may be considerably different. The authors talk about diversification based on light environments, but what about single cell versus multicellular? Are there major differences among the repertoire of linker polypeptides (based on gene models) among the various red alga and do they correlate with light or other aspects of the environment (e.g. desiccation of intertidal organisms), life history, single cell versus multicell etc.

We considered differences between macro- and micro-red algae with regard to phycobilisome structures. However, our analyses (e.g., Fig. 4 of PBS phylogeny) suggest that all mesophilic red algae (including unicells and multicells) share essentially the same set of phycobilisome and PBS linker proteins. Only the early diverged Cyanidiophytina contain a limited number of proteins (Supplementary Figure S8). Furthermore, regions of 3D structures of the phycobilisome in the target unicellular species of *P. purpureum* are similar to *Griffithsia* (Reference 10: Bryant and Canniffe 2018). In the figure, description for 'block-shaped PBS' of the red alga *Griffithsia pacifica*, Bryant and Canniffe (2018) notes that "These 'block-shaped PBS' are similar in appearance to the 'hemi-ellipsoidal PBS' of *Porphyridium cruentum*…". We also believe that inferences about cell type-dependent life histories are hard to link with our results. Additional analyses are needed. We added this limitation in the conclusion as follows; "Experiments aimed at elucidating gene function from diverse (i.e., unicellular, and multi-cellular) red algal species are needed to test these hypotheses.".

In addition, we added supplemental explanations for these cellular types of mesophilic red algal groups in the introduction; "We determined the origins of phycobilisome linker protein families including novel linker proteins in the mesophilic red algae. Although these taxa show different cellular life styles and plastid genome structures (i.e., subphylum Proteorhodophytina: unicellular/filamentous algae including intron-rich plastid genomes, and

Eurhodophytina: macroscopic seaweeds)[24], they share a diversity of conserved, lineage-specific nuclear-encoded phycobilisome linker protein families. Based on these data, we speculate that the ancestor of the mesophilic lineages was under selection to adapt to widely different light environments through the development of flexible phycobilisome structures.".

It is not surprising that many genes encoding proteins associated with photosynthesis moved to the nuclear genome by EGT. This has been known for genes of the green and red lineages for many years and has been characterized extensively in both land plants and green alga. There is a greater proportion of the major electron transport genes that have moved to the nucleus in the red algae relative to the green lineage (not really overall genes for photosynthesis as suggested), but neither genes encoding Calvin-Bassham Benson Cycle enzymes (also part of photosynthesis) nor genes for the biosynthesis of chlorophyll and carotenoids, the biogenesis of the cytochromes, the regulation of translation and stability of the plastid transcripts for photosynthesis genes etc are considered in the analysis. If considered, genes for well over half of the proteins of the photosynthetic machinery has moved to the nucleus for both the green lineage and the red alga; it all depends on what you are calling the photosynthetic machinery.

We previously defined the boundary of photosynthetic machineries [photosynthesis proteins (KEGG map00195: Photosynthesis)] that can be usually predicted based on KEGG database or BLAST tools. Currently, we divided this part into antenna complexes (main text; Fig. 2) and photosynthesis (Supplementary Text; Fig. 2) because our main focus is the phycobilisome linker protein families in red algae, which was also suggested by other reviewers. Instead, we added details in Supplementary Text to describe some Viridiplantae-specific photosynthetic genes that are absent in rhodophytes and glaucophytes as you pointed out.

There are also interesting aspects of the data that are not discussed. For example, there are several 'photosynthesis' genes that are not present on either the plastid or nuclear genomes of the red alga but are present in green algae and plants: these genes include PETE, which encodes for plastocyanin, PSAN, which encodes the protein for docking plastocyanin, and PSAG, which is for electron transport between plastocyanin and PSI. Plastocyanin is required for the functioning of the photosynthetic electron transport chain and so you either must have it or have a substitute for it… several organisms can use a c6 type cytochrome instead of plastocyanin. So the reds must have a cytochrome c6-type protein and perhaps accessory proteins associated with it. This has implications with respect to the metals used by the organisms… plastocyanin requires copper and cytochrome c6 iron. Perhaps this should be part of the discussion? Other genes missing are PSAH, which docks light harvesting complex I antenna (the reds don't have the same LHCAI as the greens), PSBS, which is required for quenching in the chlorophyll based antenna of PSII (the reds have the PBS and not the major chlorophyll based antenna) and PSBR, which is needed for the stable assemble of the chlorophyll based antenna of PSII (again the reds don't have these antenna). The paper would benefit from a more probing analysis of some of the data that is being presented.

We appreciate these valuable comments. These genes, which are absent in rhodophytes and glaucophytes as you have pointed out (i.e., *psa*N, *psa*G, *psa*H, *psb*S, *psb*R, and *pet*E) are Viridiplantae-specific that are also absent in cyanobacterial genomes (except the *pet*E gene; Fig. 2)[#1,#2]. Dühring et al.[#1] mentioned as '… Their primary structures are well conserved among eukaryotes and prokaryotes performing oxygenic photosynthesis with the exception of five subunits not present in cyanobacteria (PsaG, PsaH, PsaN, PsaO, and PsaP)…'. Thornton

et al.[#2] noted that '…The integral PSII subunit, psbS, is a 22 kDa protein found in plants and not in cyanobacteria … psbR is a 10 kDa extrinsic protein found only in plants …". In addition, *pet*E (plastocyanin) gene is likely Viridiplantae and cyanobacterial specific gene, and Cytochrome c6 encoded by *pet*J gene fulfill the role of plastocyanin in red algae that was reported by Sato and Moriyama[#3]. Sato and Moriyama[#3] mentioned that '… In contrast with green plants, red algae lack plastocyanin, a copper-bearing protein. Cytochrome c6 (encoded by the petJ gene) fulfills the role of plastocyanin in red algae, transferring electrons from the cytochrome b6f complex to PS I. In cyanobacteria and green algae, both plastocyanin and cytochrome c6 are present, but the latter functions mainly under copper deficiency, where the synthesis of plastocyanin is repressed. …'.

Therefore, our results are reasonable. We added some text in this regard: "Several photosynthetic genes (i.e., *psb*R, *psb*S, *psa*H, *psa*N, *psa*G, and *pet*E), present only in Viridiplantae, are involved in PSII, PSI, and the cytochrome complex but these genes (except *pet*E) are absent in cyanobacterial genomes (Fig. 2)[24,25]. Although these genes are important in photosynthetic functions of Viridiplantae, they may not be necessary in rhodophytes and glaucophytes as well as in cyanobacteria[24,25]. Furthermore, the *pet*E (plastocyanin) gene, which is involved in the functioning of the photosynthetic electron transport chain, is Viridiplantae- and cyanobacterial-specific. In red algae, however, cytochrome-*c*6 encoded by the *pet*J gene is the alternative for the plastocyanin[26]. This suggests that the phycobilisome-containing primary endosymbiosis groups (i.e., rhodophytes and glaucophytes) have different evolutionary history from Viridiplantae lineage that uses the light-harvesting antenna complex (i.e., chlorophyll a/b proteins).".

#1. Dühring, U. Ossenbühl, F. & Wilde, A. Late assembly steps and dynamics of the cyanobacterial photosystem I. J. Biol. Chem. 282, 10915-10921 (2007).
#2. Thornton, L. E., Roose, J. L. & Pakrasi, H. B. The low molecular weight proteins of photosystem II Ch Photosystem II (Netherlands, Springer, 2005).
#3 Sato, N. & Moriyama, T. Photosynthesis *Ch. Cyanidioschyzon merolae: a new model eukaroytie for cell and organelle biology* (Singapore, Springer, 2017).

As the authors state, there has been diversification of the genes encoding linker polypeptides of the PBS. The line on 190-192 says that the core membrane linker proteins connect the rod linker (LR) and the rod core linker (LRC)… but I think that they connect the PBS core to the core complex of reaction centers within the membranes.

The network connections are based on protein similarity, not physical (architectural) relationships within the membranes (although, in some cases, shared specific amino acid sites represent their physical interaction). We described these results more details in the text as follows; "This component contains a central node comprising core-membrane linker proteins (LCMs) that connects the LR (rod linker) and LRC (rod-core linker) protein families (Fig. 3; see details in Methods). The LCMs include sequences homologous with the phycobilisome antenna and other linker proteins (Fig. 3a, *e*-value cutoff=1.e-05). These network connections are present when using a more stringent cutoff (*e*-value cutoff=1.*e*-10, Supplementary Fig. S7). The phycobilisome antenna proteins align well with the N-terminal region of LCMs, whereas linker proteins (i.e., LR and LRC) share similarity with the C-terminus of LCMs (Fig. 3b). Although aligned amino acid sequences between the phycobilisome antenna and linker proteins show low overall similarity (*e*-value>1.e-05), these two antenna and linker proteins share several well-conserved amino acid sites (Fig. 3b). Therefore, we used the alignment to construct the phylogeny of LMC-related genes to study the relationships of red

algal phycobilisome protein families (Fig. 4).”.

The authors also extended the analysis of the novel PBS pigmented protein with a globin domain (previously identified in the P. purpureum genome) to other members of the Proteorhodophytina, and found 3 novel Nu-PBS linkers, which likely diversified after the split from the Cyanidiophytina.

The LR1 gene (similar to LR genes in cyanobacteria) was likely the gene inherited from the cyanobacteria and then underwent EGT and diversified giving rise to the larger linker family in red algae. The movement of linker genes to the nucleus and their diversification in the different red algal subphyla is interesting, although the functional significance of this diversification is not known, even though it does appear that the linkers are associated with both efficient energy transfer within the complex and its stability. The authors suggest that diversification of the linkers supported the radiation of the phyla into diverse environments following the initial massive genome reduction. At this point is there really any strong evidence to support this conjecture? Could it be that changes in other cellular processes provoked diversification of the linkers? If the diversification of the linkers was an adaptation that allowed the red algae to thrive in various light environments, why didn’t this diversification occur in the cyanobacteria which can live in almost every niche available on our planet (even at 70-75oC in the hot springs)? Hence a major conclusion of the paper appears to me to be highly speculative, with the only support being the ‘diversification’ itself. It might also be interesting to carefully examine the linkers among all of the reds and see if any of their features do correlate with the physiological characteristics of the different organisms (light, desiccation, life cycle and morphology etc).

The phrase ‘genome reduction’ is indicated as being a ‘period’ to explain the split of Cyanidiophytina. We omitted the word to avoid such confusion: “We speculate that the origin of complex phycobilisome linker structures may have been selected for and thereby supported the radiation of mesophilic rhodophytes into diverse environments. In addition, the phycobilisomes, with their diverse linker proteins enables red algae to adapt to different light environments via chromatic adaptation[48-51]. This dynamic process may partly explain the provenance of the current 7,100 described mesophilic red algal species.”.

In the mesophilic red algae, various cellular life styles (unicellular, filamentous, multicellular, and asexual/sexual), physiological (diverse polysaccharide contents, agar-producing, cell walls, etc), and morphological characteristics were diverged after the diversifications of phycobilisome (PBS) linker proteins in this lineage and the diversified PBS linkers are conserved in genomes of mesophilic red algal groups [Fig. 4 and Supplementary Figure S8]. Although we have no direct evidence to support the correlation between red algal diversification and diversification of PBS-linkers, this PBS diversification could be beneficial to adapt in diverse environments. So, we postulate that the diversification of PBS-linkers could support “the radiation of these taxa into diverse environments”. To emphasize these ideas, we added the sentence in our conclusion: “Among the cyanobacterial phycobilisome-derived eukaryotic lineages (i.e., glaucophytes and rhodophytes), only the mesophilic red algae contain distinct and diversified linker proteins. We speculate that the origin of complex phycobilisome linker structures may have been selected for and thereby supported …”.

Because the diversification of PBS-linkers was a very specific event within the mesophilic red algae, it is hard to reconcile, ‘why didn’t this diversification occur in cyanobacteria?’ similar to ‘why didn’t this occur in glaucophytes?’. If the established gene knock-out/in systems within lineages (mesophilic and extremophilic red algae), between lineages

(rhodophyte and glaucophyte), and between domains (rhodophyte and cyanobacteria) are available, including enough red algal genome data, we may able to generate the data needed to address this question. In general, however, prokaryotes are under persistent selection for genome reduction to counteract genome growth via HGT. Therefore, prokaryote genomes do not reach large sizes and encode small core genomes and extensive accessory gene sets. The latter are gained and lost at high frequency and are usually adaptive. The photosynthetic life style of cyanobacteria, of course results in them maintaining a larger core gene inventory required for this complex function.

Overall, while there is some interesting data in this paper and that various aspects of the evolution of the various photosynthesis genes may be interesting, I feel that the results require more analyses and the major conclusion are speculative with little support. At this point, my opinion is that this work would be a valuable contribution to a more specialized journal, especially if the data was more carefully considered and discussed.

Reviewer #4 (Remarks to the Author):
This paper attempts to clarify how mesophilic red algae diversified. The new data of Porphyridium purpureum is derived from Nanopore long-reads. The hybrid assembly with Illumina reads provides the high quality genome. By molecular phylogenetic analysis using large data sets, the authors show extensive duplications of phycobilisome linker proteins in ancestral mesophilic red algae. They claim that early endosymbiotic gene transfer of the linker protein and the duplication may have played critical roles for the diversification of mesophilic red algae. The finding of expanded linker proteins is novel and seems the most interesting. The paper is clearly written and the results are presented in a clear manner. I'm convinced the conclusions are sufficient novel to warrant publication in Nature Communications. I have some concerns.

Major remarks

L152-L154: The analysis of ancient EGTs is interest to researchers in wider-ranging field. For comparison, some analyses of other TIC/TOC proteins have to indicate using the high quality data sets. In addition, gene trees supporting eukaryotic-specific and land plant-specific origins are useful to interpret the results for ancient EGT origin in Figs. 2, S5 and S6.

To provide a more detailed discussion of ancient EGTs, we moved this section to the Supplementary Text. Instead, we briefly describe the ancient EGTs in the main text, for example: "These differences are explained by differential EGT or outright gene loss in the ancestors of each lineage[8,27]. To generate a more complete picture of the gene loss process, we analyzed EGTs that putatively (i.e., excluding parallel transfers) occurred before the diversification of the three Archaeplastida lineages (Fig. 2 and Supplementary Fig. S4). This analysis shows that genes with several core plastid functions including the TIC/TOC complex and several involved in photosynthesis (PSII, cytochrome, ferredoxin, and ATPase complexes) were moved to the nuclear genomes of primordial algae (Supplementary Table S7, Figs. S5 and S6; see details in Supplementary Text). A remarkable diversification and divergence are found in gene families that encode the light-harvesting antenna complexes…". In the Supplementary Text, we added gene names with their references for several lineage-specific distributions of TIC/TOC genes, and evidence of the eukaryotic TIC/TOC genes (Supplementary Figures S5 and S6); "Other TIC/TOC proteins are eukaryotic-specific (TOC34 and TIC110; Supplementary Figure S5e and S5f)[7,11,12], land plant-specific (TIC55,

note that the green alga *Chlamydomonas reinhardtii* contains 'Rieske iron-sulfur cluster 55 kDa protein of the chloroplast inner membrane translocon; XP_001697980)[7,11], and Viridiplantae-specific (TOC159)[11], or have an ambiguous/unknown provenance[7,11,12].".

L19-L22, L77-80, and L310-L312: The question for diversification of mesophilic red algae is interesting. Comparative genomic analysis by multiple methods indicates new results. But the discussion on the adaptation to diverse environment is ambiguous. Although transcriptome analyses may be beyond the scope of their analysis, the expression data of linker genes in different environments may support the discussion.
We added RNA-seq analysis of phycobilisome linker proteins under different light sources in a novel section (page 13-15) entitled "Potential role of PBS linker proteins in *P. purpureum* chromatic adaptation". We described the growth rate and gene expression patterns based on light sources and concluded that "…These preliminary results lead us to speculate that *P. purpureum* has the capacity to adapt to different light conditions, and specifically to blue light by altering its phycobilisome structure to allow efficient use of phycoerythrin. This and other ideas presented here require more detailed analysis of the CA mechanism in diverse *Porphyridium* species using a combination of comparative genomic, transcriptomic, and biophysical analyses of PBS linker proteins.".

Minor remarks
L77-L80: If the explanation for red algal group, including Proteorhodophytina and Eurhodophytina, is added in the introduction, it is likely to be useful to understand the interesting point of this research.

We highlighted this comment in the introduction as follows; "We determined the origins of phycobilisome linker protein families including novel linker proteins in the mesophilic red algae. Although these taxa show different cellular life styles and plastid genome structures (i.e., subphylum Proteorhodophytina: unicellular/filamentous algae including intron-rich plastid genomes, and Eurhodophytina: macroscopic seaweeds)[24], they share a diversity of conserved, lineage-specific nuclear-encoded phycobilisome linker protein families. Based on these data, we speculate that the ancestor of the mesophilic lineages was under selection to adapt to widely different light environments through the development of flexible phycobilisome structures.".

L97-L99: There is no verb.

We corrected the sentence as "This is a significant improvement from the existing short-read based…".

L130-144: Fig.1 has to be referred in the paragraph.

We revised the part as follows; "The phylogeny was inferred using the maximum likelihood (ML) method with 1,000 bootstrap replications, using the best-fit evolutionary model (Supplementary Table S2 and S6; IQ-tree v1.6.7)[37]. This ML tree provides strong bootstrap support (BS) for a monophyletic Rhodophyta that is sister to the monophyletic groups, Viridiplantae and Glaucophyta (BS = 100%, Fig. 1). The interrelationships of these three primary endosymbiosis groups (i.e., the early split of Rhodophyta) is also supported by transcriptome-based gene and taxon-rich datasets[38] as well as by phylogenetic analysis of plastid genome data[8]. Within Rhodophyta…".

L155-L157 and Figure 2: If the information of conserved photosynthetic proteins in extant cyanobacteria is added, it is helpful to understand the important of the analysis.

We added cyanobacterial copies of the photosynthesis and their antenna complexes in Fig. 2.

L188-L192: I feel that the network analysis with figure 3 needs to explain more clearly.

We added an additional figure plate in Fig. 3 that explains in more detail the biggest network cluster as follows; "This component contains a central node comprising core-membrane linker proteins (LCMs) that connects the LR (rod linker) and LRC (rod-core linker) protein families (Fig. 3; see details in Methods). The LCMs include sequences homologous with the phycobilisome antenna and other linker proteins (Fig. 3a, *e*-value cutoff=1.e-05). These network connections are present when using a more stringent cutoff (*e*-value cutoff=1.*e*-10, Supplementary Fig. S7). The phycobilisome antenna proteins align well with the N-terminal region of LCMs, whereas linker proteins (i.e., LR and LRC) share similarity with the C-terminus of LCMs (Fig. 3b). Although aligned amino acid sequences between the phycobilisome antenna and linker proteins show low overall similarity (*e*-value>1.e-05), these two antenna and linker proteins share several well-conserved amino acid sites (Fig. 3b). Therefore, we used the alignment to construct the phylogeny of LMC-related genes to study the relationships of red algal phycobilisome protein families (Fig. 4).".

L243: Are plastid-targeting signals found in novel Nu-PBS linker sequences?

Most of novel Nu-PBS linker sequences including three novel proteins contain plastid-targeting signals, and we added this information in Supplementary Table S8 and color-coded in Fig. 4.

L379-L381: Does the hybrid genome assembly have accession number of GenBank?

We only provided a data link including genome assembly, gene models, protein sequences of *Porphyridium purpureum*. We are plan to update the genome assembly in GenBank.

Figure 4: "Phycobilisoem" is typo

We corrected this typo in Fig. 4.

Reviewers' comments:

Reviewer #1 (Remarks to the Author):

The revised manuscript now shows a clear focus on phycobilisomes. The section on EGT with respect to photosynthesis genes and TOC/TIC translocon genes was omitted/reduced and a new section on chromatic adaptation was included. The relationship to glaucophytes, the second eukaryotic phylum containg phycobilisomes was pointed out and the general introduction on phycobilisomes was expanded. By that way the excellent data finally meet the presentation they deserve.

Reviewer #2 (Remarks to the Author):

The authors have addressed most of my comments satisfactorily.

Some relatively minor things to tidy up:

1) line 180-181: antenna "pigments" should be antenna proteins or antenna pigment-proteins.

2) Line 194-195: Including long tracks of non-homologous sequences can be problematic. It is fine as it makes a point, but this trends to cluster the the groups that lack the region and those that have them. Depends on how gaps are treated. Not an issue here because the interpretation is okay and the tree makes some important points.

3) Line 220. Figure 4 not really the right reference for this sentence.
4) Line 205-206: This data not on Figure 4, so put (Supplementary Fig S8) at end of that sentence.
5) Line 208-209: "Oxygen-binding and/or transporting functions." This isn't clear, nor is it clear from the figure (S8). Why is it likely to be involved with oxygen binding or transport?
6)Line 310: "we sampled light-harvesting antenna complex". perhaps "we sampled both phycobilisome and light-harvesting complex gene expression."
7) Line 320-321: Growth rate. I'm not sure the growth rate can be considered accurate. It depends on what density they plateau. It seems clear that the growth rate is much slower in red light, but it is difficult to compare the control, high and blue light because they are not significantly different and could have reached max cell density at different times (day 3, 4 or 5). Not something you need to do over, just keep it that in mind. The day 1-5 part is fine, but the growth rate is likely not accurate. Perhaps delete panel b. Haven't grown these myself so you would know better. it is clear that cells don't like being in red light.

8) Line 326-327: Undoubtedly this is true. If you measure chlorophyll a levels, that would be a really good marker as well.

9) Methods: More information on culturing in the experiments. Light intensity, temperature, day-night cycle etc. Sorry if I missed this. How much red light did you use (intensity)?

10) Supplementary figure S15 and S16 (Legend): explain the different panels in the legend so it is clearer. Having some difficulty easily figuring out what each is.

Overall, nice work!

Reviewer #3 (Remarks to the Author):

The authors have made considerable improvements concerning the Porphyridium purpureum nuclear genome assembly and have attempted to reconstructed events associated with EGT and gene

duplications during diversification of mesophyllic red algae, but there were a number of issues with the initial submission. The modified introduction is better, with a little more attention to phycobilisome structure, although it would benefit from a slightly more detailed discussion. Also, now the genes/proteins associated with the chlorophyll a/b antennae of plants and green alga and plastocyanin and proteins involved in plastocyanin function, which are not present in the red algae, are discussed. My first review went into more detail concerning some of the issues. Much of the sequence information seems strong and the findings are interesting, including diversification of the family of genes encoding the linker polypeptides. However, the real novelty of the findings would be much stronger if a connection between linker structure/composition and function were established (e.g. based on the different environments in which the various red algae thrive). The argument concerning chromatic adaptation and adaptation to the light conditions is valid, but many cyanobacteria also strongly chromatically adapt and they don't have the same types of phycobilisomes as the red algae (many cyanobacteria can reduce the PC and increase the PE in the rods in green light and switch back to PC dominant rods in red light; or change the size of the PBS rods depending on the light intensity). Therefore, I am not sure that chromatic adaptation really has to do with strictly with linker diversification in the reds (although there would be some differences in linkers used under the different light conditions that would be specific to the different phycobiliprotein hexamers synthesized/modified under those conditions).
Also, the new work presented on expression is a little confusing to me. To do this type of analyses it would be important to know whether the PBS composition and absorption characteristics of the photosynthetic pigments are changing during growth in high light (the authors call 30 μmol photons m-2s-1 high light; this is still low light), blue light and red light (what are the μmol photons m-2s-1 of the blue and red light and what is the half maximum band width?). A crude action spectrum for chromatic adaptation (absorption spectra after growth in different qualities and intensities of light) would roughly show the best light conditions for eliciting chromatic adaptation and the ways in which the absorption characteristics of the organism are changing under the different light conditions (has this been done by someone already?). What is the nature of chromatic adaptation in P. purpureum? Is it just changing the chromophore on the PE subunits to give a somewhat different PE absorption spectrum? Do the different light conditions change the ratio of PE to PC to chlorophyll? Were the wavelengths in red (628) and blue (463) light chosen because only the PE absorbs the blue light (usually it best absorbs green light) and only the PC absorbs the red light? I was not even sure what the rationale was for the way in which the experiment was performed to find linkers associated with different light acclimation states. Why not grow the cells in red light (extreme acclimation to red light… of various intensities) and then switch to blue light (of various intensities). The parallel experiment would involve switching the cells from growth in blue light to red light… growth rates may increase following the switch as the cells acclimate (this would take some time, so there may be a lag) and begin to change the pigment composition of their PBS (and potentially also the levels of chlorophyll and carotenoids in their photosynthetic membranes)… changes in absorption spectra may correlate with a changes in PBS compositions and levels of specific linkers (and the levels of their transcripts). It does seem like the levels of three linker transcripts were higher in blue than in red light (by about 2 fold). These transcripts could be quantified by RT-qPCR (not necessarily RNA-seq) with many replicates… to get stronger data with standard errors. Overall, the background concerning CA in P. purpureum is not given, making it difficult to know what to expect, and the expression experiments presented are both a bit confusing and do not seem optimal with respect to experimental design or analyses of the data.

Reviewer #4 (Remarks to the Author):

The authors revised the manuscript according to reviewers' comments. They added RNA-seq analysis in different lighting condition and the discussions are revised clearly. I just have one concern that accession numbers for the hybrid genome assembly are not available on NCBI database.

The point-by-point responses

Reviewers' comments:

Reviewer #1 (Remarks to the Author):
The revised manuscript now shows a clear focus on phycobilisomes. The section on EGT with respect to photosynthesis genes and TOC/TIC translocon genes was omitted/reduced and a new section on chromatic adaptation was included. The relationship to glaucophytes, the second eukaryotic phylum containg phycobilisomes was pointed out and the general introduction on phycobilisomes was expanded. By that way the excellent data finally meet the presentation they deserve.

Thank you for this positive response.

Reviewer #2 (Remarks to the Author):
The authors have addressed most of my comments satisfactorily.
Some relatively minor things to tidy up:
1) Line 180-181: antenna "pigments" should be antenna proteins or antenna pigment-proteins.

We changed “antenna pigments” to “antenna pigment proteins”.

2) Line 194-195: Including long tracks of non-homologous sequences can be problematic. It is fine as it makes a point, but this trends to cluster the groups that lack the region and those that have them. Depends on how gaps are treated. Not an issue here because the interpretation is okay and the tree makes some important points.

We thank the reviewer for this important comment.

3) Line 220. Figure 4 not really the right reference for this sentence.

We revised the sentence as follows (line 202); “In addition, phycobilisomes of Cyanidiophytina lack phycoerythrin that is absent not only in genome data (PE alpha and beta in Fig. 4) but also when studied using fluorescence spectroscopy[43,44].”.

4) Line 205-206: This data not on Figure 4, so put (Supplementary Fig S8) at end of that sentence.

We added “Supplementary Fig. S8” at end of the sentence as follows: “…we extend this result by showing that it is present in other red algae and in some cryptophytes (Supplementary Fig. S8).”

5) Line 208-209: "Oxygen-binding and/or transporting functions." This isn't clear, nor is it clear from the figure (S8). Why is it likely to be involved with oxygen binding or transport?

We deleted the unclear part as follows: “Because of the well-supported relationship with cyanobacteria that contain allophycocyanin-like globin domains (cd12130), this novel

phycobilisome protein is likely to be involved in allophycocyanin-related functions (Supplementary Fig. S8) and contains a globin superfamily-like domain superfamily (cl21461).”

6) Line 310: "we sampled light-harvesting antenna complex". perhaps "we sampled both phycobilisome and light-harvesting complex gene expression."

We revised the sentence as follows: “We studied both phycobilisome and light-harvesting complex gene expression using normalized values…”

7) Line 320-321: Growth rate. I'm not sure the growth rate can be considered accurate. It depends on what density they plateau. It seems clear that the growth rate is much slower in red light, but it is difficult to compare the control, high and blue light because they are not significantly different and could have reached max cell density at different times (day 3, 4 or 5). Not something you need to do over, just keep it that in mind. The day 1-5 part is fine, but the growth rate is likely not accurate. Perhaps delete panel b. Haven't grown these myself so you would know better. it is clear that cells don't like being in red light.

Thank you for this comment. In response, we have deleted the growth rate panel in Supplementary Fig. S13.

8) Line 326-327: Undoubtedly this is true. If you measure chlorophyll a levels, that would be a really good marker as well.

We thank you for raising this important point. We will consider this experiment in future studies.

9) Methods: More information on culturing in the experiments. Light intensity, temperature, day-night cycle etc. Sorry if I missed this. How much red light did you use (intensity)?

We added this information to the Methods in Supplementary Text.

10) Supplementary figure S15 and S16 (Legend): explain the different panels in the legend so it is clearer. Having some difficulty easily figuring out what each is.

We added detailed legends to the panels in Supplementary Fig. S15 and S16.

Overall, nice work!

We appreciate your valuable comments.

Reviewer #3 (Remarks to the Author):

The authors have made considerable improvements concerning the Porphyridium purpureum nuclear genome assembly and have attempted to reconstructed events associated with EGT and gene duplications during diversification of mesophyllic red algae, but there were a number of issues with the initial submission. The modified introduction is better, with a little more attention

to phycobilisome structure, although it would benefit from a slightly more detailed discussion. Also, now the genes/proteins associated with the chlorophyll a/b antennae of plants and green alga and plastocyanin and proteins involved in plastocyanin function, which are not present in the red algae, are discussed.

My first review went into more detail concerning some of the issues. Much of the sequence information seems strong and the findings are interesting, including diversification of the family of genes encoding the linker polypeptides. However, the real novelty of the findings would be much stronger if a connection between linker structure/composition and function were established (e.g. based on the different environments in which the various red algae thrive). The argument concerning chromatic adaptation and adaptation to the light conditions is valid, but many cyanobacteria also strongly chromatically adapt and they don't have the same types of phycobilisomes as the red algae (many cyanobacteria can reduce the PC and increase the PE in the rods in green light and switch back to PC dominant rods in red light; or change the size of the PBS rods depending on the light intensity). Therefore, I am not sure that chromatic adaptation really has to do with strictly with linker diversification in the reds (although there would be some differences in linkers used under the different light conditions that would be specific to the different phycobiliprotein hexamers synthesized/modified under those conditions).

Also, the new work presented on expression is a little confusing to me. To do this type of analyses it would be important to know whether the PBS composition and absorption characteristics of the photosynthetic pigments are changing during growth in high light (the authors call 30 µmol photons m-2s-1 high light; this is still low light), blue light and red light (what are the µmol photons m-2s-1 of the blue and red light and what is the half maximum band width?). A crude action spectrum for chromatic adaptation (absorption spectra after growth in different qualities and intensities of light) would roughly show the best light conditions for eliciting chromatic adaptation and the ways in which the absorption characteristics of the organism are changing under the different light conditions (has this been done by someone already?). What is the nature of chromatic adaptation in P. purpureum? Is it just changing the chromophore on the PE subunits to give a somewhat different PE absorption spectrum? Do the different light conditions change the ratio of PE to PC to chlorophyll? Were the wavelengths in red (628) and blue (463) light chosen because only the PE absorbs the blue light (usually it best absorbs green light) and only the PC absorbs the red light? I was not even sure what the rationale was for the way in which the experiment was performed to find linkers associated with different light acclimation states. Why not grow the cells in red light (extreme acclimation to red light… of various intensities) and then switch to blue light (of various intensities). The parallel experiment would involve switching the cells from growth in blue light to red light… growth rates may increase following the switch as the cells acclimate (this would take some time, so there may be a lag) and begin to change the pigment composition of their PBS (and potentially also the levels of chlorophyll and carotenoids in their photosynthetic membranes)… changes in absorption spectra may correlate with a changes in PBS compositions and levels of specific linkers (and the levels of their transcripts). It does seem like the levels of three linker transcripts were higher in blue than in red light (by about 2 fold). These transcripts could be quantified by RT-qPCR (not necessarily RNA-seq) with many replicates… to get stronger data with standard errors. Overall, the background concerning CA in P. purpureum is not given, making it difficult to know what to expect, and the expression experiments presented are both a bit confusing and

do not seem optimal with respect to experimental design or analyses of the data.

We truly the appreciate the depth of detail and thought that Rev. 3 has provided regarding testing hypotheses about CA in *Porphyridium*, and the design of experiments to achieve this aim. However, analysis of the structure/composition of phycobilisome linker proteins requires biophysical analyses that are beyond the goals of our manuscript and our team's abilities. In fact, we are very interested in launching such a comprehensive study, as described in Zhang et al. (2017, *Nature*), to address the structure/composition of phycobilisome linker proteins. The current work does not allow such in-depth inferences to be made.

To address other issue raised by Rev. 3, we make clear in the revised manuscript that the relationship between phycobilisome linkers and chromatic adaptation (CA) in *P. purpureum* remains unclear. It may be different from cyanobacterial CA. However, **the major function of phycobilisome linker proteins in *P. purpureum* is generally thought to be structural maintenance of phycobilisomes. This role implicates a variety of potentially adaptive functions (e.g., pH-dependent functional stability), although CA may not be among them.** We have previously noted that this area requires more detailed analysis: e.g., "This and other ideas presented here require more detailed analysis of the CA mechanism in diverse *Porphyridium* species using a combination of comparative genomic, transcriptomic, and biophysical analyses of PBS linker proteins.".

Moreover, the diversification of phycobilisome linker proteins is a red algal-specific event as we have already discussed. At this point, the red algal-specific patterns are not directly comparable to cyanobacterial phycobilisome linker proteins.

Please note that the RNA-seqs experiments were originally suggested by Reviewer 4 as a means to test if there was different expression patterns of genes encoding linker proteins under different light environments (see the original comments as follows): "L19-L22, L77-80, and L310-L312: The question for diversification of mesophilic red algae is interesting. Comparative genomic analysis by multiple methods indicates new results. But the discussion on the adaptation to diverse environment is ambiguous. Although transcriptome analyses may be beyond the scope of their analysis, the expression data of linker genes in different environments may support the discussion."

Based on these valuable comments, we chose to add the RNA-seq experiments and selected blue and red light as treatment conditions to generate the data. This choice is based on previous CA experiment with *Porphyridium cruentum* (Ley and Butler 1980, *Plant Physiology*), in which red and blue light wavelengths revealed optimized light absorptions for phycoerythrin and phycocyanin, respectively. The action spectrums of our light wavelength experiments are shown in the new Supplementary Fig. S13a.

As a result of the RNA-seq experiment, however, we found no evidence for significant differences in gene expression of PBS linker proteins under the conditions we tested. Therefore, we surmise that *P. purpureum* likely has the capacity to adapt to different light environments, and specifically to blue light.

The light-dependent change of the ratio in phycobilisome components in *P. purpureum* remains unclear, as we have suggested. Potentially, CA in *P. purpureum* could be primarily regulated by other cellular mechanisms not connected to the light-harvesting complex.

In summary, our work was designed to study the evolutionary history of phycobilisome linker gene families, and we added the RNA-seq work (based on Rev. 4's comments) and introduce CA to generate functional data to test our broad hypotheses. We recognize however that these latter results are provisional, but apparently of value, as the other three anonymous reviewers have clearly pointed out. Therefore, we consider detailed analysis of CA in *Porphyridium* to comprise a different research topic that is outside the major focus of our manuscript.

Reviewer #4 (Remarks to the Author):

The authors revised the manuscript according to reviewers' comments. They added RNA-seq analysis in different lighting condition and the discussions are revised clearly. I just have one concern that accession numbers for the hybrid genome assembly are not available on NCBI database.

We appreciate this comment and will upload the improved, hybrid *Porphyridium* genome assembly to GenBank immediately upon acceptance of the manuscript.

Reviewers' comments:

Reviewer #3 (Remarks to the Author):

Again, while I think that there is a lot of valuable information in this paper, it still suffers from some difficulties with precision and physiological/biochemical implications of the results.

The light intensities used are very low and there may be enough chlorophyll antenna to absorb all of it (5 umol photons m-2 s-1 for red and blue and 30 for what is considered high light). All of this is very low light so I don't know how meaningful it will be with respect to chromatic adaptation.

There are still places where discussion of the PBS and linkers are confusing. The linkers may be involved in both stability and tuning the absorption characteristics of the rods to create highly efficient energy transfer from the peripheral hexamers of the rods to the PBS core and then to the PSII reaction center.

LR2, LR3 and LRC5 transcripts are somewhat down (2 fold) in red light relative to blue light so the authors say that they are associated with phycoerythrin. I don't think that this can be said based on the expression data... it has to be shown in a more credible way. Also, is the expression of phycoerythrin ad phycocyanin altered at all in red and blue light?

From what I can tell figure S13 does not show an action spectrum. I think it is just the spectral output from the different light sources (white light, HL, RL and BL). An action spectrum for photosynthesis might show the different effects of specific wavelengths of light (ranging from the blue to the red) on photosynthetic activity (e.g. oxygen evolution) on cells grown in red light or blue light. This would demonstrate that the effectiveness of the different light qualities on photosynthetic activity changes when the cells are grown under red and blue light conditions. But even just characterizing the absorption spectra of cells grown in red light and blue light might show that the different wavelengths of light used for growth alter their absorption spectra in accord with what would be expected for chromatic adaptation.

Reviewer #4 (Remarks to the Author):

If the hybrid data will be registered, I will be satisfied with the revised version.

Point-by-point responses

Reviewers' comments:

Reviewer #3 (Remarks to the Author):

Again, while I think that there is a lot of valuable information in this paper, it still suffers from some difficulties with precision and physiological/biochemical implications of the results.

The light intensities used are very low and there may be enough chlorophyll antenna to absorb all of it (5 umol photons m-2 s-1 for red and blue and 30 for what is considered high light). All of this is very low light so I don't know how meaningful it will be with respect to chromatic adaptation.

There are still places where discussion of the PBS and linkers are confusing. The linkers may be involved in both stability and tuning the absorption characteristics of the rods to create highly efficient energy transfer from the peripheral hexamers of the rods to the PBS core and then to the PSII reaction center.

LR2, LR3 and LRC5 transcripts are somewhat down (2 fold) in red light relative to blue light so the authors say that they are associated with phycoerythrin. I don't think that this can be said based on the expression data... it has to be shown in a more credible way. Also, is the expression of phycoerythrin ad phycocyanin altered at all in red and blue light?

From what I can tell figure S13 does not show an action spectrum. I think it is just the spectral output from the different light sources (white light, HL, RL and BL). An action spectrum for photosynthesis might show the different effects of specific wavelengths of light (ranging from the blue to the red) on photosynthetic activity (e.g. oxygen evolution) on cells grown in red light or blue light. This would demonstrate that the effectiveness of the different light qualities on photosynthetic activity changes when the cells are grown under red and blue light conditions. But even just characterizing the absorption spectra of cells grown in red light and blue light might show that the different wavelengths of light used for growth alter their absorption spectra in accord with what would be expected for chromatic adaptation.

In response, originally, we wanted to test the gene expression patterns of phycobilisome linker proteins under different light conditions, as we have previously explained (suggested by Reviewer 4) to explore the topic of ‘chromatic adaptation’ in *P. purpureum*. Therefore, we addressed this issue in a preliminary fashion because it was not the main focus of our manuscript. As Reviewer 3 points out however, the experiments we did have some limitations, such as the use of lower light levels when compared to standard experiments down with plants. Therefore, we have excluded discussion of chromatic adaptation from the main text and moved this section, now entitled, ‘Gene expression patterns of PBS linker proteins in *P. purpureum* under different light conditions’, to the Supplementary Text (pages 2-6), as originally suggested by Reviewer 4. We recognize that a meaningful analysis of chromatic adaptation requires a different study plan (e.g., comparative genomic, transcriptomic, and biophysical analyses using diverse *Porphyridium* species) as Reviewer 3 suggests, that is not addressed by our research.

In general, differential gene expression (DEG) analysis provides an useful tool for generating a candidate list of genes that act under different experimental conditions. Our RNA-seq approach is the first to attempt this work for mesophilic red algal phycobilisome linker proteins using different light sources. We have interpreted these preliminary data in the Supplementary Text (pages 5-6) as follows: "Although many PBS linker proteins showed a response to the different light conditions we tested, no PBS linker gene was significantly up-regulated when compared to the control. We postulate that the organization of PBS liker proteins aids in maintenance of the structural integrity and stability of phycobilisome complexes in mesophilic red algae. To gain greater insight into this process, we suggest comparative genomic, transcriptomic, and biophysical analyses of PBS linker proteins as well as analysis of other photosynthetic pigments in a diversity of *Porphyridium* species.".

We have already described the expression patterns of chlorophyll-related genes (e.g., chlorophyll *a*/*b* binding, *psa*- and *psb*-gene families) in the Supplemental Text but these do not provide evidence of significant gene expression differences among chlorophyll-related genes. We added this point as follows: "To gain greater insight into this process, we suggest comparative genomic, transcriptomic, and biophysical analyses of PBS linker proteins as well as analysis of other photosynthetic pigments in a diversity of *Porphyridium* species.".

The light intensity analysis is comparative, therefore we changed the term 'high light' to 'higher light'.

The action spectrum of *P. purpureum* under different light conditions was already done by Ley and Butler (1980, *Plant Physiol*.) but we excluded this section, and described only the gene expression responses of PBS linker proteins.

Thank you for your valuable comments.

Reviewer #4 (Remarks to the Author):

If the hybrid data will be registered, I will be satisfied with the revised version.
We will upload after acceptance of our manuscript in this journal.

REVIEWERS' COMMENTS:

Reviewer #3 (Remarks to the Author):

Once again, I feel that much of the information in the manuscript is strong and worth reporting. The connection between radiation of the red algae and diversification of the linker proteins is still only a correlation with no physiological or molecular supporting data and no strong exploration of this aspect of the analyses. The additional data included on transcript accumulation for the linkers under the different light conditions is somewhat worthwhile, but it doesn't add to the argument being made by the authors, and I am not convinced that it even addresses the question given that both conditions appear to be low white light, even though one is 3X the other (and low and higher light conditions would have the same balance of blue and red light)… but it is now in supplementary data. I also still had some problem understanding what was written… specifically,

‘To collect RNA-seq data, we initially cultured P. purpureum under white light LED (10 μmol photons m^{-2} s^{-1}) as control for one week, and then exposed to the higher (30 μmol photons m^{-2} s^{-1}), blue (peak wavelength = 463 nm; 5 μmol photons m^{-2} s^{-1}), and red (peak wavelength = 628 nm; 5 μmol photons m^{-2} s^{-1}) light conditions (Supplementary Fig. S13a; SPIC-200AW, Everfine Co. Hangzhou, China) for five days at 20°C.�Ꞌ

I take it that the authors mean "... and then exposed the cultures to higher white light (30 μmol photons m^{-2} s^{-1})...'

It sounds like in the higher white light the peak has 5 μmol photons m-2 s-1 blue light and the same for red light, but that would be over a specific set of wavelengths… and not just the peak (e.g. maybe from 450-475 nm for the blue light) etc. I am sure that that the authors can clarify these points.

There is important sequence information provided in the paper and the correlation between linker diversification and radiation of mesophilic red algae is interesting, but it is not enough to make a strong causal argument (it is given as speculative). And it would need more physiological, biochemical and molecular work to strengthen the argument. Therefore, while I do have some reservations, I will defer to the other reviewers and the editor.

Point-by-point responses

REVIEWERS' COMMENTS:
Reviewer #3 (Remarks to the Author):

Once again, I feel that much of the information in the manuscript is strong and worth reporting. The connection between radiation of the red algae and diversification of the linker proteins is still only a correlation with no physiological or molecular supporting data and no strong exploration of this aspect of the analyses. The additional data included on transcript accumulation for the linkers under the different light conditions is somewhat worthwhile, but it doesn't add to the argument being made by the authors, and I am not convinced that it even addresses the question given that both conditions appear to be low white light, even though one is 3X the other (and low and higher light conditions would have the same balance of blue and red light)… but it is now in supplementary data. I also still had some problem understanding what was written… specifically,

‘To collect RNA-seq data, we initially cultured *P. purpureum* under white light LED (10 µmol photons m−2 s−1) as control for one week, and then exposed to the higher (30 µmol photons m−2 s−1), blue (peak wavelength = 463 nm; 5 µmol photons m−2 s−1), and red (peak wavelength = 628 nm; 5 µmol photons m−2 s−1) light conditions (Supplementary Fig. S13a; SPIC-200AW, Everfine Co. Hangzhou, China) for five days at 20°C.‘

I take it that the authors mean "... and then exposed the cultures to higher white light (30 µmol photons m−2 s−1)...'

It sounds like in the higher white light the peak has 5 µmol photons m-2 s-1 blue light and the same for red light, but that would be over a specific set of wavelengths… and not just the peak (e.g. maybe from 450-475 nm for the blue light) etc. I am sure that that the authors can clarify these points.

As we have discussed previously, the purpose of these experiments was to determine gene expression responses of phycobilisome linker protein families. Although typical white light LED contains a high proportion of blue light, the particular treatment conditions (e.g., blue, red light) were limited to these wavelengths. In addition, we excluded the discussion of ‘chromatic adaptation’. To clarify these points, we added the following sentence in the Supplementary Information: “…for five days at 20°C. We used the typical LED white light source as the control without any modification (e.g., decreasing the blue light amount) because the purpose of our experiment was to determine the gene expression responses of phycobilisome linker protein families when the cells were exposed to the specific wavelengths of red and blue light. We recognize however that it has been found in land plants that the high proportion of blue light in the typical white light LED source can impact gene expression levels resulting in altered phenotypes (e.g., ascorbate content)[27]. All of these experiments…”.

There is important sequence information provided in the paper and the correlation between linker diversification and radiation of mesophilic red algae is interesting, but it is not enough to make a strong causal argument (it is given as speculative). And it would need more physiological,

biochemical and molecular work to strengthen the argument. Therefore, while I do have some reservations, I will defer to the other reviewers and the editor.

We revised the title, abstract, and conclusion to tone down the argument regarding the correlation between linker diversification and radiation of mesophilic red algae as follows.
1) Title: 'Expansion of phycobilisome linker gene families in mesophilic red algae'
2) Abstract: "We speculate that the origin of these complex families in mesophilic red algae may have contributed to their adaptation to a diversity of environments."
3) Conclusion: "We speculate that the origin of complex phycobilisome linker structures may reflect selection to expand light harvesting capacity during the radiation of mesophilic red algae (7,100 described species) into a variety of aquatic environments such as the high and low intertidal, subtidal, and in association with coral reefs."

Thank you for your comments.
